# RNA Sequencing of Sessile Serrated Colon Polyps Identifies Differentially Expressed Genes and Immunohistochemical Markers

**DOI:** 10.1371/journal.pone.0088367

**Published:** 2014-02-12

**Authors:** Don A. Delker, Brett M. McGettigan, Priyanka Kanth, Stelian Pop, Deborah W. Neklason, Mary P. Bronner, Randall W. Burt, Curt H. Hagedorn

**Affiliations:** 1 Department of Medicine, University of Utah, Salt Lake City, Utah, United States of America; 2 Department of Pathology, University of Utah, Salt Lake City, Utah, United States of America; 3 Huntsman Cancer Institute, University of Utah, Salt Lake City, Utah, United States of America; 4 University of Arkansas for Medical Sciences, Little Rock, Arkansas, United States of America; 5 The Central Arkansas Veterans Healthcare System, Little Rock, Arkansas, United States of America; University of Munich, Germany

## Abstract

**Background:**

Sessile serrated adenomas/polyps (SSA/Ps) may account for 20–30% of colon cancers. Although large SSA/Ps are generally recognized phenotypically, small (<1 cm) or dysplastic SSA/Ps are difficult to differentiate from hyperplastic or small adenomatous polyps by endoscopy and histopathology. Our aim was to define the comprehensive gene expression phenotype of SSA/Ps to better define this cancer precursor.

**Results:**

RNA sequencing was performed on 5′ capped RNA from seven SSA/Ps collected from patients with the serrated polyposis syndrome (SPS) versus eight controls. Highly expressed genes were analyzed by qPCR in additional SSA/Ps, adenomas and controls. The cellular localization and level of gene products were examined by immunohistochemistry in syndromic and sporadic SSA/Ps, adenomatous and hyperplastic polyps and controls. We identified 1,294 differentially expressed annotated genes, with 106 increased ≥10-fold, in SSA/Ps compared to controls. Comparing these genes with an array dataset for adenomatous polyps identified 30 protein coding genes uniquely expressed ≥10-fold in SSA/Ps. Biological pathways altered in SSA/Ps included mucosal integrity, cell adhesion, and cell development. Marked increased expression of *MUC17*, the cell junction protein genes *VSIG1* and *GJB5*, and the antiapoptotic gene *REG4* were found in SSA/Ps, relative to controls and adenomas, were verified by qPCR analysis of additional SSA/Ps (n = 21) and adenomas (n = 10). Immunohistochemical staining of syndromic (n≥11) and sporadic SSA/Ps (n≥17), adenomatous (n≥13) and hyperplastic (n≥10) polyps plus controls (n≥16) identified unique expression patterns for VSIG1 and MUC17 in SSA/Ps.

**Conclusion:**

A subset of genes and pathways are uniquely increased in SSA/Ps, compared to adenomatous polyps, thus supporting the concept that cancer develops by different pathways in these phenotypically distinct polyps with markedly different gene expression profiles. Immunostaining for a subset of these genes differentiates both syndromic and sporadic SSA/Ps from adenomatous and hyperplastic polyps.

## Introduction

Colon cancer is the second leading cause of cancer deaths in the United States, with more than 100,000 new cases diagnosed yearly [1], [2]. Primary prevention involves screening colonoscopy every ten years for those ≥50 years of age and more frequently for individuals with first degree relatives with colon cancer or specific types of colonic polyps [2], [3]. Recent studies provide evidence that a subset of serrated polyps, sessile serrated adenomas/polyps (SSA/Ps), which remain inadequately defined but generally have a flat phenotype with a mucus cap, right colonic predominance and a distinctive serrated morphology, may account for 20–30% of colon cancers [3]–[8]. Although large right-sided SSA/Ps generally have a typical phenotype, differentiating small (<1 cm) or dysplastic SSA/Ps from simple hyperplastic or adenomatous polyps can be difficult and histological diagnosis has high observer variability [6], [9]. Improved diagnostics for SSA/Ps are greatly needed to better identify which lesions confer a colorectal cancer risk to inform appropriate cancer screening and prevention care of patients [3].

The Serrated Polyposis Syndrome (SPS) is an exaggerated serrated polyp phenotype exhibiting multiple SSA/Ps with a high risk of colon cancer [3], [4]. Two studies of at least 100 patients with SPS indicated a 25%–42% increased risk for colon cancer [10], [11]. Although an increased risk of colon cancer in relatives of patients with SPS has been suggested [10], the great majority of SPS cases do not appear to be hereditary. Preliminary studies have suggested that the pathway of progression to colon cancer in SSA/Ps is different than that in adenomatous polyps [9], [11]–[13]. An increased rate of BRAF mutations and CpG island hypermethylation has been reported in SSA/Ps as compared to adenomatous polyps [14], [15]. In addition, DNA microsatellite instability has been reported in a subset of colon cancers originating from SSA/Ps [16], [17]. Environmental factors, including cigarette smoking, have been suggested to contribute to the pathogenesis of both syndromic and sporadic SSA/Ps [18], [19]. Patients with SPS provided a unique opportunity to study the molecular phenotype of SSA/Ps because of their numerous polyps, thus enabling biospecimens to be collected more rapidly.

Serrated polyps have been classified into three groups; hyperplastic polyps (HP), sessile serrated adenoma/polyp (SSA/P), and traditional serrated adenoma (TSA) [3], the latter representing rare lesions. Hyperplastic polyps (HP) may be histologically further subdivided into microvesicular hyperplastic polyps (MVHP), goblet cell hyperplastic polyps (GCHP) and mucin-poor hyperplastic polyps (MPHP); with MVHP being the predominant type [3]. However, relatively few studies use this subclassification of HPs and a recent panel of experts suggested not subclassifying HPs [20], [21]. Morphology alone is an insufficient diagnostic standard for identifying serrated polyps with a cancer risk, emphasizing the need for better markers and criteria to accurately diagnose SSA/Ps [20], [22], [23]. Two reports have provided an initial gene expression phenotype of sporadic SSA/Ps [24], [25]. One study used arrays that interrogated expression of 16,000 genes and compared SSA/Ps to pooled mRNA from four unmatched control colon specimens [24]. Another study analyzed RNA from formalin fixed paraffin embedded samples, known to have major RNA decay challenges, and used three unmatched control specimens [25]. In this study we used RNA sequencing (RNA-seq) analysis [26]–[28], because it detects a greater range of changes in expressed genes and more details of transcripts, to define the comprehensive expressed transcriptome of prospectively collected SSA/Ps in SPS patients as compared to patient matched uninvolved and normal colonic mucosa. In addition, we analyzed 5′ capped RNA that identifies more differentially expressed Pol II genes [29]. Our aim was to establish and mine an RNA-seq database of highly expressed genes in SSA/Ps followed by confirmatory qPCR and immunohistochemistry analysis to identify candidate markers that differentiate SSA/Ps from adenomas and HPs.

## Methods

### Patients

**Figure 1:**
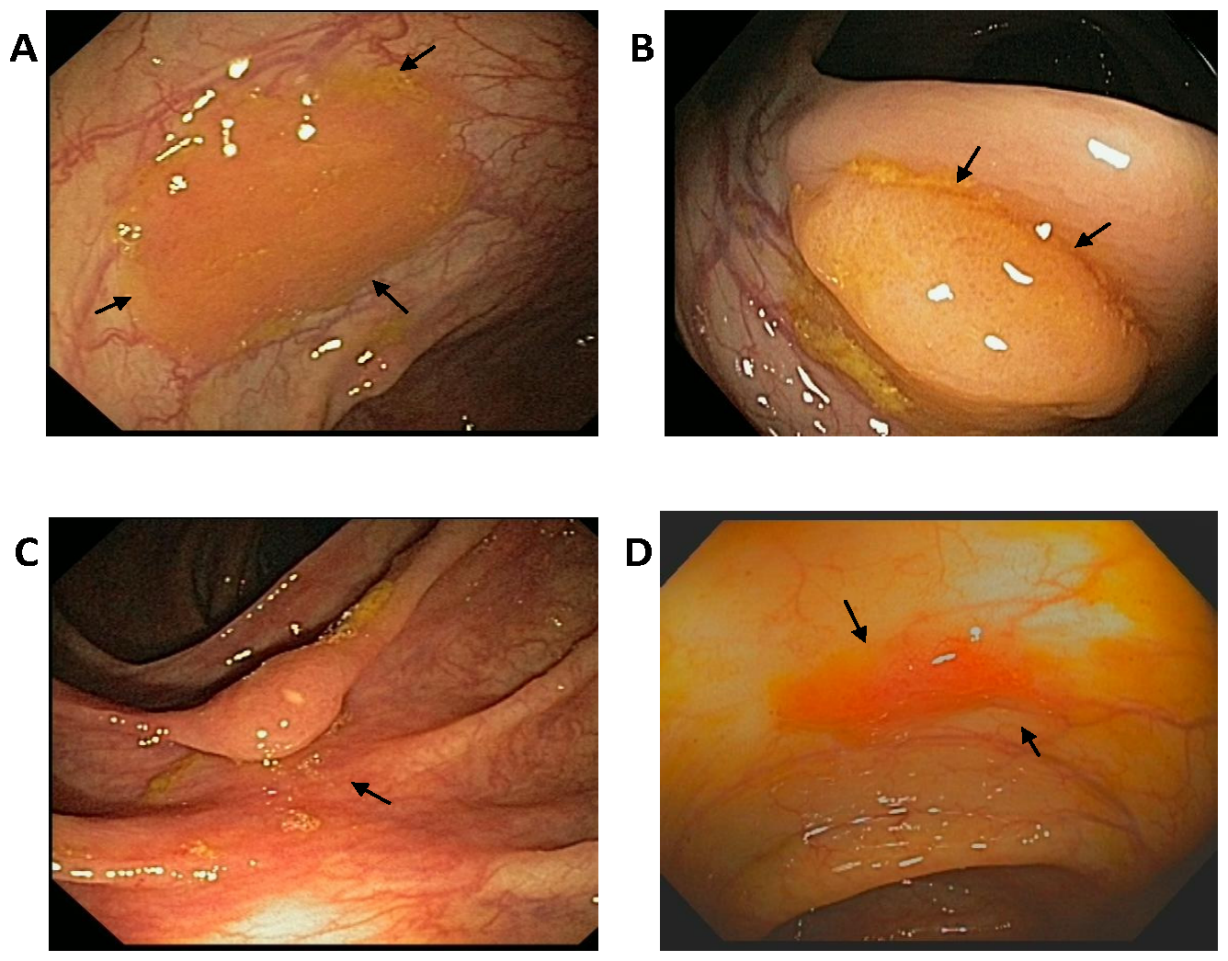
Endoscopic phenotype of four representative sessile serrated polyps/adenomas (SSA/Ps) located in the ascending colon of patients with the serrated polyposis syndrome. **Panel A**. Large 15 mm diameter SSA/P with a mucus cap. **Panel B**. 20 mm diameter SSA/P. **Panel** C. 10 mm diameter SSA/P. **Panel D**. Small 4 mm diameter SSA/P. The size of polyps was estimated using biopsy forceps as a reference. Histopathology analyses were consistent with SSA/Ps (M. Bronner).

All participants provided their written informed consent to participate in this study and all research, including the consent procedure, was approved by the Institutional Review Board (IRB). Approved University of Utah IRB protocols that were relevant to this study included: IRB 00040131, Characterizing the Molecular Signatures of Rare Inherited Colon Cancer Syndromes for Diagnosis and Intervention, IRB 00062125; Molecular Markers of Colon Cancer, IRB 00051140; Molecular Markers of Sporadic Hyperplastic Colon Polyps, IRB 00005829; High Risk Familial Colon Cancer: Genetics and Phenotype. SSA/P and patient matched surrounding uninvolved right colonic biopsy specimens were prospectively collected from 2008–2012 for gene expression analysis from eleven patients with the serrated polyposis syndrome (SPS) seen at the Huntsman Cancer Institute (see Table 1 and Figure 1 for details). All polyps (n = 21, 10≥1 cm) were collected from the cecum to the splenic flexure (designated right colon) of patients. Normal control colon (right colon; n = 10; screening colonoscopy and no polyps) and adenomatous polyps (n = 10; 5–10 mm diameter; right sided; from seven patients) were collected from patients undergoing routine screening colonoscopy (see Table S1 in File S1 for demographics). Biopsy specimens were placed in RNAlater (Invitrogen) immediately following collection and stored at 4°C overnight prior to total RNA isolation the following day. cDNA from the same SSA/Ps of SPS patients used for RNA analysis and from hyperplastic polyps (HPs, n = 10, 3–9 mm diameter) prospectively collected from the left colon of five SPS patients were used for BRAF mutation analysis. Retrospectively collected samples of formalin-fixed paraffin-embedded (FFPE) tissue of “syndromic” SSA/Ps from our SPS patient cohort were analyzed by immunohistochemistry. Sporadic SSA/Ps, HPs, adenomas and uninvolved or control colon were collected from 55 patients, without familial risk of colon cancer or SPS, undergoing surveillance or screening colonoscopy exams. These patients were between 46 and 78 years of age and were seen between 2009 and 2012.

**Table 1:** Demographics of Serrated Polyposis Syndrome Patients.

#	Sex	Age of Diagnosis	Smoking	Indication for Colonoscopy	Total # of Colonoscopies	Total # of Polyps	# Proximal Polyps	% Proximal Polyps	# of Large Polyps (>1cm)		FH Colon Cancer
**1**	M	62	Never	FH CRC	5	68	49	72	7		Yes
**2**	M	33	Never	Hematochezia	5	38	14	36	0		Yes
**3**	F	24	Never	Diarrhea	7	33	16	48	7		No
**4**	F	28	Never	Hematochezia	3	18	14	77	5		No
**5**	M	18	Never	Abd pain	6	91	22	24	0		No
**6**	F	26	Current	Hematochezia	6	67	54	80	0		No
**7**	M	51	Current	Screening	2	15	10	66	7		Yes
**8**	M	71	Ex-smoker	Screening	6	81	28	34	0		Yes
**9**	M	27	Ex-smoker	Hematochezia	2	44	8	18	1		No
**10**	M	25	Ex-smoker	Hematochezia	2	30	19	63	2		No
**11**	F	27	Never	FH CRC	3	23	10	43	1		Yes

History and colonoscopy details of patients with serrated polyposis syndrome. Only polyps with the serrated histopathology are reported. None of the patients had colon cancer. FH = Family History.

### Pathologic Classification

All biopsy specimens were collected from the cecum to the splenic flexure (designated right colon) and reviewed by an expert GI pathologist and details of the SSA/Ps (e.g., size and location) were recorded (Table S2 in File S1). Serrated polyps were classified according to the recent recommendations of the Multi-Society Task Force on Colorectal Cancer for post-polypectomy surveillance that recommended classifying serrated lesions into hyperplastic polyps without subtypes, SSA/P with and without dysplasia, and traditional serrated adenomas (TSAs) that are relatively rare [21]. The criteria for selecting unequivocal SSA/Ps for RNA profiling were that they were from the right colon and had two or more of the following, size >1 cm, morphologic features of predominantly dilated serrated crypts extending to the mucosal base and/or had dysmaturation of crypts. This selection criteria for SSA/Ps by no means implies that SSA/P's are limited to the right colon. It was simply a means to enrich the likelihood we were analyzing classic and clear cut SSA/Ps. Serrated polyps with a size <1 cm, not meeting these criteria and from the left colon were designated hyperplastic polyps without further subtyping. Only one HP analyzed was > than 6 mm. Again, these study selection criteria do not indicate that hyperplastic polyps are limited to the left colon; rather they were used to select the most typical and unequivocal lesions for RNA profiling.

### RNA isolation

Biopsies taken for RNA sequencing analysis were placed immediately into RNAlater® (Invitrogen) and stored at 4°C overnight prior to total RNA isolation using TRIzol (Invitrogen) the following day. Total RNA was prepared from biopsies of SSA/Ps (n = 21, 10≥1 cm diameter) plus patient matched uninvolved colon (n = 10) from SPS patients, adenomatous polyps (APs, n = 10, 5–10 mm) plus uninvolved patient matched colon (n = 10) and normal control colon (n = 10 with screening colonoscopy with no polyps) as described previously [27], [29]. The quantity of RNA recovered from samples was measured by NanoDrop analysis and only samples with a RIN of ≥7 determined by Agilent 2100 Bioanalyzer analysis were used in this study. Total RNA from twenty-one SSA/Ps and patient matched uninvolved colonic mucosa from eleven SPS patients was used for qPCR verification analysis. Total RNA (RIN of ≥7) from adenomatous polyps and uninvolved colonic mucosa from 17 patients undergoing screening colonoscopy (seven with adenomas and ten without polyps) was used for qPCR analysis (see Table S1 in File S1).

### RNA Sequencing and Bioinformatic Analysis of Gene Expression Datasets

5′ capped RNA was isolated from seven SSA/Ps and six patient matched control biopsy samples from five SPS patients and two normal screening colonoscopy specimens. The number of SSA/Ps analyzed by RNA-seq was determined by the time required to prospectively collect samples with high quality RNA and sequencing costs. PCR amplified cDNA sequencing libraries were prepared using random hexamers following the Illumina RNA sequencing protocol and single-end 50 bp RNA-seq reads (Illumina HiSeq 2000) were performed as described previously [26]–[28], [30]. Sequencing reads were aligned to the GRCh37/Hg19 human reference genome using the Novoalign application (Novocraft). Visualization tracks were prepared for each dataset using the USeqReadCoverage application and viewed using the Integrated Genome Browser (IGB) as described previously [27], [28]. Visualization tracks were scaled using reads per kilobase of gene length per million aligned reads (RPKM) for each Ensemble gene. The USeqOverdispersedRegionScanSeqs (ORSS) application was used to count the reads intersecting exons of each annotated gene and score them for differential expression in uninvolved colon and colon polyps [27], [28]. These p-values are controlled for multiple testing using the Benjamini and Hochberg false discovery rate (FDR) method as in prior studies [27], [28]. This testing correction is necessary since thousands of genes are analyzed simultaneously and there is a high likelihood for statistical false positives. The Benjamini and Hochberg procedure takes into account the expected proportion of false discoveries. A normalized ratio is also used to score and filter differentially expressed genes (FDR<0.05, 5 out of 100 false) by their enrichment (≥1.5-fold). The RNA-seq datasets described in this study have been deposited in GEO (GSE46513). Hierarchical clustering of log2 ratios (polyp/control) comparing SSA/P RNA-Seq and microarray data (APs GSE8671 and SSA/Ps GSE12514) were performed using Cluster 3.0 and Java treeview software [27], [28]. The fold change and false discovery rate of differentially expressed genes in the microarray datasets were determined using the “MULTTEST” R programming script. Gene set enrichment analysis of differentially expressed gene lists was performed using the Molecular Signatures Database (MSigDB, Broad Institute) [31]. Four tubular and three tubulovillous adenomas showing low dysplasia, part of a curated gene set available in the MSigDB, were selected for comparison to SSA/Ps. The adenomas were sex matched (4 females, 3 males), between 1.0 and 3.0 cm in diameter (1.8 mean diameter) and from right (n = 3) and left (n = 4) colon [32]. Log_2_ ratio values were determined by comparing the adenoma datasets to the uninvolved colon control datasets from the same patients.

### Real-time PCR (qPCR)

qPCR analysis of five genes, *MUC17, VSIG1, GJB5, REG4* and *ALDOB*, was done with the Roche Universal Probe Library and Lightcycler 480 system (Roche Applied Science) on control, uninvolved, SSA/P and AP colon samples [29]. cDNA was prepared from total RNA isolated from polyp and colon specimens and assayed for mRNA levels of selected genes to verify changes observed in the RNA-seq analysis. First-strand cDNA was synthesized using Moloney Murine Leukemia Virus reverse transcriptase (SuperScript III; Invitrogen) with 2 to 5 µg of RNA at 50°C (60 min) with oligo(dT) primers. Each PCR reaction was carried out in a 96-well optical plate (Roche Applied Science) in a 20 µl reaction buffer containing LightCycler 480 Probes Master Mix, 0.3 µM of each primer, 0.1 µM hydrolysis probe and approximately 50 ng of cDNA (done in triplicate). Triplicate incubations without template were used as negative controls. The qPCR thermo cycling was 95°C for 5 min, 45 cycles at 95°C for 10 sec, 60°C for 30 sec and 72°C for 1 sec. The relative quantity of each RNA transcript, in polyps compared to controls, was calculated with the comparative Ct (cycling threshold) method using the formula 2^ΔCt^. β-actin (*ACTB*) was used as a reference gene.

### BRAF Mutation Analysis

cDNA from the same “syndromic” SSA/Ps used for RNA analysis and cDNA from ten hyperplastic polyps (HPs, n = 10, 3–9 mm diameter) collected from the left colon of five SPS patients were analyzed. PCR amplicons of *BRAF* from SSA/Ps, HPs and patient matched uninvolved colon were sequenced for V600E *BRAF* mutations. Amplicons spanning exons 13–18 of the BRAF gene including the V600E mutation region were prepared (forward primer 5′-AGGGCTCCAGCTTGTATCAC-3′ and reverse primer 5′-CGATTCAAGGAGGGTTCTGA-3′, 20 ng of cDNA was amplified with 40 cycles of 95°C for 30 seconds, 53°C for 30 sec, and 72°C for 30 sec) and sequenced in both directions with a Applied Biosystems 3130 Genetic Analyzer.

### Immunohistochemistry

Representative SSA/Ps were retrospectively collected from patients with serrated polyposis syndrome and “sporadic” SSA/Ps, HPs, adenomas and uninvolved colon were collected from 55 surveillance and screening colonoscopy patients without familial risk of colon cancer seen at the University of Utah Hospital. These patients were between 46 and 78 years of age and were seen between 2009 and 2012. Each of these colonic biopsies was analyzed for VSIG1, MUC17, CTSE, TFF2 and REG4 protein expression by immunohistochemistry. Each polyp and control immunohistochemistry slide was reviewed and scored by an expert GI pathologist (MPB) in a blinded fashion. Polyclonal antigen affinity purified goat, sheep and rabbit primary antibodies were purchased from R&D Systems (anti-VSIG1, cat. #AF4818; anti-CTSE, cat #AF1294; anti-REG4, cat.#AF1379), Sigma-Aldrich (anti-MUC17, cat #HPA031634), ProteinTech (anti-TFF2, cat #12681-1-AP. Four-micron sections of formalin-fixed paraffin-embedded tissue were mounted on positively charged super-frost/plus slides. Section were deparaffinized with Neo-Clear® Xylene Substitute (Millipore cat. # 65351) and rehydrated in a graded series of alcohol to distilled water. Antigen retrieval was performed per the suppliers instructions for each antibody by heating on water bath at 95°C for 30 min. either in 10 mM citrate buffer (pH 6.0) or 10 mM Tris-EDTA Buffer (pH 9.0). Prior to incubation with primary antibodies tissue sections were incubated with a blocking solution of 2.5% normal horse serum (Vector laboratories, cat# S-2012) for 30 min at room temperature. Tissue sections were incubated for 1 hour at room temperature with optimal dilutions of each primary antibody. Samples were washed with 1× PBS (phosphate-buffered saline) and 1× PBS +1% Tween 20. Peroxidase immunostaining was performed, after treatment with BLOXALL™ (Vector Laboratories) endogenous peroxidase blocking solution, using the ImmPRESS polymer system and ImmPACT DAB substrate (Vector Laboratories) per the manufacturer's instructions. Sections were counterstain with hematoxylin QS (Vector Laboratories cat # H-3404). Controls included no primary antibody.

## Results

### Gene Expression Analysis

**Figure 2:**
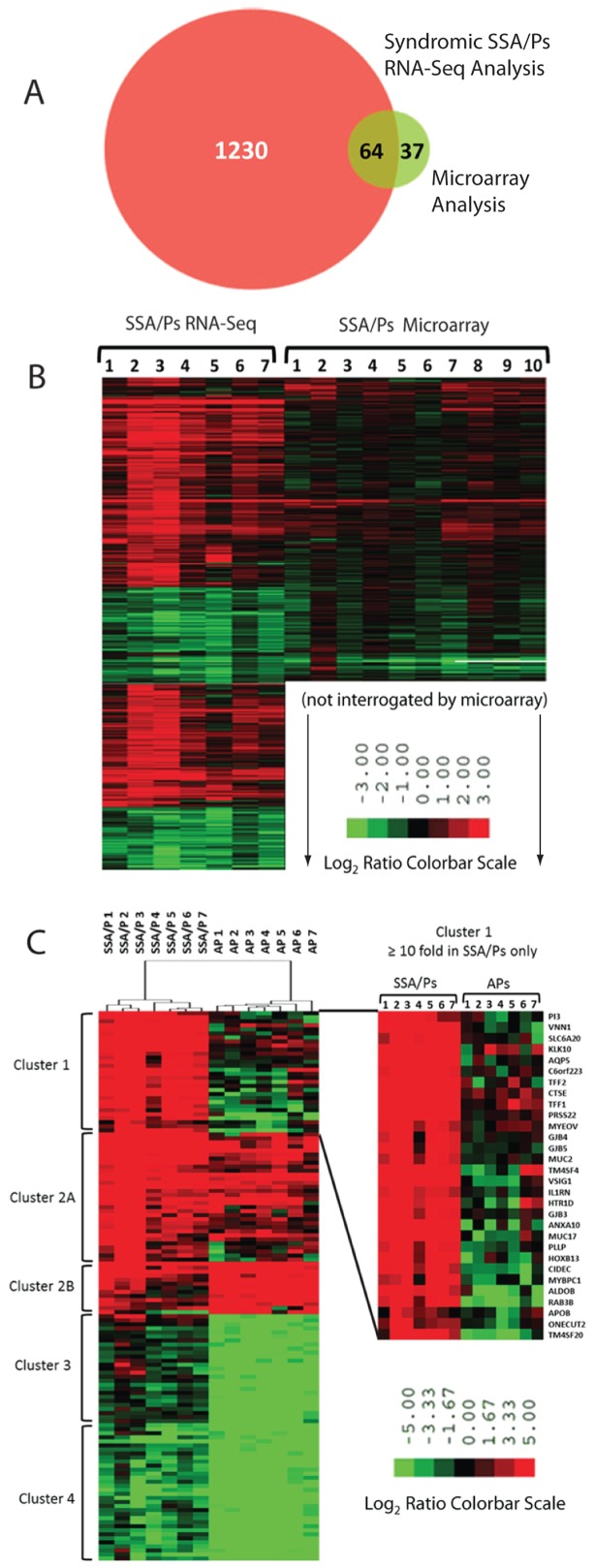
Differentially expressed genes in sessile serrated adenoma/polyps (SSA/Ps) by RNA sequencing (RNA-seq) and microarray analyses. **Panel A**. RNA-seq analysis identified 1294 genes (875 increased, 419 decreased) that were significantly differentially expressed (fold change ≥1.5, FDR<0.05) in SSA/Ps as compared to control colon biopsies. Differentially expressed genes in SSA/Ps that were found by RNA-seq analysis (red) and those found in a microarray study (green; 101 total, 59 increased, 42 decreased) are shown in the Venn diagram (23). **Panel B**. Hierarchical clustering of the differentially expressed genes in Panel A. Note: only 782 genes could be compared in the hierarchical clustering analysis because fewer genes were interrogated in the microarray analysis. **Panel C**. Hierarchical clustering of differentially expressed genes in SSA/Ps identified by RNA-seq analysis and in adenomatous polyps (APs) identified by microarray analysis (24). 136 genes (75 increased, 61 decreased) with a fold change ≥10 and FDR of <0.05 from both datasets were compared. Four distinct clusters are shown, cluster 1 represents genes increased in only SSA/Ps, cluster 2 represents genes increased in both SSA/Ps and APs, cluster 3 represents genes decreased only in APs, and cluster 4 represents genes decreased in both SSA/Ps and APs. Note: the full range of fold change is not reflected in color bar scale, the maximum fold change in RNA-seq analysis was 582-fold (*MUC5AC*) in SSA/Ps and 208-fold (*GCG*) in APs by microarray analysis.

Right-sided (cecum, ascending and transverse colon) SSA/Ps were collected from eleven patients with SPS (Table 1, Table S2 in File S1, Figure 1) and RNA isolated for RNA-seq and qPCR analysis. Seven and twenty-one SSA/Ps were used for RNA-sequencing and qPCR analysis, respectively (Table S2 in File S1). Bioinformatics analysis of the 5′ capped RNA-seq data identified 1,294 differentially expressed annotated genes [fold change ≥1.5 and false discovery rate (FDR) <0.05] in SSA/Ps as compared to patient matched uninvolved surrounding colon and normal controls (screening colonoscopy patients with no polyps) (Table S4 in File S1). At least half of the 50 most highly increased genes (all ≥14-fold, many >50-fold) and 25 most decreased genes were not identified in previous expression microarray studies of SSA/Ps (Table 2, Table S3 in File S1) [24], [25]. RNA-seq analysis identified more differentially expressed genes in SSA/Ps (1,294), by an order of magnitude, as compared to a prior microarray analysis [24] (Figure 2A). Moreover, 249 of these transcripts were changed ≥5-fold in the RNA-seq analysis as compared to only ten in the array analysis (Figure 2B). A microarray study of RNA extracted from SSA/Ps that were formalin fixed and paraffin embedded identified 71 genes that were ≥5 fold in SSA/Ps [25]. The increased number of differentially expressed genes we observed in our RNA-Seq data is consistent with the greater dynamic range of gene expression measurements in RNA-seq analysis.

**Table 2:** Top 50 gene transcripts increased by RNA sequencing in sessile serrated polyps (SSA/P) in serrated polyposis patients compared to controls.

Ensembl ID	Gene Symbol	Gene Description	SSA/P^Fold^	SSA/P^FDR^	AP^Fold^	AP^FDR^
**ENSG00000215182**	MUC5AC	Mucin 5AC, oligomeric mucus/gel-forming	582	<0.001	15	0.471
**ENSG00000129451**	KLK10	Kallikrein-related peptidase 10	378	<0.001	2.8	0.169
**ENSG00000169903**	TM4SF4	Transmembrane 4 L six family member 4	378	<0.001	2.3	0.588
**ENSG00000196188**	CTSE	Cathepsin E	116	<0.001	2.3	0.016
**ENSG00000101842**	*VSIG1	V-set and immunoglobulin domain containing 1	106	<0.001	−1.3	0.863
**ENSG00000160181**	TFF2	Trefoil factor 2	96	<0.001	1.6	0.630
**ENSG00000206075**	SERPINB5	Serpin peptidase inhibitor, clade B, member 5	92	<0.001	11	<0.001
**ENSG00000169035**	KLK7	Kallikrein-related peptidase 7	90	<0.001	2.6	0.029
**ENSG00000134193**	REG4	Regenerating islet-derived family, member 4	87	<0.001	11	<0.001
**ENSG00000169876**	MUC17	Mucin 17, cell surface associated	82	<0.001	−1.1	0.938
**ENSG00000160182**	TFF1	Trefoil factor 1	79	<0.001	2.8	0.123
**ENSG00000087916**	*SLC6A14	Solute carrier family 6, member 14	72	<0.001	3.9	0.028
**ENSG00000140279**	*DUOX2	Dual oxidase 2	70	<0.001	7.6	0.001
**ENSG00000109511**	ANXA10	Annexin A10	67	<0.001	−1.3	0.746
**ENSG00000179546**	*HTR1D	Serotonin receptor 1D	64	<0.001	1.8	0.702
**ENSG00000167757**	KLK11	Kallikrein-related peptidase 11	55	<0.001	16	<0.001
**ENSG00000140274**	*DUOXA2	Dual oxidase maturation factor 2	53	<0.001	7.3	0.004
**ENSG00000062038**	CDH3	Cadherin 3	51	<0.001	76	<0.001
**ENSG00000112299**	VNN1	Vanin 1	48	<0.001	1.4	0.609
**ENSG00000198203**	*SULT1C2	Sulfotransferase family, cytosolic, 1C, member 2	44	<0.001	5.1	0.017
**ENSG00000161798**	AQP5	Aquaporin 5	38	<0.001	1.0	0.958
**ENSG00000124102**	*PI3	Peptidase inhibitor 3, skin-derived	34	<0.001	1.0	1
**ENSG00000163347**	CLDN1	Claudin 1	32	<0.001	6.7	<0.001
**ENSG00000163993**	*S100P	S100 calcium binding protein P	30	<0.001	7.4	<0.001
**ENSG00000120875**	*DUSP4	Dual specificity phosphatase 4	30	<0.001	4.8	<0.001
**ENSG00000189280**	GJB5	Gap junction protein, beta 5	27	<0.001	−1.2	0.660
**ENSG00000163817**	*SLC6A20	Solute carrier family 6, member 20	26	<0.001	1.1	0.873
**ENSG00000137699**	*TRIM29	Tripartite motif containing 29	25	<0.001	5.8	<0.001
**ENSG00000005001**	*PRSS22	Protease, serine, 22	25	<0.001	1.4	0.308
**ENSG00000184292**	TACSTD2	Tumor-associated calcium signal transducer 2	24	<0.001	29	0.032
**ENSG00000110080**	*ST3GAL4	ST3 beta-galactoside alpha-2, 3-sialyltransferase 4	23	<0.001	2.5	0.093
**ENSG00000170786**	SDR16C5	Short chain dehydrogenase/reductase family 16C5	22	<0.001	3.8	0.007
**ENSG00000136872**	*ALDOB	Aldolase B	20	<0.001	−2.0	0.703
**ENSG00000159184**	*HOXB13	Homeobox B13	19	<0.001	−1.2	0.895
**ENSG00000135480**	KRT7	Keratin 7	19	<0.001	−1.1	0.907
**ENSG00000189433**	*GJB4	Gap junction protein, beta 4	18	<0.001	1.1	0.780
**ENSG00000084674**	*APOB	Apolipoprotein B	18	<0.001	1.0	0.988
**ENSG00000167653**	*PSCA	Prostate stem cell antigen	18	<0.001	−1.4	0.848
**ENSG00000187288**	*CIDEC	Cell death-inducing DFFA-like effector c	18	<0.001	−2.2	0.31
**ENSG00000221947**	*XKR9	XK, Kell blood group complex subunit family member 9	17	<0.001	na	na
**ENSG00000168631**	*DPCR1	Diffuse panbronchiolitis critical region 1	16	<0.001	1.4	0.728
**ENSG00000169213**	*RAB3B	RAB3B, member RAS oncogene family	16	<0.001	−4.5	<0.001
**ENSG00000130720**	FIBCD1	Fibrinogen C domain containing 1	16	<0.001	1.0	1
**ENSG00000147206**	NXF3	Nuclear RNA export factor 3	16	<0.001	6.5	0.355
**ENSG00000162366**	*PDZK1IP1	PDZK1 interacting protein 1	15	<0.001	2.5	<0.001
**ENSG00000139800**	ZIC5	Zic family member 5	15	<0.001	1.4	0.762
**ENSG00000213822**	*CEACAM18	Carcinoembryonic antigen cell adhesion molecule 18	15	<0.001	na	na
**ENSG00000163739**	*CXCL1	Chemokine (C-X-C motif) ligand 1	15	<0.001	7.2	<0.001
**ENSG00000112559**	*MDFI	MyoD family inhibitor	14	<0.001	2.1	0.002
**ENSG00000119547**	ONECUT2	One cut homeobox 2	14	<0.001	−1.3	0.684

Fold change is reported for seven right-sided sessile serrated polyps, from five serrated polyposis patients (age 26–62 years, 3 female and 2 male), compared to surrounding uninvolved colon and normal colon from healthy volunteers (controls, n = 8). Fold-change (Fold) and false discovery rate (FDR) for specific gene sequencing reads are provided (see Methods). The fold change and FDR in sex matched adenomatous polyps (AP) (age 55–79 years, three right-sided and four left-sided) with low dysplasia compared to uninvolved colon (n = 7) from a previous microarray study are provided (Sabates-Bellver, et al., 2007). Genes with an asterisk have not been previously reported to be differentially expressed in SSA/Ps. “na” denotes transcripts not analyzed in the microarray study.

**Figure 3:**
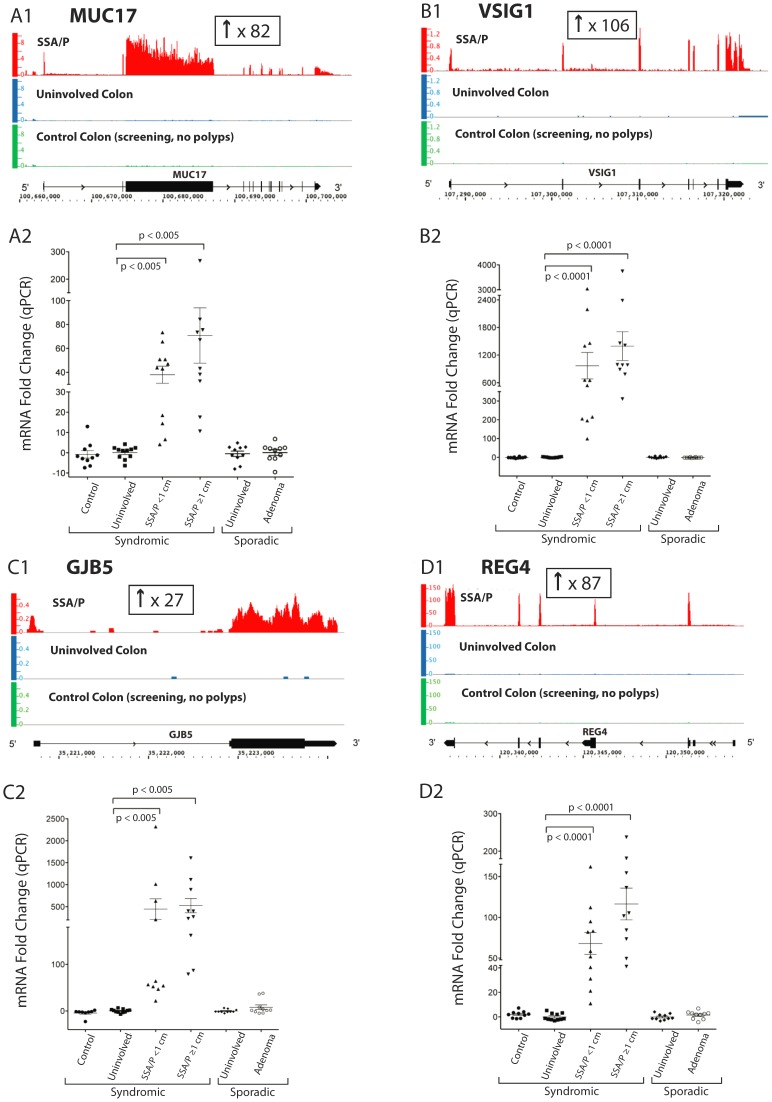
Expression of mucin 17 (*MUC17*), V-set and immunoglobulin domain containing 1 (*VSIG1*), gap junction protein, beta 5 (*GJB5*) and regenerating islet-derived family member 4 (*REG4*) in SSA/Ps, adenomatous polyps (APs) and controls as measured by RNA-seq analysis. **Panel A1**. *MUC17* RNA-seq results. The y-axis represents the number of uniquely mapped sequencing reads per kilobase of transcript length per million total reads (RPKM) mapped to the *MUC17* locus. The x-axis represents the chromosome (Chr) 7 coordinates and gene structure of the *MUC17* transcript. Analysis showed an 82-fold increase in *MUC17* mRNA in SSA/Ps (red, n = 7 polyps) compared to uninvolved colon (patient matched uninvolved, blue, n = 6) and control colon (screening colon without polyps; green, n = 2). The sequencing read length was 50 base pairs. **Panel A2**. *MUC17* expression measured by qPCR analysis in SSA/Ps, adenomatous polyps and controls in additional patients. Relative mRNA levels of *MUC17* in large (>1 cm) and small (<1 cm) SSA/Ps (n = 21), adenomatous polyps (n = 10), uninvolved colon and normal control colon biopsies (n = 10 each) are shown. In small and large SSA/Ps, *MUC17* expression was increased by 38 and 71-fold, respectively, compared to controls. qPCR results were normalized to β-actin. The average *MUC17* expression level in uninvolved colon tissue was chosen as the baseline. P-values were calculated using the Mann-Whitney U-test. **Panel B1**. *VSIG1* (Chr X) RNA-seq results. A 106-fold increase in expression of *VSIG1* was found in SSA/Ps as compared to controls. **Panel B2**. *VSIG1* qPCR results. In small and large SSA/Ps, *VSIG1* expression was increased 969 and 1393-fold, respectively. **Panel C1**. *GJB5* (Chr 1) RNA-seq results. A 27-fold increase in *GJB5* mRNA was found in SSA/Ps. **Panel C2**. *GJB5* qPCR results. In small and large SSA/Ps, *GJB5* expression was increased 446 and 523-fold, respectively. **Panel D1**. *REG4* (Chr 1) RNA-seq results. An 87-fold increase in *REG4* mRNA was found in SSA/Ps. **Panel D2**. *REG4* qPCR results. In small and large SSA/Ps, *REG4* mRNA was increased 68 and 116-fold, respectively.

Differentially expressed genes in the RNA-seq SSA/Ps dataset were compared to adenomatous polyp data that is part of a curated gene set available in the Molecular Signature Database at the Broad Institute [31], [32]. Differentially expressed genes from an equal number of adenomatous polyps from sex matched patients (n = 7, three men & four women) with low dysplasia were used for comparison. To identify genes that were highly expressed in SSA/Ps, but not in adenomatous polyps, we did hierarchical clustering analysis of 142 differentially expressed genes (>10-fold, FDR<0.05) from each dataset (Figure 2C). Fold changes were determined by comparing SSA/Ps and adenomas to their corresponding uninvolved control colon specimen datasets from each study. Approximately 60% of the 75 most highly differentially expressed genes in SSA/Ps (50 increased and 25 decreased) were not differentially expressed in adenomatous polyps relative to controls (Table 2, Table S3 in File S1). Genes that were highly increased (≥10-fold, 30 genes) in SSA/Ps (Figure 2C), but not significantly increased in adenomatous polyps, were analyzed by gene set enrichment (GSEA) analyses. Three biological pathways overrepresented in SSA/Ps were mucosal integrity (digestion), cell communication (adhesion) and epithelial cell development. Secreted trefoil factor and mucin genes associated with mucosal integrity that were increased included, mucin 5AC (*MUC5AC*,↑582-fold), cathepsin E (*CTSE*, ↑116-fold), trefoil factor 2 (*TFF2*, ↑96-fold), trefoil factor 1 (*TFF1*, ↑79-fold) and mucin 2 (*MUC2*, ↑14-fold) (Table 2, Table S4 in File S1). A membrane bound regulatory mucin, Mucin 17 (*MUC17*, ↑82-fold), was also highly increased in SSA/Ps (Figure 3A1).

RT-qPCR verification analysis of genes that were markedly increased in SSA/Ps by RNA-seq were done with twenty-one right sided SSA/Ps and uninvolved colon from SPS patients, ten right sided adenomatous polyps plus uninvolved colon and ten right sided normal control biopsies. The genes were selected based on their high levels of expression and optimal primer design. qPCR analysis verified the marked overexpression of *MUC17* (38-fold in small; 71-fold in large SSA/Ps) in SSA/Ps compared to adenomatous polyps and controls (Figure 3A2). The gene for a cell adhesion protein, membrane associated V-set and immunoglobulin domain containing 1 gene (*VSIG1*), that was markedly increased by RNA-seq analysis (↑106-fold) was also highly increased in SSA/Ps by qPCR analysis (969-fold in small; 1,393-fold in large SSA/Ps) (Figure 3B). Expression of several gap junction (connexin) genes were also highly increased in SSA/Ps including gap junction protein beta-5 (*GJB5* or connexin 31.1, ↑27-fold), gap junction protein, beta 3 (GJB3 or connexin 31, ↑14-fold) and gap junction protein beta 4 (GJB4 or connexin 30.3, ↑18-fold) (Figure 3C; Table 2, Table S4 in File S1). qPCR analysis verified the increase in *GJB5* in SSA/Ps (446 and 523-fold in small and large polyps, respectively) relative to adenomatous polyps and controls (Figure 3C2). Three tetraspanin genes, encoding proteins that interact with cell adhesion molecules and growth factor receptors, transmembrane 4 L six family member 4 (*TM4SF4*, ↑378-fold), transmembrane 4 L six family member 20 (*TM4SF20*, ↑14-fold) and plasmolipin (*PLLP*, ↑11-fold) were highly increased in SSA/Ps.

Other highly expressed genes in SSA/Ps, reported to be increased in inflammatory or neoplastic conditions of the colon, included regenerating islet-derived family member 4 (*REG4*, ↑87-fold; Figure 3D1), kallikrein 10 (*KLK10*, ↑378-fold), aquaporin 5 (*AQP5*, ↑38-fold), myeloma overexpressed (*MYEOV*, ↑14-fold) and aldolase B (*ALDOB* or fructose-bisphosphate aldolase B, ↑20-fold) (Figure S1 in File S1,Table 2, Table S4 in File S1). qPCR analysis confirmed the increase in *ALDOB* (33 to 38-fold) in SSA/Ps (Figure S1 in File S1). Increased expression of *REG4* was reported in gastric intestinal metaplasia and colonic adenomatous polyps suggesting a role in premalignant lesions [33]. qPCR analysis verified the increase in *REG4* (68 to 116-fold) in SSA/Ps compared to controls (Figure 3D2). The transcription factors homeobox B13 (*HOXB13*, ↑19-fold) and one cut homeobox 2 (*ONECUT2*, ↑14-fold), critical in epithelial cell development and differentiation, both had >10-fold increases in their mRNA in SSA/Ps by RNA-seq analysis (Table 2). Neither of these transcription factors was significantly expressed in control specimens (0.006–0.03 RPKM).

### BRAF Mutation Analysis

*BRAF* cDNA from syndromic SSA/Ps, hyperplastic polyps (HPs) and normal colon was amplified by PCR and sequenced since T to A mutations in codon 600 resulting in a valine to glutamic acid (V600E) amino acid change with increased kinase activity have been reported in SSA/Ps (Materials and Methods) [14]. PCR amplicons from twenty SSA/Ps, ten HPs, and patient matched uninvolved control specimens were sequenced. We found that 60% of SSA/Ps had V600E mutations in *BRAF* while no mutations were observed in hyperplastic polyps and controls (Table S5 in File S1) [14], [15]. It should be noted that some studies have found *BRAF* mutations in approximately 33% of HPs [34].

### Immunohistochemistry

Immunohistochemistry (IHC) for VSIG1, MUC17, CTSE, TFF2 and REG4 in a panel of routinely formalin fixed and paraffin embedded SSA/Ps, hyperplastic polyps, adenomatous polyps, and control specimens was done to further validate the RNA-seq data, identify the cell types involved in overexpression and to investigate their potential diagnostic utility for differentiating SSA/Ps from other polyps. All control and polyp specimens were reviewed by an expert GI pathologist (MPB).

**Figure 4:**
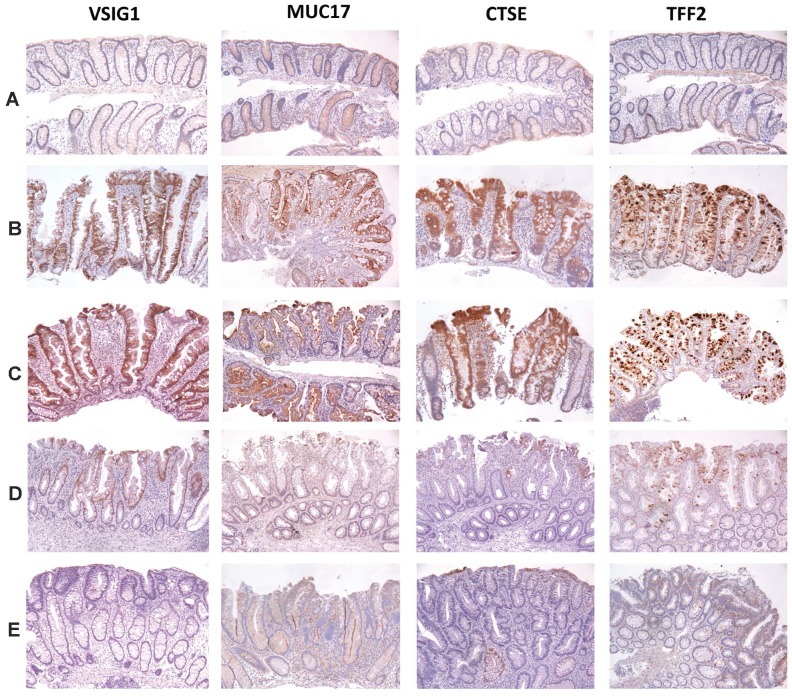
Immunostaining for VSIG1, MUC17, CTSE and TFF2 in control colon, SSA/Ps, hyperplastic and adenomatous polyps. Representative images of immunoperoxidase staining with affinity purified polyclonal antibodies and formalin-fixed, paraffin-embedded biopsies of patient matched and normal control colon (Panel A, n = 15, see Methods), syndromic SSA/Ps (Panel B, n = 10), sporadic SSA/Ps (Panel C, n = 15), hyperplastic polyps (Panel D, n = 10) and adenomatous polyps (Panel E, n = 10) are shown. Representative immunohistochemical stains for REG4 in control and polyp specimens are provided in Figure S2 in File S1.

We found intense and unique patterns of staining for VSIG1, MUC17, CTSE and TFF2 that differentiated SSA/Ps from other polyps and controls (Figure 4, Table 3). Immunostaining for VSIG1 was absent in control colon (Figure 4A), whereas with both syndromic (Figure 4B) and sporadic SSA/Ps (Figure 4C) there was intense (3 to 4+, on a scale of 0–4, 4 being highest) staining of most epithelial cell junctions (>70%) in both the luminal surface and along the crypt axis (Figure 4, Table 3, Figure S2F in File S1). Hyperplastic polyps (Figure 4D) showed trace to 1+ immunostaining in ∼25% of epithelial cells. Adenomatous polyps (Figure 4E) showed trace or no staining. Immunostaining for MUC17 in the cytoplasm of control colon epithelium was trace, whereas with SSA/Ps there was a distinctive pattern of staining that was 2 to 3+ in the cytoplasm of approximately 60% of epithelial cells and most pronounced at the luminal surface, but which progressively decreased toward the crypt bases (Figure 4, Table 3). Hyperplastic polyps showed trace to 1+ staining in <10% of luminal epithelial cells. Adenomatous polyps showed only trace diffuse immunostaining. Immunostaining for CTSE was only trace in the cytoplasm of surface epithelial cells in control colon, whereas with both syndromic and sporadic SSA/Ps there was 3 to 4+ staining of the cytoplasm in approximately 75% of epithelial cells that was often more pronounced at the luminal surface but also extended along the crypt axis (Figure 4, Table 3). Hyperplastic polyps showed only trace to 1+ immunostaining in <25% of epithelial cells. Adenomatous polyps showed only trace staining in rare glands. Immunostaining for TFF2 showed trace to no staining in control colon luminal epithelial cells, whereas SSA/Ps showed 3 to 4+ staining of goblet cell mucin in >60% of both surface and crypt cells (Figure 4, Table 3). Hyperplastic polyps also showed 2 to 3+ immunostaining of goblet cell mucin in >60% of surface and crypt cells. Adenomatous polyps showed only trace staining in <10% of luminal epithelial cells.

**Table 3:** Immunohistochemical analysis of different serrated and adenomatous polyp types for proteins encoded by genes found to be highly differentially expressed in SSA/Ps.

Polyp Type	VSIG1		MUC17		CTSE		TFF2	
	IHC* positive	Mean score* (0–4)	IHC positive	Mean score (0–4)	IHC positive	Mean score (0–4)	IHC positive	Mean score (0–4)
**Sessile serrated adenoma/polyp, syndromic**	11/11*	3.4	12/12	2.0	11/11	3.3	10/10	3.9
**Sessile serrated adenoma/polyp, sporadic**	23/23	3.1	17/17	2.9	15/15	2.6	15/15	3.7
**Hyperplastic polyp**	5/10	1.4	3/10	0.6	3/11	1.2	11/11	2.9
**Adenomatous polyp**	1/13	0.2	3/13	0.2	1/12	0.2	2/12	0.3
**Uninvolved colon mucosa**	0/8	0	0/5	0	0/5	0	0/4	0
**Normal colon mucosa**	0/16	0	0/11	0	0/10	0	0/13	0

* The number of polyp or normal colonic specimens that showed positive immunohistochemical staining (IHC) over the total number of independent samples examined are shown. The intensity of IHC staining was scored 0 (none) to 4 (maximal). See Pathologic Classification in Methods for the criteria used to select polyps for study.

In contrast to the other proteins, intense immunostaining for REG4 was found in SSA/Ps, hyperplastic polyps and adenomatous polyps and weak to intermediate staining in control colon (Figure S2 in File S1). Specifically, there was 1 to 2+ staining for REG4 in control colonocyte cytoplasm and staining in approximately 50% of goblet cells, whereas with SSA/Ps there was 4+ staining of the full mucosal thickness including 4+ staining of >90% of goblet cells. Hyperplastic polyps also showed 3 to 4+ in >75% of epithelial cells with little staining at the crypt bases. Adenomatous polyps also showed 2 to 3+ immunostaining and in a different (more diffuse pattern) than SSA/Ps or hyperplastic polyps.

## Discussion

The RNA-seq transcriptome analyses we report identifies many highly and differentially expressed genes in SSA/Ps relative to adenomatous polyps and provide detailed support for the hypothesis that the progression of SSA/Ps and adenomatous polyps to colon cancer are different. Mucin and trefoil factor related genes, that have variety of functions related to oncogenesis, were among the highly increased genes. *MUC2*, *MUC5A* and *MUC6*, are part of a gel-forming group of mucins that have epithelial barrier functions, provide a habitat for gut microbes and also contain EGF-like motifs (e.g., MUC17) predicted to regulate cell growth [35]. Although a previous study indicated that *MUC6* is over expressed in SSA/Ps, our RNA-seq analysis identified a number of other mucin genes with markedly increased expression in SSA/Ps compared to controls [35], [36]. For example, mucin 5AC (*MUC5AC*) mRNA was increased 582-fold in SSA/Ps by RNA-seq and has been reported to have increased expression in colon cancers [35]. Unfortunately we did find suitable antibodies to test immunostaining of mucin 5AC in SSA/Ps and other polyps. Interestingly, mucin 2 (*MUC2*) mRNA that was increased 14–fold in SSA/Ps by RNA-seq has been reported to be decreased in colon cancers arising from adenomatous polyps [37]. Increased MUC2 expression stimulates metastasis and decreased expression reduces metastasis of colon cancer cells [38]. Another new finding was that mucin 17 (*MUC17*) was markedly increased (82-fold) in SSA/Ps but not adenomatous polyps. Mucin 17 is a membrane bound mucin with EGF repeats that maintains epithelial barrier function, plus *MUC17* has NFkB and homeobox transcription factor CDX-2 response elements [39], [40]. The regulation of *MUC17* by NFkB suggests that inflammation and microbiota might be relevant to SSA/P development and biology. We also identified a marked increase in trefoil factor 2 (↑96-fold) and trefoil factor 1 (↑79-fold) that have been reported to not be increased in adenomatous polyps. mRNA levels for *TFF1* and *TFF2* were increased in SSA/Ps in a previously reported microarray analysis [24], [25]. Increased expression of trefoil factors contribute to mucosal repair and cell migration and increased levels of TFF2 have been reported in gastric cancer and correlate with reduced survival [41].

Several cell adhesion and tetraspanin family genes showed markedly increased expression in SSA/Ps by RNA-seq analysis and have not been previously reported to be increased in any type of colon polyp. This included V-set and immunoglobulin domain containing 1 gene (*VSIG1*), a member of the junctional adhesion (JAM) family of proteins, that regulate tight junctions and mediate other critical epithelial cell functions [42]. *VSIG1*, also known as glycoprotein 34 (GPA34) and normally only significantly expressed in stomach and testis, is overexpressed in gastric, esophageal and ovarian cancers but reported not to be increased in colon cancers [43]. *VSIG1* has been considered critical in maintaining differentiation of glandular gastric epithelium [44]. In addition, the GPA33 glycoprotein which is similar to VSIG1 is markedly increased in most colon cancers and has been used as a target for therapeutics [45]. Among the many novel highly upregulated genes found in SSA/Ps were gap junction (*GJB3*, *4*, and *5* were increased >10-fold) and transmembrane 4 proteins which are members of the tetraspanin transmembrane protein gene family that regulate cell adhesion, motility, proliferation and metastasis [46]. Gap junction proteins promote stromal-epithelial interactions in tumors, have a role in tumor progression and *GJB3* and *GJB5* regulate survival of some stem cells [47]. Tetraspanins, of which mRNA for *TM4SF4*, *TM4SF20* and *PLLP* were increased >10-fold in SSA/Ps, regulate cell adhesion and proliferation by binding integrins and growth factor receptors, are increased in multiple types of cancer and regulate epithelial-to-mesenchymal transition [46]. Levels of *TM4SF4* mRNA were reported to be increased in SSA/Ps in a microarray study of formalin fixed paraffin embedded specimens [25].

We report many previously unrecognized differentially expressed genes in SSA/Ps that should prove useful in determining the pathways leading to their development and progression to colon cancer. Among the markedly increased genes in SSA/Ps that have also been reported to be increased in colon cancer were *REG4, AQP5, MUC2, TFF1, KLK10*, [24], [33], [35], [48], [49]. Our mining of a microarray dataset of serrated colon carcinoma specimens determined that peptidase inhibitor 3 (*PI3*), vanin 1 (*VNN1*) and annexin 10 (*ANXA10*) are more highly expressed in such cancers as compared to conventional colon carcinomas [50]. *ANXA10* was recently suggested as an immunohistochemical marker of SSA/Ps [25]. *VNN1*, increased 49-fold in SSA/Ps by RNA-seq analysis, is an oxidative stress sensor in epithelial cells and plays an important role in mediating inflammatory signals during inflammation-driven carcinogenesis in animal models of colitis-associated colon cancer [51]. The *VNN1* finding, together with increased *REG4* and *MUC17*, that are also increased during colitis, suggests that the development of SSA/Ps may at least in part involve inflammatory processes [39], [52].

The marked increase in *REG4* mRNA (regenerating islet-derived family member 4, 87-fold) in SSA/Ps, is of interest because it is increased in gastric and colorectal cancers and is associated with resistance of cancer cells to chemotherapy and radiation induced cell death [33], [53], [54]. Moreover, *REG4* mRNA is increased (6 to 11-fold) in large adenomatous polyps [24]. REG4 protein also transactivates EGFR signaling and reduces apoptosis in colon cancer cells [55]. *REG4* transcription is induced by the GLI1 transcription factor, which is activated by sonic hedgehog, regulates stem cell proliferation, and is associated with a poor cancer prognosis [56]. Sonic hedgehog (*SHH*) mRNA was increased 4-fold in SSA/Ps in our analysis, consistent with increased hedgehog signaling. It seems likely that increased expression of *REG4*, and upstream factors such as hedgehog, plays a role in the biology of SSA/Ps and possibly their progression to cancer. Upregulation of the HOX transcription factors *HOXB13* (19-fold) and *ONECUT2* (14-fold) in SSA/Ps suggests altered cellular differentiation of epithelial cells in SSA/Ps. Increased expression of HOX transcription factors might be critical in the development and progression of SSA/Ps, have been shown to be increased in a variety of other cancers and to be altered by epigenetic regulatory mechanisms, including lincRNAs [57].

During the preparation of our report a gene expression study of sporadic SSA/Ps, using formalin fixed paraffin embedded (FFPE) biopsy specimens and gene arrays, was published [25]. This study compared mRNA expression of six right-sided SSA/Ps and six left-sided serrated polyps designated microvesicular hyperplastic polyps with three unmatched right and left controls, respectively. A similar number of differentially expressed genes were observed in both SSA/Ps and the microvesicular hyperplastic polyps (∼300 genes) despite suggestions that they differ in cancer risk. This result is consistent with a recent study showing that SSA/Ps and the so called microvesicular subtype of hyperplastic polyp have overlapping histological and molecular features [58]. These findings point out a potential confounding issue in studies aimed at identifying unique gene expression signatures of SSA/Ps and the rationale for not using the microvesicular hyperplastic polyp classification and why we focused on relatively large “right sided” SSA/Ps in our study. It should be noted that although Annexin A10 has been reported as a marker of SSA/Ps by qPCR and immunohistochemical analysis, one study reported significant differences in staining in SSA/Ps from left and right colon and suggested that anatomic location was a possible predictor of progression to cancer [6], [58]. Our RNA-seq transcriptome analysis of SSA/Ps identified over twelve other genes that were more highly upregulated than Annexin 10 (67-fold) in SSA/Ps, as compared to patient matched controls, and identified additional candidate immunohistochemical markers differentiating SSA/Ps from HPs and APs.

The RNA-seq gene expression and immunohistochemistry analysis we report provides evidence that molecular pathways involved in colonic mucosal integrity, cell adhesion and cell development are altered in SSA/Ps and that many of the specific gene changes altered in these pathways are not found in adenomatous polyps. However, additional studies will need to be done, including a larger sample size, studying age matched patients since our SPS cohort was on average younger in age than the screening colonoscopy patients, analyzing SSA/Ps from the left colon and serrated polyps with overlapping phenotypes. Nevertheless, our prospective collection and analysis of SSA/Ps from patients with the serrated polyposis syndrome identified differentially expressed genes that seem likely to be involved in the development and progression of SSA/Ps and may provide new strategies for preventing and treating SSA/Ps and their associated colon cancers. Although progress has been made in preventing adenomatous polyps and their progression to cancer, preclinical mechanistic evaluations of chemoprevention agents for SSA/Ps and biomarkers are likely to be required before clinical trials are instituted [59]–[62] The immunohistochemistry staining patterns that differentiated syndromic and sporadic SSA/Ps from hyperplastic and adenomatous polyps in our study, may enable the more accurate diagnosis of patients with SSA/Ps to better inform cancer screening and future clinical studies of SSA/Ps.

## Supporting Information

File S1 **This file contains Tables S1, S2, S3, S4, and S5 and Figures S1 and S2.**
(DOCX)
