# Supplementary material for: RNA Sequencing of Sessile Serrated Colon Polyps Identifies Differentially Expressed Genes and Immunohistochemical Markers
Source: PLoS One. 2014 Feb 12;9(2):e88367. doi: 10.1371/journal.pone.0088367 (PMC3922809; doi:10.1371/journal.pone.0088367)
Supplement: File S1 — This file contains Tables S1, S2, S3, S4, and S5 and Figures S1 and S2. (DOCX) [file pone.0088367.s001.docx]

**Table S1. Demographics of Patients without SPS and Controls**

**Adenomatous Polyps**

| **No of Patient** | **Age** | **Sex** |
| --- | --- | --- |
| 1 | 63 | M |
| 2 | 54 | F |
| 3 | 46 | F |
| 4 | 50 | F |
| 5 | 50 | M |
| 6 | 68 | M |
| 7 | 61 | F |
| 8 | 48 | M |
| 9 | 58 | M |
| 10 | 50 | M |

**Controls (screening colonoscopy, no polyps**

| No of Patient | Age | Sex |
| --- | --- | --- |
| 1 | 80 | M |
| 2 | 66 | M |
| 2 | 66 | M |
| 2 | 66 | M |
| 3 | 44 | M |
| 3 | 44 | M |
| 4 | 53 | F |
| 5 | 64 | M |
| 6 | 53 | F |
| 7 | 50 | M |

**Table S2. Phenotype of SSA/Ps from patients with serrated polyposis syndrome (SPS) that were analyzed by RNA-Seq and qPCR.** AC = Ascending colon; TC = Transverse Colon

| **Patient** | **Sample** | **Size Diameter (mm)** | **Location** | **Pathology** | **RNA‐Seq** | **qPCR** |
| --- | --- | --- | --- | --- | --- | --- |
| 1 | 1A | 10 | AC | SSA/P | Yes | Yes |
| 1 | 1B | 10 | TC | SSA/P | No | Yes |
| 2 | 2A | 6 | AC | SSA/P | No | Yes |
| 2 | 2B | 4 | TC | No | No | Yes |
| 3 | 3A | 8 | AC | SSA/P | Yes | Yes |
| 3 | 3B | 12 | AC | SSA/P | Yes | Yes |
| 4 | 4 | 15 | AC | SSA/P | Yes | Yes |
| 5 | 5A | 4 | AC | No | Yes | Yes |
| 5 | 5B | 5 | AC | No | No | Yes |
| 6 | 6A | 4 | AC | SSA/P | Yes | Yes |
| 6 | 6B | 4 | TC | No | No | Yes |
| 6 | 6C | 3 | AC | No | Yes | Yes |
| 7 | 7A | 12 | AC | SSA/P | No | Yes |
| 7 | 7B | 15 | TC | SSA/P | No | Yes |
| 8 | 8A | 8 | Cecum | SSA/P | No | Yes |
| 8 | 8B | 12 | AC | SSA/P | No | Yes |
| 9 | 9A | 5 | Cecum | SSA/P | No | Yes |
| 9 | 9B | 15 | AC | SSA/P | No | Yes |
| 9 | 9C | 6 | TC | SSA/P | No | Yes |
| 10 | 10 | 10 | TC | SSA/P | No | Yes |
| 11 | 11 | 12 | AC | SSA/P | No | Yes |


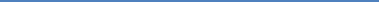
Table S3. Top 25 gene transcripts decreased by RNA sequencing in sessile serrated polyps (SSA/P) in serrated polyposis patients compared to controls. Fold change is reported for seven right-sided sessile serrated polyps, from five serrated polyposis patients (age 26-62 years, three female and two male), compared to surrounding uninvolved colon and normal colon from healthy volunteers controls, (n=8). Fold-change (Fold) and false discovery rate (FDR) are shown. The fold change and FDR in sex matched adenomatous polyps (AP) (age 55-79 years, five right-sided and two left-sided) with low dysplasia compared to uninvolved colon (n=7) from a previous microarray study (Sabates-Bellver, et al., 2007; PMID 18171984). Genes with an asterisk have not been previously reported to be differentially expressed in SSA/Ps. “na” denotes transcripts not analyzed in the microarray study.

|  | **Ensembl ID** |  | **Gene Symbol** |  | **Gene Description** |  | **SSA/PFold** |  | **SSA/PFDR** |  |  |  | **APFold** |  | **APFDR** |
| --- | --- | --- | --- | --- | --- | --- | --- | --- | --- | --- | --- | --- | --- | --- | --- |
| ENSG00000132874 SLC14A2 Solute carrier family 14, member 2 ‐19 <0.001 ‐1.2 0.908 | | | | | | | | | | | | | | | |
| ENSG00000134955 *SLC37A2 Solute carrier family 37, member 2 ‐13 <0.001 ‐4.7 0.329 | | | | | | | | | | | | | | | |
| ENSG00000183844 *FAM3B Family with sequence similarity 3, member B ‐8.2 <0.001 ‐5.0 0.647 | | | | | | | | | | | | | | | |
| ENSG00000169903 *B4GALNT2 Beta‐1, 4‐N‐acetyl‐galactosaminyl transferase 2 ‐7.7 <0.001 ‐2.2 0.205 | | | | | | | | | | | | | | | |
| ENSG00000132429 *POPDC3 Popeye domain containing 3 ‐6.3 <0.001 ‐3.4 0.403 | | | | | | | | | | | | | | | |
| ENSG00000196660 *SLC30A10 Solute carrier family 30, member 10 ‐6.1 <0.001 ‐29 <0.001 | | | | | | | | | | | | | | | |
| ENSG00000197991 *PCDH20 Protocadherin 20 ‐5.8 <0.001 ‐2.3 0.037 | | | | | | | | | | | | | | | |
| ENSG00000135220 UGT2A3 UDP glucuronosyltransferase 2 family, polypeptide A3 ‐5.6 <0.001 ‐3.9 0.004 | | | | | | | | | | | | | | | |
| ENSG00000203859 *HSD3B2 Hydroxy‐delta‐5‐steroid dehydrogenase, 3B2 ‐5.3 <0.001 ‐77 0.238 | | | | | | | | | | | | | | | |
| ENSG00000122756 CNTFR Ciliary neurotrophic factor receptor ‐4.7 <0.001 ‐1.1 0.81 | | | | | | | | | | | | | | | |
| ENSG00000064655 *EYA2 Eyes absent homolog 2 ‐4.7 <0.001 ‐6.0 <0.001 | | | | | | | | | | | | | | | |
| ENSG00000164093 *PITX2 Paired‐like homeodomain 2 ‐4.6 <0.001 ‐1.1 0.95 | | | | | | | | | | | | | | | |
| ENSG00000131482 *G6PC Glucose‐6‐phosphatase, catalytic subunit ‐4.5 <0.001 ‐3.5 0.105 | | | | | | | | | | | | | | | |
| ENSG00000204936 CD177 CD177 molecule ‐4.5 <0.001 ‐20 <0.001 | | | | | | | | | | | | | | | |
| ENSG00000244474 *UGT1A4 UDP glucuronosyltransferase 1 family, polypeptide A4 ‐4.5 <0.001 ‐1.4 0.35 | | | | | | | | | | | | | | | |
| ENSG00000138669 PRKG2 Protein kinase, cGMP‐dependent, type II ‐4.4 <0.001 ‐2.0 0.120 | | | | | | | | | | | | | | | |
| ENSG00000248144 ADH1C Alcohol dehydrogenase 1C, gamma polypeptide ‐4.2 <0.001 ‐2.8 <0.001 | | | | | | | | | | | | | | | |
| ENSG00000174992 ZG16 Zymogen granule protein 16 homolog ‐4.2 <0.001 ‐5.7 <0.001 | | | | | | | | | | | | | | | |
| ENSG00000109182 *CWH43 Cell wall biogenesis 43 C‐terminal homolog ‐4.2 <0.001 ‐3.5 <0.001 | | | | | | | | | | | | | | | |
| ENSG00000179520 *SLC17A8 Solute carrier family 17, member 8 ‐4.1 <0.001 ‐2.5 0.229 | | | | | | | | | | | | | | | |
| ENSG00000103375 AQP8 Aquaporin 8 ‐4.1 <0.001 ‐76 <0.001 | | | | | | | | | | | | | | | |
| ENSG00000124615 *MOCS1 Molybdenum cofactor synthesis 1 ‐4.0 <0.001 ‐5.2 <0.001 | | | | | | | | | | | | | | | |
| ENSG00000164128 *NPY1R Neuropeptide Y receptor Y1 ‐3.9 <0.001 ‐2.1 0.034 | | | | | | | | | | | | | | | |
| ENSG00000100505 *TRIM9 Tripartite motif containing 9 ‐3.9 <0.001 ‐5.4 0.062 | | | | | | | | | | | | | | | |
| ENSG00000182271 *TMIGD1 Transmembrane and immunoglobulin domain containing 1 ‐3.7 <0.001 na na | | | | | | | | | | | | | | | |

**Table S4. Significantly changed genes in SSA/Ps (fold change ≥ 1.5 and FDR <0.05) relative to controls.**

| **Ensembl_ID** | **Description** | **Gene_Symbol** | **BH_FDR** | **Fold** | **SSA/P_RPKM** | **Control_RPKM** |
| --- | --- | --- | --- | --- | --- | --- |
| ENSG00000215182 | mucin 5AC, oligomeric mucus/gel-forming | MUC5AC | 2.3724E-100 | 581.63 | 24.22 | 0.02 |
| ENSG00000129451 | kallikrein-related peptidase 10 | KLK10 | 3.38353E-84 | 378.45 | 23.25 | 0.03 |
| ENSG00000169903 | transmembrane 4 L six family member 4 | TM4SF4 | 1.08026E-83 | 378.14 | 39.45 | 0.06 |
| ENSG00000196188 | cathepsin E | CTSE | 3.7066E-67 | 116.15 | 141.39 | 1.72 |
| ENSG00000101842 | V-set and immunoglobulin domain containing 1 | VSIG1 | 8.91867E-65 | 106.19 | 12.35 | 0.07 |
| ENSG00000160181 | trefoil factor 2 | TFF2 | 3.01849E-50 | 95.61 | 30.98 | 0.27 |
| ENSG00000206075 | serpin peptidase inhibitor, clade B (ovalbumin), member 5 | SERPINB5 | 2.09406E-65 | 92.11 | 18.33 | 0.15 |
| ENSG00000169035 | kallikrein-related peptidase 7 | KLK7 | 1.91756E-49 | 90.54 | 6.88 | 0.00 |
| ENSG00000124233 | semenogelin I | SEMG1 | 2.43131E-42 | 88.57 | 7.78 | 0.01 |
| ENSG00000134193 | regenerating islet-derived family, member 4 | REG4 | 7.70602E-74 | 87.04 | 497.22 | 4.95 |
| ENSG00000169876 | mucin 17, cell surface associated | MUC17 | 3.99853E-73 | 82.50 | 110.99 | 1.16 |
| ENSG00000160182 | trefoil factor 1 | TFF1 | 5.8703E-65 | 78.72 | 521.43 | 6.92 |
| ENSG00000087916 | solute carrier family 6 (amino acid transporter), member 14 | SLC6A14 | 4.40407E-54 | 71.80 | 6.06 | 0.05 |
| ENSG00000140279 | dual oxidase 2 | DUOX2 | 3.71501E-64 | 69.75 | 42.81 | 0.62 |
| ENSG00000109511 | annexin A10 | ANXA10 | 3.92452E-50 | 67.28 | 12.66 | 0.13 |
| ENSG00000179546 | 5-hydroxytryptamine (serotonin) receptor 1D, G protein-coupled | HTR1D | 1.10484E-46 | 63.82 | 7.11 | 0.07 |
| ENSG00000167757 | kallikrein-related peptidase 11 | KLK11 | 2.14986E-37 | 54.63 | 9.82 | 0.17 |
| ENSG00000140274 | dual oxidase maturation factor 2 | DUOXA2 | 4.61842E-50 | 52.83 | 18.54 | 0.29 |
| ENSG00000062038 | cadherin 3, type 1, P-cadherin (placental) | CDH3 | 9.16174E-44 | 50.79 | 4.99 | 0.08 |
| ENSG00000112299 | vanin 1 | VNN1 | 1.9564E-45 | 47.75 | 8.44 | 0.15 |
| ENSG00000198203 | sulfotransferase family, cytosolic, 1C, member 2 | SULT1C2 | 2.03635E-50 | 44.46 | 7.98 | 0.16 |
| ENSG00000161798 | aquaporin 5 | AQP5 | 3.42339E-35 | 37.98 | 3.79 | 0.00 |
| ENSG00000124102 | peptidase inhibitor 3, skin-derived | PI3 | 5.44866E-36 | 33.77 | 88.50 | 3.64 |
| ENSG00000163347 | claudin 1 | CLDN1 | 2.34185E-39 | 32.00 | 8.16 | 0.23 |
| ENSG00000163993 | S100 calcium binding protein P | S100P | 9.6113E-43 | 29.80 | 83.93 | 2.84 |
| ENSG00000120875 | dual specificity phosphatase 4 | DUSP4 | 1.15029E-41 | 29.77 | 5.24 | 0.15 |
| ENSG00000110427 | KIAA1549-like | C11orf41 | 3.38727E-29 | 29.15 | 0.78 | 0.02 |
| ENSG00000198842 | dual specificity phosphatase 27 (putative) | DUSP27 | 1.63174E-17 | 28.85 | 1.42 | 0.07 |
| ENSG00000169248 | chemokine (C-X-C motif) ligand 11 | CXCL11 | 2.29403E-29 | 28.76 | 4.39 | 0.10 |
| ENSG00000189280 | gap junction protein, beta 5, 31.1kDa | GJB5 | 1.3826E-22 | 26.71 | 3.39 | 0.03 |
| ENSG00000163817 | solute carrier family 6 (proline IMINO transporter), member 20 | SLC6A20 | 8.62998E-39 | 25.66 | 7.00 | 0.25 |
| ENSG00000137699 | tripartite motif containing 29 | TRIM29 | 1.02537E-36 | 25.31 | 3.00 | 0.11 |
| ENSG00000005001 | protease, serine, 22 | PRSS22 | 5.60522E-23 | 25.29 | 4.26 | 0.11 |
| ENSG00000184292 | tumor-associated calcium signal transducer 2 | TACSTD2 | 9.62029E-32 | 24.14 | 7.42 | 0.26 |
| ENSG00000110080 | ST3 beta-galactoside alpha-2,3-sialyltransferase 4 | ST3GAL4 | 2.34185E-39 | 22.64 | 12.14 | 0.42 |
| ENSG00000165376 | claudin 2 | CLDN2 | 3.46909E-41 | 22.55 | 5.73 | 0.09 |
| ENSG00000170786 | short chain dehydrogenase/reductase family 16C, member 5 | SDR16C5 | 5.94826E-36 | 21.93 | 14.02 | 0.60 |
| ENSG00000181577 | chromosome 6 open reading frame 223 | C6orf223 | 4.42493E-22 | 20.86 | 1.80 | 0.05 |
| ENSG00000256050 | novel lincRNA | RP11-982M15.6 | 4.4548E-16 | 20.19 | 5.20 | 0.34 |
| ENSG00000136872 | aldolase B, fructose-bisphosphate | ALDOB | 5.14991E-32 | 20.07 | 40.18 | 2.37 |
| ENSG00000214039 | novel lincRNA | RP11-474D1.4 | 8.96388E-18 | 19.45 | 0.61 | 0.00 |
| ENSG00000159184 | homeobox B13 | HOXB13 | 2.43716E-20 | 19.11 | 1.33 | 0.01 |
| ENSG00000135480 | keratin 7 | KRT7 | 1.38454E-22 | 19.00 | 2.05 | 0.02 |
| ENSG00000115648 | melanophilin | MLPH | 1.07795E-22 | 18.79 | 39.47 | 3.86 |
| ENSG00000189433 | gap junction protein, beta 4, 30.3kDa | GJB4 | 9.9407E-21 | 18.45 | 2.05 | 0.04 |
| ENSG00000244503 | known pseudogene | RP11-278L15.6 | 8.18559E-14 | 18.39 | 1.81 | 0.02 |
| ENSG00000084674 | apolipoprotein B (including Ag(x) antigen) | APOB | 1.38326E-22 | 18.39 | 0.29 | 0.00 |
| ENSG00000167653 | prostate stem cell antigen | PSCA | 3.87125E-19 | 18.37 | 2.98 | 0.07 |
| ENSG00000187288 | cell death-inducing DFFA-like effector c | CIDEC | 1.14774E-26 | 17.81 | 9.77 | 0.53 |
| ENSG00000221947 | XK, Kell blood group complex subunit-related family, member 9 | XKR9 | 1.71223E-25 | 17.11 | 3.17 | 0.15 |
| ENSG00000168631 | diffuse panbronchiolitis critical region 1 | DPCR1 | 2.86482E-19 | 16.46 | 0.79 | 0.01 |
| ENSG00000169213 | RAB3B, member RAS oncogene family | RAB3B | 6.26138E-32 | 16.40 | 5.01 | 0.28 |
| ENSG00000230316 | FEZF1 antisense RNA 1 | RP11-560I19.4 | 1.28442E-14 | 16.11 | 0.90 | 0.01 |
| ENSG00000247844 | novel lincRNA | RP11-255B23.3 | 8.38759E-26 | 16.00 | 5.12 | 0.22 |
| ENSG00000130720 | fibrinogen C domain containing 1 | FIBCD1 | 2.36123E-17 | 15.90 | 1.40 | 0.07 |
| ENSG00000188536 | hemoglobin, alpha 2 | HBA2 | 6.86817E-20 | 15.72 | 56.06 | 5.85 |
| ENSG00000187258 | neuropeptide S receptor 1 | NPSR1 | 6.57334E-09 | 15.68 | 0.73 | 0.06 |
| ENSG00000147206 | nuclear RNA export factor 3 | NXF3 | 2.09916E-19 | 15.51 | 1.25 | 0.04 |
| ENSG00000206172 | hemoglobin, alpha 1 | HBA1 | 3.74489E-19 | 15.41 | 56.78 | 6.20 |
| ENSG00000129455 | kallikrein-related peptidase 8 | KLK8 | 1.61722E-16 | 15.18 | 2.80 | 0.01 |
| ENSG00000181791 | novel pseudogene | AC009041.1 | 1.17751E-20 | 15.09 | 4.14 | 0.06 |
| ENSG00000043355 | Zic family member 2 | ZIC2 | 4.38754E-12 | 14.98 | 0.74 | 0.02 |
| ENSG00000162366 | PDZK1 interacting protein 1 | PDZK1IP1 | 1.59145E-20 | 14.92 | 28.07 | 2.88 |
| ENSG00000139800 | Zic family member 5 | ZIC5 | 6.6383E-12 | 14.88 | 0.40 | 0.00 |
| ENSG00000213822 | carcinoembryonic antigen-related cell adhesion molecule 18 | CEACAM18 | 1.23447E-10 | 14.82 | 1.92 | 0.09 |
| ENSG00000248757 | novel lincRNA | CTD-2193G5.1 | 4.08672E-14 | 14.69 | 5.80 | 0.14 |
| ENSG00000163739 | chemokine (C-X-C motif) ligand 1 (melanoma growth stimulating act | CXCL1 | 6.01728E-25 | 14.66 | 26.15 | 1.96 |
| ENSG00000112559 | MyoD family inhibitor | MDFI | 4.56089E-15 | 14.35 | 1.91 | 0.11 |
| ENSG00000119547 | one cut homeobox 2 | ONECUT2 | 1.44599E-20 | 14.29 | 0.56 | 0.03 |
| ENSG00000168955 | transmembrane 4 L six family member 20 | TM4SF20 | 9.10865E-34 | 14.08 | 16.69 | 0.82 |
| ENSG00000188910 | gap junction protein, beta 3, 31kDa | GJB3 | 1.02791E-23 | 13.89 | 5.76 | 0.35 |
| ENSG00000172927 | myeloma overexpressed (in a subset of t(11;14) positive multiple m | MYEOV | 1.424E-22 | 13.84 | 7.00 | 0.58 |
| ENSG00000214318 | ATP synthase, H+ transporting, mitochondrial Fo complex, subunit C | ATP5G1P6 | 6.16112E-14 | 13.69 | 4.85 | 0.04 |
| ENSG00000226926 | known pseudogene | AC010423.1 | 1.74556E-16 | 13.68 | 2.01 | 0.05 |
| ENSG00000167105 | transmembrane protein 92 | TMEM92 | 3.68899E-21 | 13.67 | 5.48 | 0.42 |
| ENSG00000198788 | mucin 2, oligomeric mucus/gel-forming | MUC2 | 2.49302E-27 | 13.52 | 2169.42 | 178.18 |
| ENSG00000239899 | Metazoan signal recognition particle RNA | AC011994.1 | 4.92683E-13 | 13.45 | 257.28 | 41.45 |
| ENSG00000101188 | neurotensin receptor 1 (high affinity) | NTSR1 | 1.71959E-15 | 13.40 | 0.90 | 0.03 |
| ENSG00000134827 | transcobalamin I (vitamin B12 binding protein, R binder family) | TCN1 | 1.19399E-17 | 13.32 | 2.69 | 0.10 |
| ENSG00000243942 |  | AC112721.3 | 2.17833E-11 | 13.27 | 67.26 | 5.92 |
| ENSG00000172031 | epoxide hydrolase 4 | EPHX4 | 5.84349E-15 | 13.24 | 1.22 | 0.03 |
| ENSG00000139515 | pancreatic and duodenal homeobox 1 | PDX1 | 5.49451E-16 | 13.14 | 3.02 | 0.13 |
| ENSG00000134757 | desmoglein 3 | DSG3 | 2.21209E-26 | 12.89 | 1.03 | 0.02 |

| ENSG00000125207 | piwi-like 1 (Drosophila) | PIWIL1 | 1.77396E-15 | 12.84 | 0.75 | 0.03 |
| --- | --- | --- | --- | --- | --- | --- |
| ENSG00000239484 |  | AC112721.1 | 9.90456E-12 | 12.40 | 80.67 | 6.92 |
| ENSG00000139289 | pleckstrin homology-like domain, family A, member 1 | PHLDA1 | 7.399E-25 | 12.30 | 15.85 | 1.36 |
| ENSG00000146038 | doublecortin domain containing 2 | DCDC2 | 4.40987E-20 | 12.27 | 2.39 | 0.17 |
| ENSG00000164379 | forkhead box Q1 | FOXQ1 | 7.11962E-20 | 12.25 | 7.01 | 0.60 |
| ENSG00000151012 | solute carrier family 7 (anionic amino acid transporter light chain, xc | SLC7A11 | 6.67309E-23 | 12.13 | 3.84 | 0.34 |
| ENSG00000226598 | novel lincRNA | AC017060.1 | 7.34204E-13 | 12.02 | 7.55 | 0.38 |
| ENSG00000182851 | glycosylphosphatidylinositol anchored high density lipoprotein bindin | GPIHBP1 | 2.49672E-08 | 12.00 | 2.37 | 0.34 |
| ENSG00000239824 |  | AC112721.2 | 1.80428E-10 | 11.94 | 64.46 | 6.10 |
| ENSG00000060566 | cAMP responsive element binding protein 3-like 3 | CREB3L3 | 3.21949E-14 | 11.76 | 3.45 | 0.35 |
| ENSG00000214049 | urothelial cancer associated 1 (non-protein coding) | UCA1 | 2.48686E-15 | 11.46 | 3.22 | 0.16 |
| ENSG00000244734 | hemoglobin, beta | HBB | 3.73227E-21 | 11.46 | 38.69 | 3.81 |
| ENSG00000204876 | Uncharacterized protein | AC021218.2 | 1.29239E-24 | 11.33 | 7.01 | 0.45 |
| ENSG00000196091 | myosin binding protein C, slow type | MYBPC1 | 1.26487E-16 | 11.26 | 1.00 | 0.05 |
| ENSG00000253368 | TMF1-regulated nuclear protein 1 | TRNP1 | 2.09606E-16 | 11.22 | 2.29 | 0.10 |
| ENSG00000168427 | kelch-like 30 (Drosophila) | KLHL30 | 2.19239E-11 | 11.19 | 0.53 | 0.01 |
| ENSG00000167755 | kallikrein-related peptidase 6 | KLK6 | 3.06137E-14 | 10.87 | 1.25 | 0.00 |
| ENSG00000102934 | plasmolipin | PLLP | 2.95601E-18 | 10.82 | 6.30 | 0.49 |
| ENSG00000136689 | interleukin 1 receptor antagonist | IL1RN | 1.07362E-15 | 10.73 | 4.76 | 0.58 |
| ENSG00000185168 | long intergenic non-protein coding RNA 482 | LINC00482 | 0.000461014 | 10.58 | 4.96 | 1.60 |
| ENSG00000110492 | midkine (neurite growth-promoting factor 2) | MDK | 1.24313E-09 | 10.54 | 16.09 | 3.38 |
| ENSG00000130600 | H19, imprinted maternally expressed transcript (non-protein coding) | H19 | 0.00041609 | 10.33 | 1.74 | 0.46 |
| ENSG00000162009 | somatostatin receptor 5 | SSTR5 | 4.15315E-13 | 10.09 | 0.90 | 0.02 |
| ENSG00000168502 | SOGA family member 2 | CCDC165 | 2.16669E-18 | 9.97 | 1.55 | 0.15 |
| ENSG00000166268 | chromosome 12 open reading frame 28 | C12orf28 | 5.1297E-14 | 9.93 | 3.57 | 0.24 |
| ENSG00000163734 | chemokine (C-X-C motif) ligand 3 | CXCL3 | 4.46176E-19 | 9.91 | 8.91 | 0.87 |
| ENSG00000137203 | transcription factor AP-2 alpha (activating enhancer binding protein 2 | TFAP2A | 7.81108E-11 | 9.87 | 0.54 | 0.05 |
| ENSG00000181634 | tumor necrosis factor (ligand) superfamily, member 15 | TNFSF15 | 5.72378E-22 | 9.81 | 7.40 | 0.62 |
| ENSG00000132911 | neuromedin U receptor 2 | NMUR2 | 8.26628E-11 | 9.78 | 0.56 | 0.00 |
| ENSG00000169894 | mucin 3A, cell surface associated | MUC3A | 1.28813E-17 | 9.71 | 263.63 | 35.49 |
| ENSG00000163191 | S100 calcium binding protein A11 | S100A11 | 1.44236E-13 | 9.68 | 270.57 | 46.32 |
| ENSG00000232903 | putative processed transcript | RP13-137A17.4 | 0.038257699 | 9.43 | 0.55 | 0.13 |
| ENSG00000202538 | RNA, U4 small nuclear 2 | RNU4-2 | 0.015275326 | 9.11 | 70.39 | 27.68 |
| ENSG00000140254 | dual oxidase maturation factor 1 | DUOXA1 | 6.04912E-12 | 9.10 | 1.24 | 0.03 |
| ENSG00000124157 | semenogelin II | SEMG2 | 7.57545E-07 | 9.05 | 0.50 | 0.00 |
| ENSG00000205922 | one cut homeobox 3 | ONECUT3 | 8.73572E-07 | 9.05 | 0.68 | 0.00 |
| ENSG00000137033 | interleukin 33 | IL33 | 2.41111E-16 | 8.96 | 7.34 | 0.91 |
| ENSG00000169429 | interleukin 8 | IL8 | 1.27709E-11 | 8.85 | 1.44 | 0.11 |
| ENSG00000240869 | Metazoan signal recognition particle RNA | AL136303.1 | 2.17461E-12 | 8.84 | 204.74 | 34.31 |
| ENSG00000250991 | MHC class I polypeptide-related sequence E (pseudogene) | MICE | 2.88944E-07 | 8.72 | 3.28 | 0.36 |
| ENSG00000088386 | solute carrier family 15 (oligopeptide transporter), member 1 | SLC15A1 | 4.30646E-14 | 8.55 | 1.80 | 0.17 |
| ENSG00000085117 | CD82 molecule | CD82 | 1.33461E-10 | 8.53 | 12.55 | 2.51 |
| ENSG00000196352 | CD55 molecule, decay accelerating factor for complement (Cromer b | CD55 | 2.76139E-23 | 8.51 | 54.44 | 5.43 |
| ENSG00000105464 | glutamate receptor, ionotropic, N-methyl D-aspartate 2D | GRIN2D | 2.02587E-17 | 8.42 | 1.72 | 0.14 |
| ENSG00000158825 | cytidine deaminase | CDA | 1.44043E-11 | 8.36 | 18.17 | 3.02 |
| ENSG00000111700 | solute carrier organic anion transporter family, member 1B3 | SLCO1B3 | 2.91104E-08 | 8.30 | 0.77 | 0.01 |
| ENSG00000257032 |  |  | 0.043295843 | 8.28 | 2.03 | 0.67 |
| ENSG00000229715 | eukaryotic translation elongation factor 1 delta pseudogene 3 | EEF1DP3 | 4.31228E-11 | 8.21 | 0.86 | 0.07 |
| ENSG00000148346 | lipocalin 2 | LCN2 | 1.11962E-19 | 8.19 | 60.83 | 7.17 |
| ENSG00000081041 | chemokine (C-X-C motif) ligand 2 | CXCL2 | 1.35793E-14 | 8.06 | 5.04 | 0.49 |
| ENSG00000107159 | carbonic anhydrase IX | CA9 | 6.62006E-09 | 8.06 | 2.26 | 0.15 |
| ENSG00000236039 | novel lincRNA | AC019117.2 | 9.90456E-12 | 7.97 | 3.52 | 0.24 |
| ENSG00000184956 | mucin 6, oligomeric mucus/gel-forming | MUC6 | 4.68824E-18 | 7.88 | 6.68 | 0.78 |
| ENSG00000111701 | apolipoprotein B mRNA editing enzyme, catalytic polypeptide 1 | APOBEC1 | 1.15126E-09 | 7.87 | 11.62 | 1.99 |
| ENSG00000256208 | novel miRNA | AL353626.2 | 1.54866E-17 | 7.86 | 8.46 | 0.97 |
| ENSG00000133985 | tetratricopeptide repeat domain 9 | TTC9 | 1.16271E-12 | 7.86 | 3.26 | 0.50 |
| ENSG00000135046 | annexin A1 | ANXA1 | 1.8355E-13 | 7.84 | 23.84 | 4.10 |
| ENSG00000176485 | phospholipase A2, group XVI | PLA2G16 | 5.49276E-08 | 7.80 | 3.32 | 0.69 |
| ENSG00000102854 | mesothelin | MSLN | 7.6286E-14 | 7.75 | 3.35 | 0.38 |
| ENSG00000228974 | known pseudogene | AC006483.5 | 2.55532E-12 | 7.75 | 76.68 | 12.76 |
| ENSG00000164946 | FRAS1 related extracellular matrix 1 | FREM1 | 8.7317E-13 | 7.72 | 2.85 | 0.47 |
| ENSG00000131746 | tensin 4 | TNS4 | 4.4548E-16 | 7.68 | 10.85 | 1.51 |
| ENSG00000183018 | spinster homolog 2 (Drosophila) | SPNS2 | 2.65137E-16 | 7.62 | 11.75 | 1.58 |
| ENSG00000137225 | calpain 11 | CAPN11 | 9.80776E-07 | 7.57 | 0.61 | 0.06 |
| ENSG00000226715 | putative lincRNA | RP11-411H5.1 | 1.06484E-09 | 7.49 | 2.59 | 0.00 |
| ENSG00000184471 | C1q and tumor necrosis factor related protein 8 | C1QTNF8 | 0.000523086 | 7.44 | 0.33 | 0.03 |
| ENSG00000142661 | myomesin family, member 3 | MYOM3 | 1.32733E-13 | 7.43 | 1.03 | 0.12 |
| ENSG00000116741 | regulator of G-protein signaling 2, 24kDa | RGS2 | 2.52098E-17 | 7.43 | 24.34 | 3.06 |
| ENSG00000139629 | UDP-N-acetyl-alpha-D-galactosamine:polypeptide N-acetylgalactosa | GALNT6 | 7.26089E-16 | 7.41 | 7.26 | 0.99 |
| ENSG00000138755 | chemokine (C-X-C motif) ligand 9 | CXCL9 | 1.17215E-13 | 7.31 | 7.19 | 1.14 |
| ENSG00000134824 | fatty acid desaturase 2 | FADS2 | 7.81108E-11 | 7.29 | 1.91 | 0.35 |
| ENSG00000086548 | carcinoembryonic antigen-related cell adhesion molecule 6 (non-spec | CEACAM6 | 5.25566E-13 | 7.28 | 419.73 | 77.24 |
| ENSG00000203635 | novel lincRNA | AC144450.2 | 3.9512E-06 | 7.22 | 0.64 | 0.02 |
| ENSG00000115009 | chemokine (C-C motif) ligand 20 | CCL20 | 2.84259E-16 | 7.20 | 20.55 | 2.64 |
| ENSG00000170231 | fatty acid binding protein 6, ileal | FABP6 | 4.54102E-05 | 7.17 | 1.16 | 0.07 |
| ENSG00000170561 | iroquois homeobox 2 | IRX2 | 1.63033E-08 | 7.13 | 0.29 | 0.00 |
| ENSG00000134070 | interleukin-1 receptor-associated kinase 2 | IRAK2 | 1.17215E-13 | 7.12 | 3.01 | 0.38 |
| ENSG00000173227 | synaptotagmin XII | SYT12 | 1.66298E-08 | 7.06 | 0.27 | 0.03 |
| ENSG00000237632 | S100 calcium binding protein A11 pseudogene 1 | S100A11P1 | 1.01342E-09 | 7.02 | 18.89 | 2.72 |
| ENSG00000159871 | LY6/PLAUR domain containing 5 | LYPD5 | 2.12837E-11 | 6.93 | 1.42 | 0.14 |
| ENSG00000162591 | multiple EGF-like-domains 6 | MEGF6 | 3.89963E-11 | 6.88 | 1.04 | 0.13 |
| ENSG00000228705 | long intergenic non-protein coding RNA 659 | RP5-885L7.10 | 0.000105943 | 6.86 | 1.63 | 0.06 |
| ENSG00000123843 | complement component 4 binding protein, beta | C4BPB | 1.38479E-10 | 6.85 | 2.53 | 0.39 |
| ENSG00000138028 | cell growth regulator with EF-hand domain 1 | CGREF1 | 1.31267E-08 | 6.82 | 1.48 | 0.22 |
| ENSG00000118733 | olfactomedin 3 | OLFM3 | 2.90745E-09 | 6.78 | 0.32 | 0.01 |
| ENSG00000255706 | known pseudogene | AC125634.1 | 3.16072E-15 | 6.74 | 8.11 | 1.10 |
| ENSG00000138772 | annexin A3 | ANXA3 | 1.91053E-15 | 6.73 | 10.85 | 1.60 |

| ENSG00000160183 | transmembrane protease, serine 3 | TMPRSS3 | 3.54112E-12 | 6.65 | 1.27 | 0.16 |
| --- | --- | --- | --- | --- | --- | --- |
| ENSG00000244230 | Metazoan signal recognition particle RNA | AL355679.1 | 0.000339437 | 6.60 | 5.01 | 0.36 |
| ENSG00000175592 | FOS-like antigen 1 | FOSL1 | 6.76243E-10 | 6.59 | 2.16 | 0.25 |
| ENSG00000169403 | platelet-activating factor receptor | PTAFR | 6.2394E-12 | 6.58 | 5.10 | 0.88 |
| ENSG00000228991 | known pseudogene | RP11-318K12.1 | 5.43144E-15 | 6.58 | 10.23 | 1.28 |
| ENSG00000251179 | novel antisense | RP11-893F2.9 | 2.69077E-07 | 6.57 | 2.42 | 0.16 |
| ENSG00000169047 | insulin receptor substrate 1 | IRS1 | 2.88797E-16 | 6.53 | 4.69 | 0.63 |
| ENSG00000131910 | nuclear receptor subfamily 0, group B, member 2 | NR0B2 | 3.85221E-09 | 6.49 | 2.11 | 0.14 |
| ENSG00000100196 | KDEL (Lys-Asp-Glu-Leu) endoplasmic reticulum protein retention rec | KDELR3 | 2.19855E-14 | 6.38 | 19.32 | 2.95 |
| ENSG00000160862 | alpha-2-glycoprotein 1, zinc-binding | AZGP1 | 3.56348E-08 | 6.35 | 6.01 | 1.33 |
| ENSG00000204540 | psoriasis susceptibility 1 candidate 1 | PSORS1C1 | 2.28298E-08 | 6.26 | 5.60 | 1.16 |
| ENSG00000157064 | nicotinamide nucleotide adenylyltransferase 2 | NMNAT2 | 3.57334E-11 | 6.26 | 0.75 | 0.09 |
| ENSG00000239437 | Metazoan signal recognition particle RNA | AC023162.1 | 5.05357E-16 | 6.23 | 172.43 | 22.13 |
| ENSG00000251026 | novel lincRNA | RP11-138J23.1 | 0.000323681 | 6.22 | 1.21 | 0.03 |
| ENSG00000100342 | apolipoprotein L, 1 | APOL1 | 4.65253E-12 | 6.18 | 29.17 | 5.50 |
| ENSG00000199334 | RNA, 5S ribosomal 11 | RN5S11 | 0.017783191 | 6.15 | 6.40 | 0.64 |
| ENSG00000102265 | TIMP metallopeptidase inhibitor 1 | TIMP1 | 2.76873E-10 | 6.13 | 20.94 | 4.32 |
| ENSG00000196611 | matrix metallopeptidase 1 (interstitial collagenase) | MMP1 | 1.49763E-10 | 6.09 | 9.63 | 1.75 |
| ENSG00000128965 | ChaC, cation transport regulator homolog 1 (E. coli) | CHAC1 | 2.0222E-08 | 6.09 | 2.58 | 0.39 |
| ENSG00000112414 | G protein-coupled receptor 126 | GPR126 | 4.92648E-16 | 6.05 | 9.97 | 1.48 |
| ENSG00000124107 | secretory leukocyte peptidase inhibitor | SLPI | 8.66848E-12 | 6.04 | 32.03 | 5.33 |
| ENSG00000205517 | ral guanine nucleotide dissociation stimulator-like 3 | RGL3 | 3.75302E-09 | 6.04 | 2.80 | 0.49 |
| ENSG00000137673 | matrix metallopeptidase 7 (matrilysin, uterine) | MMP7 | 2.27637E-08 | 5.96 | 0.69 | 0.03 |
| ENSG00000198535 | C2 calcium-dependent domain containing 4A | C2CD4A | 3.85221E-09 | 5.96 | 0.92 | 0.11 |
| ENSG00000074211 | protein phosphatase 2, regulatory subunit B, gamma | PPP2R2C | 0.009501606 | 5.93 | 0.09 | 0.01 |
| ENSG00000197956 | S100 calcium binding protein A6 | S100A6 | 2.72752E-05 | 5.88 | 1188.11 | 385.00 |
| ENSG00000172602 | Rho family GTPase 1 | RND1 | 8.93446E-06 | 5.87 | 4.31 | 1.04 |
| ENSG00000199337 | RNA, 5S ribosomal 3 | RN5S3 | 0.00919058 | 5.85 | 5.69 | 0.38 |
| ENSG00000135253 | kielin/chordin-like protein | KCP | 9.18098E-11 | 5.84 | 0.69 | 0.08 |
| ENSG00000223392 | CLDN10 antisense RNA 1 | CLDN10-AS1 | 0.000304091 | 5.83 | 0.79 | 0.02 |
| ENSG00000088992 | tescalcin | TESC | 9.99024E-08 | 5.80 | 1.94 | 0.26 |
| ENSG00000128578 | family with sequence similarity 40, member B | FAM40B | 2.24301E-11 | 5.79 | 1.41 | 0.21 |
| ENSG00000159263 | single-minded homolog 2 (Drosophila) | SIM2 | 7.67932E-09 | 5.76 | 0.42 | 0.05 |
| ENSG00000238290 | novel lincRNA | RP11-431K24.1 | 0.013240885 | 5.76 | 0.88 | 0.21 |
| ENSG00000065833 | malic enzyme 1, NADP(+)-dependent, cytosolic | ME1 | 6.27603E-13 | 5.75 | 12.60 | 2.17 |
| ENSG00000223609 | hemoglobin, delta | HBD | 2.92652E-05 | 5.73 | 2.31 | 0.36 |
| ENSG00000088826 | spermine oxidase | SMOX | 6.95484E-09 | 5.71 | 2.17 | 0.36 |
| ENSG00000244642 | Metazoan signal recognition particle RNA | AP005717.1 | 1.81351E-15 | 5.61 | 225.01 | 30.64 |
| ENSG00000006327 | tumor necrosis factor receptor superfamily, member 12A | TNFRSF12A | 8.70679E-10 | 5.59 | 7.35 | 1.16 |
| ENSG00000167460 | tropomyosin 4 | TPM4 | 0.000101951 | 5.57 | 170.84 | 58.87 |
| ENSG00000213886 | ubiquitin D | UBD | 0.00026609 | 5.54 | 8.03 | 1.95 |
| ENSG00000138823 | microsomal triglyceride transfer protein | MTTP | 6.63137E-07 | 5.49 | 0.35 | 0.03 |
| ENSG00000013588 | G protein-coupled receptor, family C, group 5, member A | GPRC5A | 2.08539E-13 | 5.48 | 83.46 | 15.04 |
| ENSG00000132357 | caspase recruitment domain family, member 6 | CARD6 | 4.55167E-07 | 5.46 | 3.10 | 0.75 |
| ENSG00000204539 | corneodesmosin | CDSN | 0.002037186 | 5.45 | 5.90 | 0.57 |
| ENSG00000175832 | ets variant 4 | ETV4 | 7.08034E-08 | 5.44 | 1.75 | 0.30 |
| ENSG00000198807 | paired box 9 | PAX9 | 1.04223E-05 | 5.44 | 0.33 | 0.02 |
| ENSG00000105388 | carcinoembryonic antigen-related cell adhesion molecule 5 | CEACAM5 | 1.36037E-08 | 5.42 | 1579.19 | 392.13 |
| ENSG00000023171 | GRAM domain containing 1B | GRAMD1B | 5.74701E-10 | 5.41 | 0.94 | 0.16 |
| ENSG00000185567 | AHNAK nucleoprotein 2 | AHNAK2 | 2.22067E-19 | 5.34 | 2.14 | 0.23 |
| ENSG00000087085 | acetylcholinesterase | ACHE | 2.94088E-12 | 5.32 | 6.66 | 1.13 |
| ENSG00000228791 | novel lincRNA | AC012087.2 | 0.000987858 | 5.30 | 1.18 | 0.22 |
| ENSG00000122641 | inhibin, beta A | INHBA | 2.71706E-06 | 5.26 | 0.31 | 0.04 |
| ENSG00000144035 | N-acetyltransferase 8 (GCN5-related, putative) | NAT8 | 0.001664876 | 5.25 | 1.77 | 0.32 |
| ENSG00000173702 | mucin 13, cell surface associated | MUC13 | 2.4755E-06 | 5.24 | 341.91 | 101.48 |
| ENSG00000137860 | solute carrier family 28 (sodium-coupled nucleoside transporter), me | SLC28A2 | 2.71097E-11 | 5.20 | 5.90 | 1.07 |
| ENSG00000160868 | cytochrome P450, family 3, subfamily A, polypeptide 4 | CYP3A4 | 2.0821E-10 | 5.20 | 3.39 | 0.59 |
| ENSG00000224885 | TSSC1 intronic transcript 1 (non-protein coding) | TSSC1-IT1 | 3.60364E-06 | 5.16 | 5.85 | 1.07 |
| ENSG00000248601 | novel antisense | RP11-310I9.1 | 3.33749E-06 | 5.15 | 1.67 | 0.00 |
| ENSG00000257005 | known pseudogene | RP11-972L6.2 | 1.48915E-05 | 5.13 | 12.70 | 2.59 |
| ENSG00000182870 | UDP-N-acetyl-alpha-D-galactosamine:polypeptide N-acetylgalactosa | GALNT9 | 0.009599451 | 5.13 | 0.19 | 0.02 |
| ENSG00000173156 | ras homolog family member D | RHOD | 2.35215E-06 | 5.12 | 4.78 | 1.05 |
| ENSG00000181409 | apoptosis-associated tyrosine kinase | AATK | 8.35474E-10 | 5.09 | 1.49 | 0.26 |
| ENSG00000244300 | novel lincRNA | RP11-475N22.4 | 0.002148751 | 5.08 | 0.52 | 0.07 |
| ENSG00000223552 | novel antisense | RP11-24F11.2 | 0.014540689 | 5.07 | 3.46 | 1.01 |
| ENSG00000169908 | transmembrane 4 L six family member 1 | TM4SF1 | 5.57472E-12 | 5.06 | 18.37 | 3.47 |
| ENSG00000090402 | sucrase-isomaltase (alpha-glucosidase) | SI | 6.02917E-11 | 5.01 | 22.43 | 4.78 |
| ENSG00000202198 | RNA, 7SK small nuclear | RN7SK | 0.017648573 | 5.01 | 326.67 | 145.08 |
| ENSG00000105499 | phospholipase A2, group IVC (cytosolic, calcium-independent) | PLA2G4C | 7.17264E-07 | 4.98 | 2.53 | 0.53 |
| ENSG00000169247 | SH3 domain and tetratricopeptide repeats 2 | SH3TC2 | 2.14735E-08 | 4.97 | 0.34 | 0.06 |
| ENSG00000238279 | novel antisense | BX470102.3 | 0.000392544 | 4.95 | 113.46 | 40.38 |
| ENSG00000200488 | 7SK RNA | 7SK | 0.006988835 | 4.88 | 36.63 | 14.26 |
| ENSG00000138166 | dual specificity phosphatase 5 | DUSP5 | 6.32238E-09 | 4.88 | 19.51 | 4.50 |
| ENSG00000124469 | carcinoembryonic antigen-related cell adhesion molecule 8 | CEACAM8 | 2.81643E-05 | 4.86 | 15.66 | 4.96 |
| ENSG00000197506 | solute carrier family 28 (sodium-coupled nucleoside transporter), me | SLC28A3 | 1.16722E-07 | 4.84 | 0.66 | 0.10 |
| ENSG00000254332 | known pseudogene | GS1-44D20.1 | 0.038800721 | 4.80 | 110.88 | 52.25 |
| ENSG00000137857 | dual oxidase 1 | DUOX1 | 3.57334E-11 | 4.80 | 3.93 | 0.74 |
| ENSG00000240925 | ribosomal protein S20 pseudogene 31 | RPS20P31 | 0.000199346 | 4.80 | 3.98 | 0.50 |
| ENSG00000252678 | ribonuclease P RNA component H1 | RPPH1 | 0.001521492 | 4.78 | 159.76 | 61.20 |
| ENSG00000153292 | G protein-coupled receptor 110 | GPR110 | 3.68515E-05 | 4.78 | 0.78 | 0.23 |
| ENSG00000237223 | sulfotransferase family, cytosolic, 1C, member 2 pseudogene 1 | SULT1C2P1 | 0.00013538 | 4.78 | 0.35 | 0.01 |
| ENSG00000249267 | novel lincRNA | RP5-916L7.1 | 0.000928535 | 4.77 | 0.13 | 0.01 |
| ENSG00000138685 | fibroblast growth factor 2 (basic) | FGF2 | 5.48554E-10 | 4.75 | 1.68 | 0.30 |
| ENSG00000213145 | cysteine-rich protein 1 (intestinal) | CRIP1 | 8.59578E-05 | 4.75 | 10.29 | 3.28 |
| ENSG00000158023 | WD repeat domain 66 | WDR66 | 5.43822E-05 | 4.73 | 0.31 | 0.08 |
| ENSG00000214313 | alpha-2-glycoprotein 1, zinc-binding pseudogene 1 | AZGP1P1 | 0.000172845 | 4.72 | 1.65 | 0.30 |
| ENSG00000125538 | interleukin 1, beta | IL1B | 6.57334E-09 | 4.69 | 2.53 | 0.48 |

| ENSG00000180061 | transmembrane protein 150B | TMEM150B | 2.95833E-05 | 4.68 | 12.56 | 3.64 |
| --- | --- | --- | --- | --- | --- | --- |
| ENSG00000010310 | gastric inhibitory polypeptide receptor | GIPR | 1.29545E-06 | 4.67 | 3.93 | 0.91 |
| ENSG00000029153 | aryl hydrocarbon receptor nuclear translocator-like 2 | ARNTL2 | 4.10516E-08 | 4.67 | 2.49 | 0.56 |
| ENSG00000227788 | known pseudogene | AC012363.8 | 1.03373E-05 | 4.66 | 2.70 | 0.37 |
| ENSG00000133710 | serine peptidase inhibitor, Kazal type 5 | SPINK5 | 2.22517E-09 | 4.66 | 4.09 | 0.81 |
| ENSG00000142484 | transmembrane 4 L six family member 5 | TM4SF5 | 0.002294028 | 4.61 | 7.73 | 2.51 |
| ENSG00000112541 | phosphodiesterase 10A | PDE10A | 3.49374E-07 | 4.61 | 0.46 | 0.08 |
| ENSG00000227597 | known pseudogene | AC019100.7 | 0.000591028 | 4.58 | 0.79 | 0.08 |
| ENSG00000200624 | RNA, 5S ribosomal 6 | RN5S6 | 0.006777643 | 4.54 | 5.41 | 0.25 |
| ENSG00000135919 | serpin peptidase inhibitor, clade E (nexin, plasminogen activator inhi | SERPINE2 | 9.86022E-10 | 4.52 | 2.84 | 0.60 |
| ENSG00000125046 | chromosome 3 open reading frame 32 | C3orf32 | 3.99784E-08 | 4.51 | 1.12 | 0.21 |
| ENSG00000182378 | phosphatidylinositol-specific phospholipase C, X domain containing 1 | PLCXD1 | 3.61185E-08 | 4.51 | 7.31 | 1.79 |
| ENSG00000142627 | EPH receptor A2 | EPHA2 | 4.70137E-09 | 4.49 | 11.75 | 2.74 |
| ENSG00000131037 | EPS8-like 1 | EPS8L1 | 1.4258E-06 | 4.49 | 7.03 | 1.90 |
| ENSG00000199004 | microRNA 21 | MIR21 | 0.003529932 | 4.47 | 27.05 | 6.72 |
| ENSG00000115507 | orthodenticle homeobox 1 | OTX1 | 6.12284E-05 | 4.46 | 0.15 | 0.00 |
| ENSG00000103888 | KIAA1199 | KIAA1199 | 5.14676E-14 | 4.46 | 3.16 | 0.47 |
| ENSG00000197261 | chromosome 6 open reading frame 141 | C6orf141 | 1.9029E-07 | 4.45 | 5.42 | 1.31 |
| ENSG00000185022 | v-maf musculoaponeurotic fibrosarcoma oncogene homolog F (avian) | MAFF | 2.16408E-09 | 4.45 | 5.01 | 1.01 |
| ENSG00000183778 | UDP-Gal:betaGlcNAc beta 1,3-galactosyltransferase, polypeptide 5 | B3GALT5 | 1.14232E-10 | 4.43 | 16.81 | 3.55 |
| ENSG00000228140 | novel lincRNA | RP3-467K16.4 | 0.003806091 | 4.42 | 0.18 | 0.03 |
| ENSG00000185950 | insulin receptor substrate 2 | IRS2 | 7.29891E-09 | 4.41 | 6.64 | 1.57 |
| ENSG00000105976 | met proto-oncogene (hepatocyte growth factor receptor) | MET | 9.3079E-11 | 4.39 | 14.19 | 3.00 |
| ENSG00000167600 | cytochrome P450, family 2, subfamily S, polypeptide 1 | CYP2S1 | 0.000100909 | 4.38 | 19.52 | 6.71 |
| ENSG00000174827 | PDZ domain containing 1 | PDZK1 | 8.60161E-06 | 4.37 | 2.01 | 0.44 |
| ENSG00000225680 | novel lincRNA | AL163953.2 | 0.001004559 | 4.37 | 0.77 | 0.02 |
| ENSG00000152784 | PR domain containing 8 | PRDM8 | 0.000190436 | 4.36 | 0.91 | 0.24 |
| ENSG00000173432 | serum amyloid A1 | SAA1 | 0.013033882 | 4.36 | 1.30 | 0.23 |
| ENSG00000200343 | RNA, 5S ribosomal 8 | RN5S8 | 0.034325273 | 4.36 | 4.84 | 0.38 |
| ENSG00000199352 | RNA, 5S ribosomal 1 | RN5S1 | 0.002850339 | 4.36 | 5.98 | 0.25 |
| ENSG00000171931 | F-box and WD repeat domain containing 10 | FBXW10 | 1.47397E-05 | 4.35 | 0.65 | 0.11 |
| ENSG00000154928 | EPH receptor B1 | EPHB1 | 0.000289132 | 4.35 | 0.25 | 0.04 |
| ENSG00000157613 | cAMP responsive element binding protein 3-like 1 | CREB3L1 | 1.99643E-08 | 4.34 | 27.93 | 6.99 |
| ENSG00000113070 | heparin-binding EGF-like growth factor | HBEGF | 3.65204E-05 | 4.34 | 5.22 | 1.61 |
| ENSG00000248323 | novel lincRNA | RP11-213H15.3 | 4.87736E-05 | 4.34 | 0.37 | 0.03 |
| ENSG00000088836 | solute carrier family 4, sodium borate transporter, member 11 | SLC4A11 | 0.000162963 | 4.33 | 0.39 | 0.06 |
| ENSG00000244384 | Metazoan signal recognition particle RNA | AC073254.1 | 0.048182608 | 4.33 | 6.31 | 1.88 |
| ENSG00000124882 | epiregulin | EREG | 0.000531326 | 4.32 | 0.97 | 0.30 |
| ENSG00000196228 | sulfotransferase family, cytosolic, 1C, member 3 | SULT1C3 | 5.68889E-05 | 4.30 | 1.01 | 0.11 |
| ENSG00000087510 | transcription factor AP-2 gamma (activating enhancer binding protein | TFAP2C | 1.56562E-05 | 4.27 | 0.49 | 0.04 |
| ENSG00000188828 | glycine receptor, alpha 4 | GLRA4 | 0.009383611 | 4.27 | 0.68 | 0.17 |
| ENSG00000184995 | interferon, epsilon | IFNE | 7.73711E-05 | 4.27 | 0.43 | 0.00 |
| ENSG00000254872 | novel lincRNA | RP13-870H17.3 | 0.001177831 | 4.26 | 0.77 | 0.19 |
| ENSG00000162078 | zymogen granule protein 16 homolog B (rat) | ZG16B | 0.003805043 | 4.25 | 12.43 | 4.62 |
| ENSG00000200087 | small nucleolar RNA, H/ACA box 73B | SNORA73B | 0.012281265 | 4.23 | 57.29 | 22.99 |
| ENSG00000184106 | triggering receptor expressed on myeloid cells-like 3, pseudogene | TREML3 | 0.009656624 | 4.22 | 0.30 | 0.00 |
| ENSG00000173467 | anterior gradient 3 homolog (Xenopus laevis) | AGR3 | 0.014247941 | 4.22 | 52.43 | 22.88 |
| ENSG00000138759 | Fraser syndrome 1 | FRAS1 | 3.99784E-08 | 4.21 | 0.35 | 0.07 |
| ENSG00000166670 | matrix metallopeptidase 10 (stromelysin 2) | MMP10 | 0.000851951 | 4.21 | 0.83 | 0.15 |
| ENSG00000129465 | receptor-interacting serine-threonine kinase 3 | RIPK3 | 0.000173749 | 4.21 | 18.84 | 6.48 |
| ENSG00000215512 | known pseudogene | AP005901.1 | 0.0165065 | 4.20 | 0.17 | 0.01 |
| ENSG00000166401 | serpin peptidase inhibitor, clade B (ovalbumin), member 8 | SERPINB8 | 4.67015E-07 | 4.19 | 5.53 | 1.43 |
| ENSG00000005238 | family with sequence similarity 214, member B | KIAA1539 | 5.80812E-06 | 4.19 | 5.82 | 1.73 |
| ENSG00000146054 | tripartite motif containing 7 | TRIM7 | 9.47321E-07 | 4.18 | 0.88 | 0.19 |
| ENSG00000128610 | FEZ family zinc finger 1 | FEZF1 | 0.001457256 | 4.18 | 0.29 | 0.01 |
| ENSG00000088002 | sulfotransferase family, cytosolic, 2B, member 1 | SULT2B1 | 0.004065291 | 4.16 | 2.36 | 0.70 |
| ENSG00000124920 | chromosome 11 open reading frame 9 | C11orf9 | 6.04263E-09 | 4.15 | 4.38 | 1.03 |
| ENSG00000162699 | DnaJ (Hsp40) homolog, subfamily A, member 1 pseudogene 5 | DNAJA1P5 | 0.00068932 | 4.15 | 0.54 | 0.00 |
| ENSG00000040608 | reticulon 4 receptor | RTN4R | 2.15345E-05 | 4.14 | 0.48 | 0.06 |
| ENSG00000077274 | calpain 6 | CAPN6 | 2.04687E-05 | 4.12 | 1.82 | 0.47 |
| ENSG00000204618 | ring finger protein 39 | RNF39 | 0.002037186 | 4.10 | 1.75 | 0.52 |
| ENSG00000010539 | zinc finger protein 200 | ZNF200 | 0.000104776 | 4.09 | 3.74 | 1.17 |
| ENSG00000203499 | novel lincRNA | RP11-429J17.6 | 6.3974E-09 | 4.09 | 2.24 | 0.46 |
| ENSG00000171889 | MIR31 host gene (non-protein coding) | MIR31HG | 0.036895746 | 4.08 | 0.96 | 0.03 |
| ENSG00000177508 | iroquois homeobox 3 | IRX3 | 0.002565617 | 4.08 | 0.20 | 0.00 |
| ENSG00000171060 | chromosome 8 open reading frame 74 | C8orf74 | 0.009711616 | 4.08 | 0.09 | 0.00 |
| ENSG00000177238 | tripartite motif containing 72 | TRIM72 | 0.005385748 | 4.08 | 0.24 | 0.01 |
| ENSG00000213275 | known pseudogene | AP005232.1 | 0.01614901 | 4.07 | 3.59 | 0.84 |
| ENSG00000134317 | grainyhead-like 1 (Drosophila) | GRHL1 | 4.54102E-05 | 4.06 | 0.30 | 0.05 |
| ENSG00000129474 | ajuba LIM protein | JUB | 0.000327453 | 4.05 | 0.75 | 0.19 |
| ENSG00000152669 | cyclin O | CCNO | 0.01501718 | 4.05 | 0.74 | 0.18 |
| ENSG00000164542 | KIAA0895 | KIAA0895 | 1.76955E-06 | 4.05 | 0.89 | 0.20 |
| ENSG00000245694 | colorectal neoplasia differentially expressed (non-protein coding) | CRNDE | 0.009656624 | 4.04 | 0.72 | 0.04 |
| ENSG00000198900 | topoisomerase (DNA) I | TOP1 | 0.032975782 | 4.04 | 69.23 | 32.37 |
| ENSG00000237988 | olfactory receptor, family 2, subfamily I, member 1 pseudogene | OR2I1P | 0.000268732 | 4.03 | 1.38 | 0.21 |
| ENSG00000227959 | novel antisense | RP11-276H7.2 | 0.021111855 | 4.03 | 3.93 | 1.39 |
| ENSG00000197550 | known pseudogene | RP11-460N11.2 | 2.41442E-08 | 4.03 | 2.85 | 0.61 |
| ENSG00000183900 | novel pseudogene | FOXD1 | 0.008706173 | 4.02 | 0.49 | 0.07 |
| ENSG00000189431 | Ras association (RalGDS/AF-6) domain family (N-terminal) member | RASSF10 | 0.030284053 | 4.00 | 0.24 | 0.02 |
| ENSG00000173559 | nucleic acid binding protein 1 | OBFC2A | 0.000516213 | 3.98 | 15.25 | 5.64 |
| ENSG00000254166 | novel lincRNA | RP11-255B23.4 | 0.001148371 | 3.97 | 1.89 | 0.16 |
| ENSG00000164690 | sonic hedgehog | SHH | 6.78888E-07 | 3.94 | 2.25 | 0.55 |
| ENSG00000164171 | integrin, alpha 2 (CD49B, alpha 2 subunit of VLA-2 receptor) | ITGA2 | 2.97708E-10 | 3.93 | 15.61 | 3.44 |
| ENSG00000158055 | grainyhead-like 3 (Drosophila) | GRHL3 | 2.18162E-05 | 3.91 | 0.57 | 0.09 |
| ENSG00000149798 | CDC42 effector protein (Rho GTPase binding) 2 | CDC42EP2 | 1.91676E-05 | 3.91 | 4.06 | 1.14 |
| ENSG00000181652 | autophagy related 9B | ATG9B | 0.00104853 | 3.90 | 0.59 | 0.06 |
| ENSG00000236878 | known pseudogene | AC012363.7 | 2.81643E-05 | 3.90 | 1.97 | 0.16 |

| ENSG00000161249 | dermokine | DMKN | 4.81325E-05 | 3.88 | 0.63 | 0.15 |
| --- | --- | --- | --- | --- | --- | --- |
| ENSG00000168122 | zinc finger protein 355, pseudogene | ZNF355P | 5.06099E-05 | 3.88 | 0.49 | 0.08 |
| ENSG00000167759 | kallikrein-related peptidase 13 | KLK13 | 0.018604337 | 3.87 | 0.63 | 0.14 |
| ENSG00000155265 | golgin A7 family, member B | GOLGA7B | 0.001989436 | 3.86 | 0.65 | 0.13 |
| ENSG00000165388 | zinc finger protein 488 | ZNF488 | 0.001043934 | 3.86 | 0.33 | 0.06 |
| ENSG00000008513 | ST3 beta-galactoside alpha-2,3-sialyltransferase 1 | ST3GAL1 | 2.18436E-07 | 3.86 | 4.82 | 1.26 |
| ENSG00000218991 | cyclin G1 pseudogene 1 | CCNG1P1 | 0.037498561 | 3.84 | 0.46 | 0.03 |
| ENSG00000242247 | ADP-ribosylation factor GTPase activating protein 3 | ARFGAP3 | 9.66383E-08 | 3.84 | 14.32 | 3.70 |
| ENSG00000126458 | related RAS viral (r-ras) oncogene homolog | RRAS | 0.025195008 | 3.83 | 18.09 | 8.02 |
| ENSG00000240233 | Metazoan signal recognition particle RNA | AF111168.1 | 4.38868E-07 | 3.82 | 23.53 | 4.54 |
| ENSG00000072422 | Rho-related BTB domain containing 1 | RHOBTB1 | 1.28897E-07 | 3.81 | 3.11 | 0.77 |
| ENSG00000102962 | chemokine (C-C motif) ligand 22 | CCL22 | 0.030730519 | 3.81 | 2.67 | 1.12 |
| ENSG00000187664 | hyaluronan and proteoglycan link protein 4 | HAPLN4 | 4.63054E-05 | 3.78 | 0.42 | 0.05 |
| ENSG00000106089 | syntaxin 1A (brain) | STX1A | 1.63577E-05 | 3.78 | 1.02 | 0.19 |
| ENSG00000230638 | known pseudogene | RP11-486B10.4 | 0.001577275 | 3.76 | 2.46 | 0.72 |
| ENSG00000205595 | amphiregulin B | AREGB | 4.47627E-09 | 3.76 | 13.65 | 2.96 |
| ENSG00000227318 | known pseudogene | RP11-282E4.1 | 1.48915E-05 | 3.76 | 2.15 | 0.47 |
| ENSG00000169258 | G protein regulated inducer of neurite outgrowth 1 | GPRIN1 | 4.10516E-08 | 3.75 | 2.87 | 0.63 |
| ENSG00000133048 | chitinase 3-like 1 (cartilage glycoprotein-39) | CHI3L1 | 0.003536213 | 3.75 | 0.43 | 0.09 |
| ENSG00000129657 | SEC14-like 1 (S. cerevisiae) | SEC14L1 | 0.006404873 | 3.75 | 14.13 | 5.96 |
| ENSG00000142677 | interleukin 22 receptor, alpha 1 | IL22RA1 | 2.49875E-06 | 3.75 | 14.43 | 4.19 |
| ENSG00000235331 | known pseudogene | RP11-885N19.5 | 0.006241618 | 3.75 | 4.01 | 0.64 |
| ENSG00000171811 | tetratricopeptide repeat domain 40 | C10orf93 | 0.032352132 | 3.74 | 0.07 | 0.01 |
| ENSG00000237794 | topoisomerase (DNA) I pseudogene 1 | TOP1P1 | 0.000589778 | 3.74 | 39.46 | 14.10 |
| ENSG00000147041 | synaptotagmin-like 5 | SYTL5 | 2.25647E-08 | 3.74 | 6.53 | 1.59 |
| ENSG00000128165 | adrenomedullin 2 | ADM2 | 1.04129E-08 | 3.74 | 1.86 | 0.34 |
| ENSG00000197635 | dipeptidyl-peptidase 4 | DPP4 | 7.40954E-08 | 3.73 | 14.34 | 3.68 |
| ENSG00000092929 | unc-13 homolog D (C. elegans) | UNC13D | 8.49857E-08 | 3.73 | 5.56 | 1.40 |
| ENSG00000197930 | ERO1-like (S. cerevisiae) | ERO1L | 9.96593E-08 | 3.71 | 31.23 | 8.40 |
| ENSG00000234065 | MT-ND4 pseudogene 26 | MTND4P26 | 1.24448E-08 | 3.71 | 2.33 | 0.26 |
| ENSG00000106541 | anterior gradient 2 homolog (Xenopus laevis) | AGR2 | 1.70049E-09 | 3.70 | 272.56 | 63.46 |
| ENSG00000139178 | complement component 1, r subcomponent-like | C1RL | 0.002293797 | 3.69 | 10.08 | 3.94 |
| ENSG00000124171 | par-6 partitioning defective 6 homolog beta (C. elegans) | PARD6B | 0.000128545 | 3.68 | 13.41 | 4.61 |
| ENSG00000175130 | MARCKS-like 1 | MARCKSL1 | 0.042536701 | 3.68 | 63.61 | 30.45 |
| ENSG00000250388 | known lincRNA | AC004510.1 | 0.003160128 | 3.68 | 1.36 | 0.16 |
| ENSG00000151090 | thyroid hormone receptor, beta | THRB | 5.07917E-05 | 3.67 | 1.96 | 0.61 |
| ENSG00000227758 | HLA complex group 9 pseudogene 5 | HCG9P5 | 0.036571746 | 3.67 | 4.29 | 0.81 |
| ENSG00000136155 | sciellin | SCEL | 0.000275726 | 3.67 | 0.76 | 0.18 |
| ENSG00000250033 | SLC7A11 antisense RNA 1 | RP11-725C19.1 | 0.000888971 | 3.66 | 0.40 | 0.09 |
| ENSG00000182351 | known pseudogene | AP000358.3 | 0.026417104 | 3.66 | 15.03 | 5.44 |
| ENSG00000243444 | paralemmin 2 | PALM2 | 0.000953993 | 3.65 | 0.18 | 0.04 |
| ENSG00000179869 | ATP-binding cassette, sub-family A (ABC1), member 13 | ABCA13 | 8.69849E-05 | 3.64 | 0.23 | 0.06 |
| ENSG00000236559 | novel antisense | RP11-243J16.7 | 0.000268724 | 3.64 | 10.01 | 2.59 |
| ENSG00000134504 | potassium channel tetramerisation domain containing 1 | KCTD1 | 0.000318306 | 3.64 | 3.10 | 1.02 |
| ENSG00000203799 | coiled-coil domain containing 162, pseudogene | CCDC162P | 2.88944E-07 | 3.63 | 3.33 | 0.80 |
| ENSG00000204538 | psoriasis susceptibility 1 candidate 2 | PSORS1C2 | 0.012281265 | 3.63 | 1.09 | 0.24 |
| ENSG00000137962 | Rho GTPase activating protein 29 | ARHGAP29 | 8.84037E-07 | 3.62 | 1.34 | 0.35 |
| ENSG00000187583 | pleckstrin homology domain containing, family N member 1 | PLEKHN1 | 0.001749929 | 3.62 | 0.57 | 0.11 |
| ENSG00000133519 | zinc finger, DHHC-type containing 8 pseudogene 1 | ZDHHC8P1 | 0.000372383 | 3.61 | 0.52 | 0.10 |
| ENSG00000176395 | carcinoembryonic antigen-related cell adhesion molecule 20 | CEACAM20 | 0.002994364 | 3.61 | 0.42 | 0.05 |
| ENSG00000058085 | laminin, gamma 2 | LAMC2 | 5.02365E-08 | 3.60 | 13.46 | 3.51 |
| ENSG00000109321 | amphiregulin | AREG | 5.07659E-09 | 3.60 | 13.93 | 3.04 |
| ENSG00000213996 | transmembrane 6 superfamily member 2 | TM6SF2 | 2.82718E-09 | 3.59 | 4.39 | 0.78 |
| ENSG00000123095 | basic helix-loop-helix family, member e41 | BHLHE41 | 1.67638E-05 | 3.59 | 11.68 | 3.66 |
| ENSG00000120149 | msh homeobox 2 | MSX2 | 1.47601E-07 | 3.59 | 1.15 | 0.13 |
| ENSG00000239948 | Metazoan signal recognition particle RNA | AC006953.1 | 0.007406237 | 3.58 | 7.20 | 1.95 |
| ENSG00000185761 | ADAMTS-like 5 | ADAMTSL5 | 3.53382E-06 | 3.58 | 1.45 | 0.29 |
| ENSG00000202521 | RNA, 5S ribosomal 7 | RN5S7 | 0.020628669 | 3.58 | 4.70 | 0.25 |
| ENSG00000225328 | novel antisense | AC019100.3 | 0.00384307 | 3.58 | 0.67 | 0.00 |
| ENSG00000161921 | chemokine (C-X-C motif) ligand 16 | CXCL16 | 2.81643E-05 | 3.58 | 14.89 | 4.75 |
| ENSG00000108448 | tripartite motif containing 16-like | TRIM16L | 0.000219114 | 3.57 | 7.13 | 2.33 |
| ENSG00000140297 | glucosaminyl (N-acetyl) transferase 3, mucin type | GCNT3 | 0.000427484 | 3.56 | 198.54 | 74.12 |
| ENSG00000130513 | growth differentiation factor 15 | GDF15 | 9.47486E-05 | 3.56 | 3.75 | 0.93 |
| ENSG00000078114 | nebulette | NEBL | 1.63164E-06 | 3.55 | 1.63 | 0.46 |
| ENSG00000109610 | superoxide dismutase 3, extracellular | SOD3 | 0.000533538 | 3.55 | 8.48 | 2.90 |
| ENSG00000110347 | matrix metallopeptidase 12 (macrophage elastase) | MMP12 | 0.000150004 | 3.55 | 16.55 | 5.72 |
| ENSG00000232070 | chromosome 14 open reading frame 176 | C14orf176 | 0.006474756 | 3.55 | 13.51 | 5.44 |
| ENSG00000215604 | zinc finger protein 962, pseudogene | ZNF962P | 0.000493552 | 3.54 | 0.45 | 0.08 |
| ENSG00000103569 | aquaporin 9 | AQP9 | 4.50973E-06 | 3.54 | 0.34 | 0.02 |
| ENSG00000181649 | pleckstrin homology-like domain, family A, member 2 | PHLDA2 | 4.21725E-05 | 3.54 | 9.20 | 2.63 |
| ENSG00000215864 | neuroblastoma breakpoint family, member 7 | NBPF7 | 9.52304E-06 | 3.54 | 2.95 | 0.74 |
| ENSG00000254911 | small Cajal body-specific RNA 9 | SCARNA9 | 0.002141485 | 3.53 | 12.20 | 3.26 |
| ENSG00000173557 | chromosome 2 open reading frame 70 | C2orf70 | 0.00206485 | 3.53 | 0.34 | 0.04 |
| ENSG00000131019 | UL16 binding protein 3 | ULBP3 | 0.000557172 | 3.52 | 1.00 | 0.19 |
| ENSG00000133962 | catsper channel auxiliary subunit beta | CATSPERB | 6.9299E-06 | 3.52 | 1.81 | 0.47 |
| ENSG00000165810 | butyrophilin-like 9 | BTNL9 | 3.65204E-05 | 3.52 | 0.88 | 0.22 |
| ENSG00000172183 | interferon stimulated exonuclease gene 20kDa | ISG20 | 0.000987858 | 3.51 | 10.62 | 3.91 |
| ENSG00000224621 | novel antisense | RP11-276H7.3 | 0.012165521 | 3.51 | 2.37 | 0.53 |
| ENSG00000182240 | beta-site APP-cleaving enzyme 2 | BACE2 | 2.55453E-07 | 3.51 | 13.11 | 3.57 |
| ENSG00000248706 |  | AC100791.3 | 0.010654076 | 3.51 | 0.20 | 0.04 |
| ENSG00000156313 | retinitis pigmentosa GTPase regulator | RPGR | 0.049751056 | 3.50 | 5.18 | 2.42 |
| ENSG00000183929 | dual specificity phosphatase 5 pseudogene | DUSP5P | 0.033326837 | 3.49 | 0.23 | 0.02 |
| ENSG00000228613 |  | AC144450.1 | 0.010548417 | 3.48 | 0.67 | 0.04 |
| ENSG00000136574 | GATA binding protein 4 | GATA4 | 0.006961899 | 3.48 | 0.10 | 0.00 |
| ENSG00000226380 | known lincRNA | AC058791.1 | 3.95131E-05 | 3.47 | 5.41 | 1.67 |
| ENSG00000255595 | novel lincRNA | RP4-809F18.1 | 2.67346E-05 | 3.47 | 0.69 | 0.01 |
| ENSG00000138347 | myopalladin | MYPN | 0.000121623 | 3.45 | 0.72 | 0.19 |

| ENSG00000163064 | engrailed homeobox 1 | EN1 | 0.001457256 | 3.45 | 0.14 | 0.00 |
| --- | --- | --- | --- | --- | --- | --- |
| ENSG00000173898 | spectrin, beta, non-erythrocytic 2 | SPTBN2 | 9.61907E-06 | 3.44 | 0.78 | 0.20 |
| ENSG00000231826 | novel lincRNA | AC016735.2 | 0.04272395 | 3.43 | 1.47 | 0.59 |
| ENSG00000167767 | keratin 80 | KRT80 | 0.002753264 | 3.43 | 1.21 | 0.40 |
| ENSG00000160870 | cytochrome P450, family 3, subfamily A, polypeptide 7 | CYP3A7 | 0.001449443 | 3.42 | 0.40 | 0.07 |
| ENSG00000170956 | carcinoembryonic antigen-related cell adhesion molecule 3 | CEACAM3 | 0.01102984 | 3.42 | 40.22 | 17.71 |
| ENSG00000214922 | HLA-F antisense RNA 1 | HLA-F-AS1 | 0.002316946 | 3.41 | 1.22 | 0.36 |
| ENSG00000228917 | novel antisense | RP11-544M22.8 | 0.023167491 | 3.41 | 0.41 | 0.03 |
| ENSG00000136542 | UDP-N-acetyl-alpha-D-galactosamine:polypeptide N-acetylgalactosa | GALNT5 | 5.49779E-09 | 3.40 | 21.26 | 5.09 |
| ENSG00000100078 | phospholipase A2, group III | PLA2G3 | 0.004500513 | 3.40 | 0.31 | 0.04 |
| ENSG00000183914 | dynein, axonemal, heavy chain 2 | DNAH2 | 8.06819E-05 | 3.40 | 0.18 | 0.04 |
| ENSG00000198734 | coagulation factor V (proaccelerin, labile factor) | F5 | 1.51862E-05 | 3.40 | 0.98 | 0.25 |
| ENSG00000094755 | gamma-aminobutyric acid (GABA) A receptor, pi | GABRP | 0.003501468 | 3.39 | 0.44 | 0.11 |
| ENSG00000146592 | cAMP responsive element binding protein 5 | CREB5 | 0.000485981 | 3.39 | 0.25 | 0.06 |
| ENSG00000136153 | LIM domain 7 | LMO7 | 3.31034E-07 | 3.36 | 38.42 | 10.73 |
| ENSG00000205277 | mucin 12, cell surface associated | MUC12 | 1.74669E-08 | 3.35 | 203.42 | 52.27 |
| ENSG00000228146 | caspase 16, apoptosis-related cysteine peptidase (putative) | AC108134.2 | 0.019478791 | 3.35 | 0.73 | 0.14 |
| ENSG00000117394 | solute carrier family 2 (facilitated glucose transporter), member 1 | SLC2A1 | 8.06854E-06 | 3.34 | 13.11 | 4.05 |
| ENSG00000124602 | unc-5 homolog C (C. elegans)-like | UNC5CL | 0.000454775 | 3.33 | 1.53 | 0.41 |
| ENSG00000088053 | glycoprotein VI (platelet) | GP6 | 0.012609396 | 3.33 | 0.73 | 0.21 |
| ENSG00000120738 | early growth response 1 | EGR1 | 0.003501468 | 3.33 | 11.65 | 4.79 |
| ENSG00000154217 | phosphatidylinositol transfer protein, cytoplasmic 1 | PITPNC1 | 8.19516E-05 | 3.32 | 5.69 | 1.78 |
| ENSG00000107819 | sideroflexin 3 | SFXN3 | 3.65204E-05 | 3.32 | 3.81 | 1.18 |
| ENSG00000114771 | arylacetamide deacetylase | AADAC | 0.031426231 | 3.31 | 0.36 | 0.05 |
| ENSG00000021826 | carbamoyl-phosphate synthase 1, mitochondrial | CPS1 | 9.44439E-14 | 3.31 | 0.41 | 0.03 |
| ENSG00000247381 | PDX1 antisense RNA 1 | RP11-438F9.2 | 0.04665514 | 3.31 | 0.19 | 0.01 |
| ENSG00000130164 | low density lipoprotein receptor | LDLR | 1.18605E-06 | 3.30 | 16.73 | 4.91 |
| ENSG00000138119 | myoferlin | MYOF | 2.91104E-08 | 3.30 | 14.30 | 3.69 |
| ENSG00000075618 | fascin homolog 1, actin-bundling protein (Strongylocentrotus purpura | FSCN1 | 0.000265261 | 3.29 | 2.69 | 0.88 |
| ENSG00000177409 | sterile alpha motif domain containing 9-like | SAMD9L | 7.21788E-06 | 3.28 | 10.34 | 3.19 |
| ENSG00000171346 | keratin 15 | KRT15 | 1.81687E-05 | 3.27 | 1.07 | 0.29 |
| ENSG00000134138 | Meis homeobox 2 | MEIS2 | 0.006643385 | 3.26 | 1.50 | 0.55 |
| ENSG00000128039 | steroid 5 alpha-reductase 3 | SRD5A3 | 9.79827E-06 | 3.26 | 3.97 | 1.18 |
| ENSG00000235162 | chromosome 12 open reading frame 75 | C12orf75 | 0.000147977 | 3.26 | 14.27 | 4.72 |
| ENSG00000229782 | novel antisense | AC118754.4 | 0.037632061 | 3.25 | 1.60 | 0.16 |
| ENSG00000116260 | quiescin Q6 sulfhydryl oxidase 1 | QSOX1 | 6.44209E-07 | 3.25 | 13.96 | 4.04 |
| ENSG00000006453 | BAI1-associated protein 2-like 1 | BAIAP2L1 | 0.000424797 | 3.25 | 25.13 | 9.31 |
| ENSG00000139725 | ras homolog family member F (in filopodia) | RHOF | 0.045027559 | 3.24 | 2.71 | 0.99 |
| ENSG00000003249 | dysbindin (dystrobrevin binding protein 1) domain containing 1 | DBNDD1 | 0.0005814 | 3.24 | 0.99 | 0.24 |
| ENSG00000123684 | lysophosphatidylglycerol acyltransferase 1 | LPGAT1 | 8.84077E-07 | 3.23 | 11.23 | 3.25 |
| ENSG00000250457 |  | AC010976.1 | 7.45169E-05 | 3.23 | 5.05 | 1.53 |
| ENSG00000178719 | glutamate receptor, ionotropic, N-methyl D-aspartate-associated pro | GRINA | 0.002440156 | 3.23 | 11.81 | 4.64 |
| ENSG00000171004 | heparan sulfate 6-O-sulfotransferase 2 | HS6ST2 | 0.005446185 | 3.22 | 0.24 | 0.05 |
| ENSG00000169245 | chemokine (C-X-C motif) ligand 10 | CXCL10 | 4.22386E-11 | 3.22 | 9.30 | 1.65 |
| ENSG00000150086 | glutamate receptor, ionotropic, N-methyl D-aspartate 2B | GRIN2B | 0.005957047 | 3.22 | 0.36 | 0.11 |
| ENSG00000244026 | family with sequence similarity 86, member D, pseudogene | FAM86DP | 0.003594455 | 3.21 | 1.37 | 0.31 |
| ENSG00000079337 | Rap guanine nucleotide exchange factor (GEF) 3 | RAPGEF3 | 1.42498E-05 | 3.21 | 1.24 | 0.37 |
| ENSG00000143013 | LIM domain only 4 | LMO4 | 0.024344463 | 3.21 | 9.98 | 4.55 |
| ENSG00000007171 | nitric oxide synthase 2, inducible | NOS2 | 3.34585E-20 | 3.20 | 14.51 | 1.72 |
| ENSG00000106236 | neuronal pentraxin II | NPTX2 | 0.007560129 | 3.20 | 0.59 | 0.15 |
| ENSG00000247484 |  | AC004923.1 | 1.79542E-05 | 3.20 | 1.01 | 0.24 |
| ENSG00000151364 | potassium channel tetramerisation domain containing 14 | KCTD14 | 0.000287759 | 3.19 | 5.68 | 1.64 |
| ENSG00000132744 | aspartoacylase (aminocyclase) 3 | ACY3 | 0.002928433 | 3.18 | 2.89 | 0.87 |
| ENSG00000112320 | sine oculis binding protein homolog (Drosophila) | SOBP | 0.004265063 | 3.18 | 0.72 | 0.23 |
| ENSG00000116132 | paired related homeobox 1 | PRRX1 | 0.015601354 | 3.18 | 0.48 | 0.16 |
| ENSG00000105355 | perilipin 3 | PLIN3 | 0.006454708 | 3.18 | 21.16 | 9.01 |
| ENSG00000236318 | novel lincRNA | AC019117.1 | 0.038197536 | 3.17 | 0.63 | 0.07 |
| ENSG00000104881 | protein phosphatase 1, regulatory subunit 13 like | PPP1R13L | 1.56779E-05 | 3.17 | 3.75 | 1.07 |
| ENSG00000245532 | nuclear paraspeckle assembly transcript 1 (non-protein coding) | NEAT1 | 0.000482735 | 3.17 | 224.10 | 84.68 |
| ENSG00000142178 | salt-inducible kinase 1 | SIK1 | 0.002925964 | 3.17 | 1.84 | 0.68 |
| ENSG00000225745 | novel processed transcript | AL773572.7 | 3.25057E-05 | 3.17 | 4.55 | 1.20 |
| ENSG00000171067 | chromosome 11 open reading frame 24 | C11orf24 | 0.00266296 | 3.16 | 13.28 | 5.27 |
| ENSG00000249899 | novel antisense | CTD-2175A23.1 | 0.023147101 | 3.14 | 2.75 | 0.71 |
| ENSG00000064666 | calponin 2 | CNN2 | 0.006569368 | 3.14 | 21.96 | 9.31 |
| ENSG00000180801 | arylsulfatase family, member J | ARSJ | 0.000130858 | 3.14 | 1.25 | 0.37 |
| ENSG00000119917 | interferon-induced protein with tetratricopeptide repeats 3 | IFIT3 | 0.01918811 | 3.13 | 5.40 | 2.40 |
| ENSG00000006534 | aldehyde dehydrogenase 3 family, member B1 | ALDH3B1 | 5.28807E-06 | 3.12 | 2.62 | 0.74 |
| ENSG00000134107 | basic helix-loop-helix family, member e40 | BHLHE40 | 0.010792036 | 3.11 | 15.09 | 6.64 |
| ENSG00000168209 | DNA-damage-inducible transcript 4 | DDIT4 | 0.000297355 | 3.11 | 12.18 | 4.28 |
| ENSG00000140859 | kinesin family member C3 | KIFC3 | 0.001664876 | 3.10 | 0.81 | 0.25 |
| ENSG00000189058 | apolipoprotein D | APOD | 0.032975782 | 3.09 | 1.20 | 0.41 |
| ENSG00000078900 | tumor protein p73 | TP73 | 0.030284053 | 3.09 | 0.29 | 0.08 |
| ENSG00000250072 | novel lincRNA | CTC-529P8.1 | 0.01184376 | 3.09 | 0.21 | 0.03 |
| ENSG00000011422 | plasminogen activator, urokinase receptor | PLAUR | 0.000623424 | 3.09 | 15.18 | 5.55 |
| ENSG00000170537 | transmembrane channel-like 7 | TMC7 | 0.000138411 | 3.09 | 4.86 | 1.64 |
| ENSG00000106258 | cytochrome P450, family 3, subfamily A, polypeptide 5 | CYP3A5 | 1.14096E-07 | 3.08 | 29.19 | 7.85 |
| ENSG00000151176 | phospholipase B domain containing 2 | PLBD2 | 0.02141258 | 3.08 | 8.02 | 3.53 |
| ENSG00000164761 | tumor necrosis factor receptor superfamily, member 11b | TNFRSF11B | 6.70824E-06 | 3.07 | 3.56 | 0.95 |
| ENSG00000130827 | plexin A3 | PLXNA3 | 1.25773E-06 | 3.06 | 3.24 | 0.91 |
| ENSG00000147144 | coiled-coil domain containing 120 | CCDC120 | 4.55758E-06 | 3.06 | 3.17 | 0.87 |
| ENSG00000189325 | chromosome 6 open reading frame 222 | C6orf222 | 2.26999E-05 | 3.06 | 5.48 | 1.71 |
| ENSG00000233552 |  | AL139035.1 | 0.000829123 | 3.06 | 0.97 | 0.28 |
| ENSG00000163286 | alkaline phosphatase, placental-like 2 | ALPPL2 | 0.047392836 | 3.06 | 0.32 | 0.07 |
| ENSG00000214226 | chromosome 17 open reading frame 67 | C17orf67 | 0.005844445 | 3.06 | 2.05 | 0.70 |
| ENSG00000240733 | Metazoan signal recognition particle RNA | AL136135.1 | 0.042856303 | 3.05 | 2.33 | 0.38 |
| ENSG00000078081 | lysosomal-associated membrane protein 3 | LAMP3 | 0.021003743 | 3.05 | 2.30 | 0.97 |

| ENSG00000204429 | known pseudogene | RP11-1E11.1 | 0.004280781 | 3.05 | 1.67 | 0.31 |
| --- | --- | --- | --- | --- | --- | --- |
| ENSG00000186188 | omega-3 fatty acid receptor 1 | O3FAR1 | 2.29191E-05 | 3.05 | 5.98 | 1.85 |
| ENSG00000108691 | chemokine (C-C motif) ligand 2 | CCL2 | 0.000648973 | 3.05 | 10.11 | 3.35 |
| ENSG00000173221 | glutaredoxin (thioltransferase) | GLRX | 0.028482182 | 3.05 | 8.47 | 3.91 |
| ENSG00000163577 | eukaryotic translation initiation factor 5A2 | EIF5A2 | 0.00192295 | 3.05 | 0.58 | 0.17 |
| ENSG00000150961 | SEC24 family, member D (S. cerevisiae) | SEC24D | 3.0753E-05 | 3.05 | 7.93 | 2.61 |
| ENSG00000253785 | known pseudogene | CTC-308K20.3 | 0.040411652 | 3.04 | 23.37 | 10.87 |
| ENSG00000221926 | tripartite motif containing 16 | TRIM16 | 0.000194941 | 3.04 | 4.41 | 1.47 |
| ENSG00000123838 | complement component 4 binding protein, alpha | C4BPA | 0.0219575 | 3.04 | 0.81 | 0.27 |
| ENSG00000130475 | FCH domain only 1 | FCHO1 | 0.023260423 | 3.04 | 1.95 | 0.79 |
| ENSG00000252309 | novel miRNA | AL391416.1 | 0.047130202 | 3.04 | 10.04 | 2.11 |
| ENSG00000238060 |  | AP002478.2 | 0.001104164 | 3.04 | 2.12 | 0.65 |
| ENSG00000197085 | NPSR1 antisense RNA 1 | AAA1 | 0.035428319 | 3.03 | 0.10 | 0.02 |
| ENSG00000196511 | thiamin pyrophosphokinase 1 | TPK1 | 5.54449E-05 | 3.02 | 1.53 | 0.47 |
| ENSG00000151929 | BCL2-associated athanogene 3 | BAG3 | 0.004094035 | 3.02 | 8.33 | 3.37 |
| ENSG00000157224 | claudin 12 | CLDN12 | 1.91414E-06 | 3.02 | 13.62 | 4.02 |
| ENSG00000161647 | membrane protein, palmitoylated 3 (MAGUK p55 subfamily member | MPP3 | 0.000548341 | 3.01 | 0.50 | 0.12 |
| ENSG00000188263 | interleukin 17 receptor E-like | IL17REL | 0.006452852 | 3.01 | 0.36 | 0.08 |
| ENSG00000134339 | serum amyloid A2 | SAA2 | 0.009249122 | 3.00 | 0.47 | 0.09 |
| ENSG00000135472 | Fas apoptotic inhibitory molecule 2 | FAIM2 | 0.046394126 | 2.99 | 0.31 | 0.10 |
| ENSG00000199270 | RNA, 5S ribosomal 12 | RN5S12 | 0.048095827 | 2.99 | 5.84 | 0.76 |
| ENSG00000164951 | pyruvate dehyrogenase phosphatase catalytic subunit 1 | PDP1 | 5.35466E-06 | 2.98 | 4.76 | 1.41 |
| ENSG00000248771 | novel lincRNA | RP11-294O2.2 | 0.006626736 | 2.98 | 15.05 | 6.30 |
| ENSG00000014257 | acid phosphatase, prostate | ACPP | 0.016792923 | 2.97 | 2.02 | 0.83 |
| ENSG00000157227 | matrix metallopeptidase 14 (membrane-inserted) | MMP14 | 0.002559121 | 2.97 | 17.52 | 7.08 |
| ENSG00000231566 | novel lincRNA | RP5-1158E12.3 | 0.023146919 | 2.97 | 0.61 | 0.15 |
| ENSG00000219891 | zinc finger and SCAN domain containing 12 pseudogene 1 | ZSCAN12P1 | 0.000591028 | 2.97 | 1.78 | 0.46 |
| ENSG00000187678 | sprouty homolog 4 (Drosophila) | SPRY4 | 0.001045407 | 2.96 | 4.72 | 1.80 |
| ENSG00000118898 | periplakin | PPL | 4.1163E-06 | 2.96 | 3.36 | 0.96 |
| ENSG00000153234 | nuclear receptor subfamily 4, group A, member 2 | NR4A2 | 0.032980588 | 2.96 | 1.61 | 0.67 |
| ENSG00000107338 | Src homology 2 domain containing adaptor protein B | SHB | 0.000210313 | 2.95 | 6.12 | 1.90 |
| ENSG00000145287 | placenta-specific 8 | PLAC8 | 0.012007934 | 2.92 | 113.12 | 49.85 |
| ENSG00000124570 | serpin peptidase inhibitor, clade B (ovalbumin), member 6 | SERPINB6 | 0.013066022 | 2.92 | 26.32 | 11.74 |
| ENSG00000256604 | novel pseudogene | MIR4448 | 0.048879077 | 2.91 | 0.72 | 0.13 |
| ENSG00000253868 | FER1L6 antisense RNA 2 | RP11-959I15.3 | 0.045027559 | 2.91 | 1.02 | 0.33 |
| ENSG00000206344 | HLA complex group 27 (non-protein coding) | HCG27 | 0.000932403 | 2.91 | 2.41 | 0.71 |
| ENSG00000214274 | angiogenin, ribonuclease, RNase A family, 5 | ANG | 0.004774966 | 2.91 | 21.71 | 8.41 |
| ENSG00000228634 | novel lincRNA | RP4-534N18.2 | 0.008684524 | 2.90 | 7.60 | 2.60 |
| ENSG00000237070 | novel antisense | AC005550.3 | 0.042916481 | 2.90 | 1.29 | 0.54 |
| ENSG00000132481 | tripartite motif containing 47 | TRIM47 | 0.001597175 | 2.89 | 4.46 | 1.62 |
| ENSG00000239028 | novel miRNA | AL359955.1 | 0.042960349 | 2.89 | 7.85 | 1.48 |
| ENSG00000158859 | ADAM metallopeptidase with thrombospondin type 1 motif, 4 | ADAMTS4 | 0.043154869 | 2.89 | 0.30 | 0.10 |
| ENSG00000188306 | leucine-rich repeats and IQ motif containing 4 | LRRIQ4 | 0.005957047 | 2.88 | 1.13 | 0.30 |
| ENSG00000183196 | carbohydrate (N-acetylglucosamine 6-O) sulfotransferase 6 | CHST6 | 0.002842108 | 2.87 | 0.72 | 0.24 |
| ENSG00000114019 | angiomotin like 2 | AMOTL2 | 0.000461014 | 2.87 | 2.19 | 0.76 |
| ENSG00000159182 | prostate cancer susceptibility candidate | PRAC | 0.006474756 | 2.87 | 0.88 | 0.00 |
| ENSG00000171552 | BCL2-like 1 | BCL2L1 | 0.001940988 | 2.86 | 17.59 | 7.02 |
| ENSG00000200839 | RNA, 5S ribosomal pseudogene 48 | RN5S48 | 0.006643385 | 2.85 | 3.36 | 0.00 |
| ENSG00000106038 | even-skipped homeobox 1 | EVX1 | 0.021207218 | 2.85 | 0.20 | 0.00 |
| ENSG00000156510 | hexokinase domain containing 1 | HKDC1 | 0.005195113 | 2.84 | 12.74 | 5.39 |
| ENSG00000166123 | glutamic pyruvate transaminase (alanine aminotransferase) 2 | GPT2 | 0.026238835 | 2.83 | 3.51 | 1.58 |
| ENSG00000134278 | spire homolog 1 (Drosophila) | SPIRE1 | 1.57999E-05 | 2.83 | 2.23 | 0.65 |
| ENSG00000162695 | solute carrier family 30 (zinc transporter), member 7 | SLC30A7 | 0.000560424 | 2.83 | 10.45 | 3.94 |
| ENSG00000128203 | aspartate beta-hydroxylase domain containing 2 | ASPHD2 | 0.00136115 | 2.82 | 1.77 | 0.59 |
| ENSG00000160180 | trefoil factor 3 (intestinal) | TFF3 | 0.038293512 | 2.81 | 116.31 | 55.45 |
| ENSG00000171345 | keratin 19 | KRT19 | 0.002148751 | 2.81 | 166.06 | 68.37 |
| ENSG00000117226 | guanylate binding protein 3 | GBP3 | 0.001715039 | 2.81 | 52.58 | 21.04 |
| ENSG00000223549 | MT-ND5 pseudogene 28 | MTND5P28 | 0.000401206 | 2.80 | 1.57 | 0.35 |
| ENSG00000180817 | pyrophosphatase (inorganic) 1 | PPA1 | 0.006543274 | 2.79 | 35.41 | 15.09 |
| ENSG00000132329 | receptor (G protein-coupled) activity modifying protein 1 | RAMP1 | 0.006285511 | 2.79 | 1.07 | 0.21 |
| ENSG00000198959 | transglutaminase 2 (C polypeptide, protein-glutamine-gamma-glutam | TGM2 | 1.69802E-05 | 2.79 | 4.11 | 1.32 |
| ENSG00000100033 | proline dehydrogenase (oxidase) 1 | PRODH | 0.005465077 | 2.79 | 0.34 | 0.09 |
| ENSG00000133321 | retinoic acid receptor responder (tazarotene induced) 3 | RARRES3 | 0.01693183 | 2.79 | 9.71 | 4.13 |
| ENSG00000149256 | odz, odd Oz/ten-m homolog 4 (Drosophila) | ODZ4 | 0.007452414 | 2.78 | 0.14 | 0.04 |
| ENSG00000135702 | carbohydrate (N-acetylglucosamine 6-O) sulfotransferase 5 | CHST5 | 3.70592E-05 | 2.78 | 5.98 | 1.91 |
| ENSG00000148926 | adrenomedullin | ADM | 0.000154251 | 2.78 | 4.84 | 1.60 |
| ENSG00000224959 | novel lincRNA | AC017002.2 | 0.027703682 | 2.77 | 3.52 | 1.29 |
| ENSG00000196754 | S100 calcium binding protein A2 | S100A2 | 0.013033882 | 2.77 | 0.36 | 0.04 |
| ENSG00000150457 | LATS, large tumor suppressor, homolog 2 (Drosophila) | LATS2 | 0.002415508 | 2.77 | 2.48 | 0.95 |
| ENSG00000178381 | zinc finger, AN1-type domain 2A | ZFAND2A | 0.001132006 | 2.76 | 7.67 | 2.76 |
| ENSG00000236397 | DEAD/H (Asp-Glu-Ala-Asp/His) box helicase 11 like 2 | DDX11L2 | 0.001004559 | 2.76 | 1.64 | 0.36 |
| ENSG00000226652 | proteasome 26S subunit, non-ATPase, 10 pseudogene 2 | PSMD10P2 | 0.041719001 | 2.76 | 3.23 | 1.08 |
| ENSG00000133019 | cholinergic receptor, muscarinic 3 | CHRM3 | 0.023838394 | 2.75 | 0.64 | 0.27 |
| ENSG00000236893 | argininosuccinate synthetase 1 pseudogene 7 | ASS1P7 | 0.0395719 | 2.75 | 1.63 | 0.53 |
| ENSG00000145649 | granzyme A (granzyme 1, cytotoxic T-lymphocyte-associated serine | GZMA | 0.040014372 | 2.75 | 6.18 | 2.64 |
| ENSG00000136002 | Rho guanine nucleotide exchange factor (GEF) 4 | ARHGEF4 | 0.044198156 | 2.75 | 0.15 | 0.05 |
| ENSG00000006210 | chemokine (C-X3-C motif) ligand 1 | CX3CL1 | 0.013805375 | 2.74 | 1.12 | 0.42 |
| ENSG00000238273 | novel antisense | AC012360.6 | 0.005586715 | 2.74 | 4.37 | 1.70 |
| ENSG00000134321 | radical S-adenosyl methionine domain containing 2 | RSAD2 | 0.039452661 | 2.74 | 4.16 | 1.96 |
| ENSG00000069535 | monoamine oxidase B | MAOB | 0.043154869 | 2.74 | 2.97 | 1.31 |
| ENSG00000233268 | known pseudogene | RP11-558F24.2 | 0.018739043 | 2.73 | 1.22 | 0.08 |
| ENSG00000140835 | carbohydrate (N-acetylglucosamine 6-O) sulfotransferase 4 | CHST4 | 0.009958334 | 2.72 | 0.81 | 0.22 |
| ENSG00000127324 | tetraspanin 8 | TSPAN8 | 0.001538122 | 2.72 | 449.55 | 179.95 |
| ENSG00000140526 | abhydrolase domain containing 2 | ABHD2 | 9.48135E-05 | 2.72 | 30.43 | 10.75 |
| ENSG00000233488 | Uncharacterized protein | AC108463.1 | 0.026118853 | 2.71 | 0.59 | 0.13 |
| ENSG00000180871 | chemokine (C-X-C motif) receptor 2 | CXCR2 | 0.01188981 | 2.71 | 0.49 | 0.14 |
| ENSG00000198814 | glycerol kinase | GK | 0.002683181 | 2.71 | 9.13 | 3.66 |

| ENSG00000111962 | uronyl-2-sulfotransferase | UST | 0.020669482 | 2.70 | 0.95 | 0.36 |
| --- | --- | --- | --- | --- | --- | --- |
| ENSG00000158125 | xanthine dehydrogenase | XDH | 0.000339437 | 2.70 | 12.25 | 4.59 |
| ENSG00000167880 | envoplakin | EVPL | 0.001853902 | 2.70 | 6.29 | 2.47 |
| ENSG00000172794 | RAB37, member RAS oncogene family | RAB37 | 0.011453655 | 2.70 | 1.37 | 0.53 |
| ENSG00000160408 | ST6 (alpha-N-acetyl-neuraminyl-2,3-beta-galactosyl-1,3)-N-acetylga | ST6GALNAC6 | 0.000364647 | 2.69 | 12.01 | 4.42 |
| ENSG00000151746 | bicaudal D homolog 1 (Drosophila) | BICD1 | 0.027703682 | 2.69 | 3.29 | 1.45 |
| ENSG00000174145 | KIAA1239 | KIAA1239 | 0.018248148 | 2.69 | 0.42 | 0.15 |
| ENSG00000158555 | glycerophosphodiester phosphodiesterase domain containing 5 | GDPD5 | 0.020628669 | 2.69 | 0.31 | 0.11 |
| ENSG00000142871 | cysteine-rich, angiogenic inducer, 61 | CYR61 | 0.004439777 | 2.68 | 1.41 | 0.46 |
| ENSG00000242125 | small nucleolar RNA host gene 3 (non-protein coding) | SNHG3 | 0.002504123 | 2.68 | 13.79 | 5.41 |
| ENSG00000143546 | S100 calcium binding protein A8 | S100A8 | 0.009853513 | 2.67 | 1.06 | 0.23 |
| ENSG00000168785 | tetraspanin 5 | TSPAN5 | 0.002612111 | 2.67 | 0.79 | 0.28 |
| ENSG00000234361 | novel antisense | RP11-52J3.3 | 0.007385015 | 2.66 | 3.18 | 0.91 |
| ENSG00000135744 | angiotensinogen (serpin peptidase inhibitor, clade A, member 8) | AGT | 0.014650013 | 2.66 | 1.27 | 0.42 |
| ENSG00000197279 | zinc finger protein 165 | ZNF165 | 0.001069744 | 2.66 | 4.54 | 1.57 |
| ENSG00000117308 | UDP-galactose-4-epimerase | GALE | 0.004759415 | 2.65 | 10.88 | 4.53 |
| ENSG00000171051 | formyl peptide receptor 1 | FPR1 | 0.000181619 | 2.65 | 0.95 | 0.15 |
| ENSG00000052802 | methylsterol monooxygenase 1 | MSMO1 | 0.000416781 | 2.65 | 15.89 | 5.89 |
| ENSG00000112378 | PERP, TP53 apoptosis effector | PERP | 0.00016485 | 2.65 | 68.35 | 24.54 |
| ENSG00000179588 | zinc finger protein, multitype 1 | ZFPM1 | 0.004950944 | 2.65 | 1.46 | 0.50 |
| ENSG00000125735 | tumor necrosis factor (ligand) superfamily, member 14 | TNFSF14 | 0.028440341 | 2.64 | 1.61 | 0.64 |
| ENSG00000035862 | TIMP metallopeptidase inhibitor 2 | TIMP2 | 0.004017561 | 2.64 | 14.89 | 6.22 |
| ENSG00000143217 | poliovirus receptor-related 4 | PVRL4 | 0.001148371 | 2.64 | 1.30 | 0.41 |
| ENSG00000184557 | suppressor of cytokine signaling 3 | SOCS3 | 0.002110386 | 2.64 | 3.62 | 1.36 |
| ENSG00000099337 | potassium channel, subfamily K, member 6 | KCNK6 | 0.012388014 | 2.63 | 11.40 | 4.95 |
| ENSG00000152056 | adaptor-related protein complex 1, sigma 3 subunit | AP1S3 | 0.000300648 | 2.63 | 1.98 | 0.63 |
| ENSG00000118523 | connective tissue growth factor | CTGF | 0.000753932 | 2.63 | 5.42 | 1.92 |
| ENSG00000182782 | hydroxycarboxylic acid receptor 2 | HCAR2 | 0.017994323 | 2.63 | 0.41 | 0.08 |
| ENSG00000150403 | transmembrane and coiled-coil domains 3 | TMCO3 | 0.013476841 | 2.62 | 8.14 | 3.61 |
| ENSG00000010030 | ets variant 7 | ETV7 | 0.031756776 | 2.62 | 1.28 | 0.48 |
| ENSG00000197119 | solute carrier family 25 (mitochondrial carnitine/acylcarnitine carrier) | SLC25A29 | 0.000865316 | 2.62 | 1.97 | 0.61 |
| ENSG00000180105 | known pseudogene | AC017081.2 | 0.02985428 | 2.61 | 2.75 | 0.93 |
| ENSG00000203697 | calpain 8 | CAPN8 | 0.000163757 | 2.61 | 16.71 | 6.06 |
| ENSG00000104728 | Rho guanine nucleotide exchange factor (GEF) 10 | ARHGEF10 | 0.037216361 | 2.61 | 1.07 | 0.48 |
| ENSG00000205502 | C2 calcium-dependent domain containing 4B | C2CD4B | 0.041863535 | 2.61 | 0.60 | 0.15 |
| ENSG00000220867 | known pseudogene | RP3-403L10.2 | 0.025195008 | 2.61 | 6.47 | 1.98 |
| ENSG00000163220 | S100 calcium binding protein A9 | S100A9 | 0.022477329 | 2.60 | 4.67 | 1.68 |
| ENSG00000168679 | solute carrier family 16, member 4 (monocarboxylic acid transporter | SLC16A4 | 0.000776473 | 2.60 | 4.03 | 1.45 |
| ENSG00000119508 | nuclear receptor subfamily 4, group A, member 3 | NR4A3 | 0.034870406 | 2.59 | 0.41 | 0.15 |
| ENSG00000069011 | paired-like homeodomain 1 | PITX1 | 0.003542195 | 2.59 | 2.46 | 0.92 |
| ENSG00000117228 | guanylate binding protein 1, interferon-inducible | GBP1 | 0.003039421 | 2.59 | 10.53 | 4.38 |
| ENSG00000159363 | ATPase type 13A2 | ATP13A2 | 0.000564606 | 2.59 | 4.40 | 1.63 |
| ENSG00000172137 | calbindin 2 | CALB2 | 0.008259227 | 2.59 | 0.61 | 0.11 |
| ENSG00000170485 | neuronal PAS domain protein 2 | NPAS2 | 0.001328582 | 2.59 | 3.42 | 1.34 |
| ENSG00000241886 | putative processed transcript | RP11-242C19.2 | 0.041186539 | 2.58 | 3.65 | 1.27 |
| ENSG00000109107 | aldolase C, fructose-bisphosphate | ALDOC | 0.009806176 | 2.58 | 2.88 | 1.10 |
| ENSG00000205413 | sterile alpha motif domain containing 9 | SAMD9 | 0.000401206 | 2.58 | 16.67 | 6.20 |
| ENSG00000197442 | mitogen-activated protein kinase kinase kinase 5 | MAP3K5 | 0.000327032 | 2.58 | 9.37 | 3.44 |
| ENSG00000185033 | sema domain, immunoglobulin domain (Ig), transmembrane domain | SEMA4B | 0.002141485 | 2.58 | 14.24 | 5.81 |
| ENSG00000243989 | aminoacylase 1 | ACY1 | 0.002562248 | 2.58 | 4.37 | 1.58 |
| ENSG00000008853 | Rho-related BTB domain containing 2 | RHOBTB2 | 0.020883203 | 2.58 | 2.26 | 0.97 |
| ENSG00000128335 | apolipoprotein L, 2 | APOL2 | 0.006461791 | 2.58 | 13.34 | 5.66 |
| ENSG00000214780 | Uncharacterized protein | AP002380.1 | 0.029391727 | 2.58 | 1.05 | 0.27 |
| ENSG00000137193 | pim-1 oncogene | PIM1 | 0.001711121 | 2.57 | 7.87 | 3.07 |
| ENSG00000249464 | novel lincRNA | RP11-93L9.1 | 0.008161384 | 2.57 | 0.47 | 0.17 |
| ENSG00000125798 | forkhead box A2 | FOXA2 | 0.029093455 | 2.57 | 2.39 | 0.99 |
| ENSG00000104522 | tissue specific transplantation antigen P35B | TSTA3 | 0.005332321 | 2.57 | 7.73 | 3.21 |
| ENSG00000059728 | MAX dimerization protein 1 | MXD1 | 9.15864E-05 | 2.56 | 36.10 | 12.67 |
| ENSG00000166340 | tripeptidyl peptidase I | TPP1 | 0.025195008 | 2.56 | 23.00 | 10.76 |
| ENSG00000103534 | transmembrane channel-like 5 | TMC5 | 0.000587399 | 2.56 | 17.93 | 6.86 |
| ENSG00000228903 | RAS p21 protein activator 4C, pseudogene | AC004985.13 | 0.006162508 | 2.56 | 2.27 | 0.88 |
| ENSG00000208038 | microRNA 492 | MIR492 | 0.029373771 | 2.55 | 134.89 | 58.36 |
| ENSG00000173166 | Ras association (RalGDS/AF-6) and pleckstrin homology domains 1 | RAPH1 | 0.013240885 | 2.55 | 10.23 | 4.61 |
| ENSG00000053438 | neuronatin | NNAT | 0.001052901 | 2.55 | 5.44 | 1.80 |
| ENSG00000151689 | inositol polyphosphate-1-phosphatase | INPP1 | 0.003160128 | 2.54 | 9.29 | 3.77 |
| ENSG00000131620 | anoctamin 1, calcium activated chloride channel | ANO1 | 0.023458709 | 2.54 | 2.26 | 0.99 |
| ENSG00000132470 | integrin, beta 4 | ITGB4 | 0.000681297 | 2.53 | 24.82 | 9.67 |
| ENSG00000188157 | agrin | AGRN | 0.000644137 | 2.53 | 2.94 | 1.11 |
| ENSG00000224388 | BACE2 intronic transcript 1 (non-protein coding) | BACE2-IT1 | 0.037564923 | 2.53 | 9.44 | 3.40 |
| ENSG00000065618 | collagen, type XVII, alpha 1 | COL17A1 | 0.002756793 | 2.53 | 31.38 | 13.04 |
| ENSG00000182158 | cAMP responsive element binding protein 3-like 2 | CREB3L2 | 0.000793067 | 2.52 | 14.31 | 5.57 |
| ENSG00000113645 | WW and C2 domain containing 1 | WWC1 | 5.88671E-05 | 2.52 | 4.42 | 1.47 |
| ENSG00000167800 | T-box 10 | TBX10 | 0.002683181 | 2.52 | 2.41 | 0.73 |
| ENSG00000041353 | RAB27B, member RAS oncogene family | RAB27B | 8.49916E-05 | 2.51 | 5.56 | 1.88 |
| ENSG00000089723 | OTU domain, ubiquitin aldehyde binding 2 | OTUB2 | 0.005465077 | 2.51 | 0.99 | 0.34 |
| ENSG00000131203 | indoleamine 2,3-dioxygenase 1 | IDO1 | 0.000369378 | 2.49 | 2.40 | 0.80 |
| ENSG00000183960 | potassium voltage-gated channel, subfamily H (eag-related), membe | KCNH8 | 0.035417891 | 2.48 | 0.47 | 0.18 |
| ENSG00000187231 | SEC14 and spectrin domains 1 | SESTD1 | 0.002948097 | 2.48 | 2.69 | 1.10 |
| ENSG00000152413 | homer homolog 1 (Drosophila) | HOMER1 | 0.016904464 | 2.48 | 0.47 | 0.17 |
| ENSG00000185436 | interleukin 28 receptor, alpha (interferon, lambda receptor) | IL28RA | 0.046394126 | 2.46 | 3.50 | 1.66 |
| ENSG00000163898 | lipase, member H | LIPH | 0.004948727 | 2.46 | 26.95 | 11.47 |
| ENSG00000233286 | MT-ND3 pseudogene 10 | MTND3P10 | 0.009387177 | 2.46 | 2.53 | 0.35 |
| ENSG00000100228 | RAB36, member RAS oncogene family | RAB36 | 0.023591513 | 2.45 | 0.43 | 0.13 |
| ENSG00000151014 | CCR4 carbon catabolite repression 4-like (S. cerevisiae) | CCRN4L | 0.00406718 | 2.45 | 5.38 | 2.07 |
| ENSG00000101144 | bone morphogenetic protein 7 | BMP7 | 0.031688808 | 2.45 | 0.38 | 0.13 |
| ENSG00000007384 | rhomboid 5 homolog 1 (Drosophila) | RHBDF1 | 0.004133905 | 2.45 | 2.68 | 1.07 |

| ENSG00000171903 | cytochrome P450, family 4, subfamily F, polypeptide 11 | CYP4F11 | 0.009099105 | 2.45 | 3.02 | 1.22 |
| --- | --- | --- | --- | --- | --- | --- |
| ENSG00000182795 | chromosome 1 open reading frame 116 | C1orf116 | 5.09803E-05 | 2.45 | 12.10 | 4.09 |
| ENSG00000181381 | DEAD (Asp-Glu-Ala-Asp) box polypeptide 60-like | DDX60L | 0.000360509 | 2.45 | 6.66 | 2.47 |
| ENSG00000085265 | ficolin (collagen/fibrinogen domain containing) 1 | FCN1 | 0.027301361 | 2.44 | 0.27 | 0.07 |
| ENSG00000162654 | guanylate binding protein 4 | GBP4 | 0.000237933 | 2.44 | 5.23 | 1.90 |
| ENSG00000177076 | alkaline ceramidase 2 | ACER2 | 0.00101682 | 2.44 | 5.64 | 2.03 |
| ENSG00000240541 | TM4SF1 antisense RNA 1 | TM4SF1-AS1 | 0.03180407 | 2.43 | 1.18 | 0.24 |
| ENSG00000120708 | transforming growth factor, beta-induced, 68kDa | TGFBI | 0.011811082 | 2.43 | 7.40 | 3.30 |
| ENSG00000152766 | ankyrin repeat domain 22 | ANKRD22 | 0.006467699 | 2.43 | 8.43 | 3.44 |
| ENSG00000150782 | interleukin 18 (interferon-gamma-inducing factor) | IL18 | 0.014566191 | 2.42 | 15.48 | 6.86 |
| ENSG00000235750 | KIAA0040 | KIAA0040 | 0.009501606 | 2.42 | 8.70 | 3.75 |
| ENSG00000139318 | dual specificity phosphatase 6 | DUSP6 | 0.000378737 | 2.42 | 23.41 | 8.66 |
| ENSG00000002587 | heparan sulfate (glucosamine) 3-O-sulfotransferase 1 | HS3ST1 | 0.049273884 | 2.41 | 0.97 | 0.44 |
| ENSG00000204616 | tripartite motif containing 31 | TRIM31 | 0.029093455 | 2.41 | 40.60 | 19.19 |
| ENSG00000128016 | zinc finger protein 36, C3H type, homolog (mouse) | ZFP36 | 0.010310585 | 2.41 | 40.34 | 17.77 |
| ENSG00000183742 | metastasis associated in colon cancer 1 | MACC1 | 0.009078483 | 2.41 | 6.67 | 2.92 |
| ENSG00000168615 | ADAM metallopeptidase domain 9 | ADAM9 | 0.001711121 | 2.41 | 19.39 | 7.77 |
| ENSG00000130340 | sorting nexin 9 | SNX9 | 0.008448743 | 2.41 | 23.29 | 10.18 |
| ENSG00000134531 | epithelial membrane protein 1 | EMP1 | 0.000878784 | 2.41 | 94.33 | 36.46 |
| ENSG00000120549 | KIAA1217 | KIAA1217 | 0.00541379 | 2.40 | 15.74 | 6.72 |
| ENSG00000229670 | plakophilin 4 pseudogene 1 | PKP4P1 | 0.040202309 | 2.38 | 0.90 | 0.30 |
| ENSG00000152128 | transmembrane protein 163 | TMEM163 | 0.018714089 | 2.38 | 0.27 | 0.08 |
| ENSG00000124762 | cyclin-dependent kinase inhibitor 1A (p21, Cip1) | CDKN1A | 0.034428183 | 2.37 | 44.93 | 21.48 |
| ENSG00000111696 | 5'-nucleotidase domain containing 3 | NT5DC3 | 0.000591028 | 2.37 | 2.38 | 0.87 |
| ENSG00000141497 | zinc finger, MYND-type containing 15 | ZMYND15 | 0.045306666 | 2.37 | 0.72 | 0.23 |
| ENSG00000100522 | glucosamine-phosphate N-acetyltransferase 1 | GNPNAT1 | 0.029939973 | 2.37 | 14.19 | 6.67 |
| ENSG00000106031 | homeobox A13 | HOXA13 | 0.013621994 | 2.37 | 3.38 | 1.48 |
| ENSG00000214271 |  | AC084018.1 | 0.039072815 | 2.37 | 2.13 | 0.80 |
| ENSG00000255398 | hydroxycarboxylic acid receptor 3 | HCAR3 | 0.046465349 | 2.37 | 0.52 | 0.07 |
| ENSG00000163297 | anthrax toxin receptor 2 | ANTXR2 | 0.000816387 | 2.36 | 8.09 | 3.16 |
| ENSG00000214417 | keratin 18 pseudogene 13 | KRT18P13 | 0.033326837 | 2.36 | 11.29 | 5.15 |
| ENSG00000189136 | ubiquitin-conjugating enzyme E2Q family member 2 pseudogene 1 | UBE2Q2P1 | 0.042052237 | 2.36 | 1.61 | 0.66 |
| ENSG00000140105 | tryptophanyl-tRNA synthetase | WARS | 0.021785022 | 2.36 | 26.46 | 12.51 |
| ENSG00000113615 | SEC24 family, member A (S. cerevisiae) | SEC24A | 0.006896658 | 2.35 | 11.96 | 5.19 |
| ENSG00000073910 | furry homolog (Drosophila) | FRY | 0.004349903 | 2.35 | 1.40 | 0.57 |
| ENSG00000214814 | fer-1-like 6 (C. elegans) | FER1L6 | 0.001005139 | 2.35 | 10.52 | 4.10 |
| ENSG00000100170 | solute carrier family 5 (sodium/glucose cotransporter), member 1 | SLC5A1 | 0.006200019 | 2.34 | 5.55 | 2.38 |
| ENSG00000226141 | known pseudogene | AF277315.13 | 0.043295843 | 2.34 | 2.40 | 0.50 |
| ENSG00000112773 | family with sequence similarity 46, member A | FAM46A | 0.005446185 | 2.34 | 10.14 | 4.32 |
| ENSG00000197608 | zinc finger protein 841 | ZNF841 | 0.020040225 | 2.33 | 10.77 | 4.91 |
| ENSG00000232756 | novel antisense | RP5-1185I7.1 | 0.001492626 | 2.33 | 3.43 | 1.20 |
| ENSG00000207418 | RNA, U1 small nuclear 9 | RNU1-9 | 0.023638047 | 2.33 | 50.50 | 21.31 |
| ENSG00000132530 | XIAP associated factor 1 | XAF1 | 0.001051315 | 2.33 | 9.39 | 3.60 |
| ENSG00000116883 | known protein coding | AL591845.1 | 0.036652745 | 2.32 | 1.32 | 0.45 |
| ENSG00000120889 | tumor necrosis factor receptor superfamily, member 10b | TNFRSF10B | 0.004919671 | 2.32 | 7.26 | 3.03 |
| ENSG00000225492 | guanylate binding protein 1, interferon-inducible pseudogene 1 | GBP1P1 | 0.012794261 | 2.32 | 4.83 | 2.06 |
| ENSG00000099194 | stearoyl-CoA desaturase (delta-9-desaturase) | SCD | 0.012007934 | 2.32 | 11.78 | 5.30 |
| ENSG00000176903 | paraneoplastic Ma antigen 1 | PNMA1 | 0.002154857 | 2.32 | 5.84 | 2.26 |
| ENSG00000003147 | islet cell autoantigen 1, 69kDa | ICA1 | 0.002411976 | 2.32 | 7.43 | 2.95 |
| ENSG00000116285 | ERBB receptor feedback inhibitor 1 | ERRFI1 | 0.000111691 | 2.32 | 18.18 | 6.38 |
| ENSG00000213406 | annexin A2 pseudogene 1 | ANXA2P1 | 0.022640791 | 2.31 | 57.36 | 26.35 |
| ENSG00000153395 | lysophosphatidylcholine acyltransferase 1 | LPCAT1 | 0.008111374 | 2.31 | 2.61 | 1.10 |
| ENSG00000224083 | known pseudogene | RP11-39K24.4 | 0.03729171 | 2.30 | 1.36 | 0.50 |
| ENSG00000113319 | Ras protein-specific guanine nucleotide-releasing factor 2 | RASGRF2 | 0.013033522 | 2.30 | 0.96 | 0.40 |
| ENSG00000163874 | zinc finger CCCH-type containing 12A | ZC3H12A | 0.040014372 | 2.30 | 5.33 | 2.51 |
| ENSG00000229807 | X (inactive)-specific transcript (non-protein coding) | XIST | 0.004646675 | 2.29 | 37.37 | 16.21 |
| ENSG00000231991 | annexin A2 pseudogene 2 | ANXA2P2 | 0.021088233 | 2.29 | 305.31 | 141.71 |
| ENSG00000251537 | CMT1A duplicated region transcript 1 protein | RP11-385D13.1 | 0.046465349 | 2.29 | 0.70 | 0.28 |
| ENSG00000162772 | activating transcription factor 3 | ATF3 | 0.014131091 | 2.29 | 3.21 | 1.38 |
| ENSG00000175505 | cardiotrophin-like cytokine factor 1 | CLCF1 | 0.027026076 | 2.29 | 1.69 | 0.62 |
| ENSG00000176907 | chromosome 8 open reading frame 4 | C8orf4 | 0.001148512 | 2.29 | 36.05 | 13.66 |
| ENSG00000225308 | argininosuccinate synthetase 1 pseudogene 11 | ASS1P11 | 0.047207728 | 2.28 | 8.69 | 4.02 |
| ENSG00000169507 | solute carrier family 38, member 11 | SLC38A11 | 0.007848969 | 2.28 | 0.96 | 0.35 |
| ENSG00000164181 | ELOVL fatty acid elongase 7 | ELOVL7 | 0.01011276 | 2.28 | 3.38 | 1.44 |
| ENSG00000073737 | dehydrogenase/reductase (SDR family) member 9 | DHRS9 | 0.002004371 | 2.27 | 48.86 | 19.75 |
| ENSG00000144908 | aldehyde dehydrogenase 1 family, member L1 | ALDH1L1 | 0.005534913 | 2.27 | 2.55 | 1.08 |
| ENSG00000182718 | annexin A2 | ANXA2 | 0.007164893 | 2.27 | 127.47 | 55.58 |
| ENSG00000168032 | ectonucleoside triphosphate diphosphohydrolase 3 | ENTPD3 | 0.045850302 | 2.27 | 0.80 | 0.30 |
| ENSG00000237523 | putative lincRNA | RP11-40F6.2 | 0.013690194 | 2.27 | 1.44 | 0.49 |
| ENSG00000163629 | protein tyrosine phosphatase, non-receptor type 13 (APO-1/CD95 (F | PTPN13 | 0.002427547 | 2.26 | 1.31 | 0.51 |
| ENSG00000216740 | annexin A2 pseudogene 3 | ANXA2P3 | 0.022314749 | 2.26 | 21.56 | 9.70 |
| ENSG00000119899 | solute carrier family 17 (anion/sugar transporter), member 5 | SLC17A5 | 0.001168042 | 2.26 | 9.88 | 3.82 |
| ENSG00000225855 | RUSC1 antisense RNA 1 | RUSC1-AS1 | 0.015634032 | 2.26 | 5.39 | 2.31 |
| ENSG00000135111 | T-box 3 | TBX3 | 0.0041779 | 2.25 | 4.38 | 1.79 |
| ENSG00000137267 | tubulin, beta 2A class IIa | TUBB2A | 0.044551015 | 2.25 | 7.30 | 3.39 |
| ENSG00000178404 | differential display clone 8 isoform 2 | AC100788.1 | 0.040208003 | 2.25 | 1.39 | 0.56 |
| ENSG00000070190 | dual adaptor of phosphotyrosine and 3-phosphoinositides | DAPP1 | 0.024179967 | 2.24 | 2.03 | 0.87 |
| ENSG00000008300 | cadherin, EGF LAG seven-pass G-type receptor 3 (flamingo homolog, | CELSR3 | 0.005561576 | 2.24 | 0.41 | 0.15 |
| ENSG00000167695 | family with sequence similarity 57, member A | FAM57A | 0.013805375 | 2.24 | 3.23 | 1.34 |
| ENSG00000176092 | absent in melanoma 1-like | AIM1L | 0.002309075 | 2.23 | 3.15 | 1.23 |
| ENSG00000155093 | protein tyrosine phosphatase, receptor type, N polypeptide 2 | PTPRN2 | 0.01349003 | 2.23 | 3.01 | 1.31 |
| ENSG00000198088 | nucleoporin 62kDa C-terminal like | NUP62CL | 0.047943805 | 2.21 | 1.85 | 0.75 |
| ENSG00000115641 | four and a half LIM domains 2 | FHL2 | 0.009417296 | 2.21 | 10.05 | 4.45 |
| ENSG00000225074 | long intergenic non-protein coding RNA 261 | LINC00261 | 0.004800862 | 2.21 | 3.67 | 1.52 |
| ENSG00000117472 | tetraspanin 1 | TSPAN1 | 0.02859157 | 2.21 | 194.47 | 92.66 |
| ENSG00000257061 | novel lincRNA | RP4-809F18.2 | 0.009099105 | 2.21 | 0.63 | 0.02 |
| ENSG00000186451 | spermatogenesis associated 12 | SPATA12 | 0.021225494 | 2.20 | 0.64 | 0.18 |

| ENSG00000207389 | RNA, U1 small nuclear 4 | RNU1-4 | 0.045850302 | 2.20 | 47.51 | 21.13 |
| --- | --- | --- | --- | --- | --- | --- |
| ENSG00000254287 | novel antisense | RP11-44K6.4 | 0.037184359 | 2.20 | 1.57 | 0.34 |
| ENSG00000185015 | carbonic anhydrase XIII | CA13 | 0.032539914 | 2.19 | 5.27 | 2.47 |
| ENSG00000164023 | sphingomyelin synthase 2 | SGMS2 | 0.008950972 | 2.19 | 8.57 | 3.77 |
| ENSG00000154845 | protein phosphatase 4, regulatory subunit 1 | PPP4R1 | 0.004919671 | 2.19 | 9.26 | 3.91 |
| ENSG00000204614 | tripartite motif containing 40 | TRIM40 | 0.003488111 | 2.18 | 3.39 | 1.32 |
| ENSG00000198142 | sosondowah ankyrin repeat domain family member C | ANKRD57 | 0.042774979 | 2.18 | 9.28 | 4.49 |
| ENSG00000228676 |  | AC069259.1 | 0.009863878 | 2.18 | 7.31 | 3.05 |
| ENSG00000003400 | caspase 10, apoptosis-related cysteine peptidase | CASP10 | 0.018086699 | 2.18 | 7.87 | 3.64 |
| ENSG00000130202 | poliovirus receptor-related 2 (herpesvirus entry mediator B) | PVRL2 | 0.032352132 | 2.17 | 11.46 | 5.43 |
| ENSG00000115008 | interleukin 1, alpha | IL1A | 0.005090985 | 2.16 | 0.72 | 0.16 |
| ENSG00000175938 | ORAI calcium release-activated calcium modulator 3 | ORAI3 | 0.024752405 | 2.16 | 3.03 | 1.27 |
| ENSG00000034152 | mitogen-activated protein kinase kinase 3 | MAP2K3 | 0.045411725 | 2.16 | 8.15 | 3.95 |
| ENSG00000023445 | baculoviral IAP repeat containing 3 | BIRC3 | 0.025286633 | 2.16 | 13.94 | 6.59 |
| ENSG00000256893 | long intergenic non-protein coding RNA GPRC5A-2 | RP11-392P7.6 | 0.044540532 | 2.16 | 2.15 | 0.76 |
| ENSG00000255353 | known pseudogene | RP11-382M14.1 | 0.031155656 | 2.15 | 8.00 | 3.54 |
| ENSG00000071575 | tribbles homolog 2 (Drosophila) | TRIB2 | 0.045027559 | 2.15 | 2.27 | 1.07 |
| ENSG00000162576 | matrix-remodelling associated 8 | MXRA8 | 0.002505149 | 2.15 | 4.00 | 1.51 |
| ENSG00000243104 | known pseudogene | AC012363.10 | 0.01927465 | 2.14 | 1.89 | 0.16 |
| ENSG00000103855 | CD276 molecule | CD276 | 0.016940011 | 2.14 | 3.83 | 1.69 |
| ENSG00000119535 | colony stimulating factor 3 receptor (granulocyte) | CSF3R | 0.04318642 | 2.14 | 0.43 | 0.18 |
| ENSG00000134324 | lipin 1 | LPIN1 | 0.032352132 | 2.14 | 2.44 | 1.16 |
| ENSG00000207005 | RNA, U1 small nuclear 2 | RNU1-2 | 0.040485574 | 2.13 | 51.74 | 22.79 |
| ENSG00000188158 | Nance-Horan syndrome (congenital cataracts and dental anomalies) | NHS | 0.018058584 | 2.13 | 0.73 | 0.30 |
| ENSG00000136802 | leucine rich repeat containing 8 family, member A | LRRC8A | 0.023259228 | 2.13 | 6.36 | 2.95 |
| ENSG00000218014 | keratin 19 pseudogene 1 | RP11-263F15.1 | 0.016305548 | 2.12 | 39.62 | 18.12 |
| ENSG00000106546 | aryl hydrocarbon receptor | AHR | 0.007688808 | 2.12 | 13.28 | 5.88 |
| ENSG00000090339 | intercellular adhesion molecule 1 | ICAM1 | 0.03661928 | 2.11 | 4.35 | 2.05 |
| ENSG00000119125 | guanine deaminase | GDA | 0.022303756 | 2.11 | 9.32 | 4.38 |
| ENSG00000130707 | argininosuccinate synthase 1 | ASS1 | 0.047729436 | 2.11 | 41.72 | 20.61 |
| ENSG00000115339 | UDP-N-acetyl-alpha-D-galactosamine:polypeptide N-acetylgalactosa | GALNT3 | 0.018535666 | 2.11 | 20.02 | 9.19 |
| ENSG00000248472 | DEAD/H (Asp-Glu-Ala-Asp/His) box helicase 11 like 9 | DDX11L9 | 0.003659024 | 2.11 | 0.89 | 0.13 |
| ENSG00000137166 | forkhead box P4 | FOXP4 | 0.043093791 | 2.11 | 4.91 | 2.36 |
| ENSG00000129467 | adenylate cyclase 4 | ADCY4 | 0.004783699 | 2.10 | 1.33 | 0.49 |
| ENSG00000197121 | post-GPI attachment to proteins 1 | PGAP1 | 0.025688562 | 2.10 | 2.17 | 1.02 |
| ENSG00000170017 | activated leukocyte cell adhesion molecule | ALCAM | 0.036793788 | 2.10 | 3.23 | 1.52 |
| ENSG00000170525 | 6-phosphofructo-2-kinase/fructose-2,6-biphosphatase 3 | PFKFB3 | 0.042525184 | 2.09 | 2.76 | 1.32 |
| ENSG00000228998 | known pseudogene | AC091167.2 | 0.019593711 | 2.09 | 3.71 | 1.60 |
| ENSG00000134851 | transmembrane protein 165 | TMEM165 | 0.016900708 | 2.09 | 7.20 | 3.29 |
| ENSG00000144802 | nuclear factor of kappa light polypeptide gene enhancer in B-cells inh | NFKBIZ | 0.013805375 | 2.09 | 11.90 | 5.43 |
| ENSG00000104549 | squalene epoxidase | SQLE | 0.008277641 | 2.08 | 7.18 | 3.13 |
| ENSG00000197324 | low density lipoprotein receptor-related protein 10 | LRP10 | 0.020283631 | 2.08 | 23.77 | 11.16 |
| ENSG00000122711 | serine peptidase inhibitor, Kazal type 4 | SPINK4 | 3.34324E-05 | 2.08 | 42.27 | 13.51 |
| ENSG00000213694 | sphingosine-1-phosphate receptor 3 | S1PR3 | 0.006174082 | 2.07 | 0.57 | 0.20 |
| ENSG00000178038 | ALS2 C-terminal like | ALS2CL | 0.00508233 | 2.07 | 4.74 | 2.02 |
| ENSG00000164211 | StAR-related lipid transfer (START) domain containing 4 | STARD4 | 0.008706173 | 2.07 | 5.53 | 2.41 |
| ENSG00000181784 | ribonuclease, RNase A family, 4 | RNASE4 | 0.033759624 | 2.07 | 20.90 | 9.84 |
| ENSG00000180398 | multiple coagulation factor deficiency 2 | MCFD2 | 0.040336582 | 2.06 | 9.15 | 4.39 |
| ENSG00000128567 | podocalyxin-like | PODXL | 0.01406087 | 2.06 | 3.33 | 1.48 |
| ENSG00000204634 | TBC1 domain family, member 8 (with GRAM domain) | TBC1D8 | 0.011263008 | 2.05 | 3.37 | 1.47 |
| ENSG00000148175 | stomatin | STOM | 0.03180407 | 2.05 | 18.80 | 8.99 |
| ENSG00000064042 | LIM and calponin homology domains 1 | LIMCH1 | 0.014834783 | 2.05 | 1.71 | 0.75 |
| ENSG00000214193 | SH3 domain containing 21 | SH3D21 | 0.001328582 | 2.05 | 4.33 | 1.62 |
| ENSG00000173193 | poly (ADP-ribose) polymerase family, member 14 | PARP14 | 0.031249689 | 2.04 | 16.15 | 7.81 |
| ENSG00000074276 | cadherin-related family member 2 | CDHR2 | 0.001521492 | 2.04 | 20.46 | 8.22 |
| ENSG00000125347 | interferon regulatory factor 1 | IRF1 | 0.047785177 | 2.04 | 10.39 | 5.17 |
| ENSG00000169129 | actin filament associated protein 1-like 2 | AFAP1L2 | 0.010721363 | 2.04 | 3.48 | 1.49 |
| ENSG00000163975 | antigen p97 (melanoma associated) identified by monoclonal antibod | MFI2 | 0.025322361 | 2.03 | 0.73 | 0.30 |
| ENSG00000106392 | core 1 synthase, glycoprotein-N-acetylgalactosamine 3-beta-galactos | C1GALT1 | 0.041294347 | 2.03 | 5.67 | 2.75 |
| ENSG00000225190 | pleckstrin homology domain containing, family M (with RUN domain) | PLEKHM1 | 0.032232412 | 2.01 | 10.00 | 4.77 |
| ENSG00000164078 | macrophage stimulating 1 receptor (c-met-related tyrosine kinase) | MST1R | 0.021111855 | 2.01 | 9.61 | 4.51 |
| ENSG00000142910 | tubulointerstitial nephritis antigen-like 1 | TINAGL1 | 0.022600862 | 2.00 | 9.74 | 4.53 |
| ENSG00000103089 | fatty acid 2-hydroxylase | FA2H | 0.029727357 | 2.00 | 10.24 | 4.77 |
| ENSG00000167371 | proline-rich transmembrane protein 2 | PRRT2 | 0.01161895 | 2.00 | 10.10 | 4.37 |
| ENSG00000213699 | chromosome 2 open reading frame 18 | C2orf18 | 0.023638047 | 1.99 | 10.69 | 4.90 |
| ENSG00000205885 | C1RL antisense RNA 1 | BC12-49244600F4 | 0.013208631 | 1.98 | 3.44 | 1.50 |
| ENSG00000130589 | Peroxisomal proliferator-activated receptor A-interacting complex 28 | RP4-697K14.7 | 0.014950978 | 1.98 | 1.83 | 0.82 |
| ENSG00000196730 | death-associated protein kinase 1 | DAPK1 | 0.03107869 | 1.96 | 3.14 | 1.49 |
| ENSG00000106688 | solute carrier family 1 (neuronal/epithelial high affinity glutamate tra | SLC1A1 | 0.03223515 | 1.96 | 9.27 | 4.37 |
| ENSG00000188833 | ectonucleoside triphosphate diphosphohydrolase 8 | ENTPD8 | 0.01736153 | 1.95 | 5.55 | 2.41 |
| ENSG00000147676 | mal, T-cell differentiation protein 2 (gene/pseudogene) | MAL2 | 0.005195113 | 1.94 | 68.66 | 29.40 |
| ENSG00000111331 | 2'-5'-oligoadenylate synthetase 3, 100kDa | OAS3 | 0.023147101 | 1.94 | 5.41 | 2.56 |
| ENSG00000196576 | plexin B2 | PLXNB2 | 0.042085026 | 1.93 | 20.28 | 10.00 |
| ENSG00000231087 | farnesyl diphosphate synthase pseudogene 7 | FDPSP7 | 0.002504123 | 1.91 | 13.28 | 4.98 |
| ENSG00000119139 | tight junction protein 2 | TJP2 | 0.049259591 | 1.91 | 18.25 | 9.01 |
| ENSG00000236774 | novel protein coding | RP11-381O7.5 | 0.035240394 | 1.91 | 0.71 | 0.19 |
| ENSG00000178209 | plectin | PLEC | 0.048731121 | 1.90 | 30.41 | 15.25 |
| ENSG00000198113 | torsin family 4, member A | C9orf167 | 0.03201166 | 1.88 | 4.89 | 2.31 |
| ENSG00000157551 | potassium inwardly-rectifying channel, subfamily J, member 15 | KCNJ15 | 0.027703682 | 1.87 | 0.12 | 0.02 |
| ENSG00000169174 | proprotein convertase subtilisin/kexin type 9 | PCSK9 | 0.040645624 | 1.84 | 1.14 | 0.51 |
| ENSG00000168389 | major facilitator superfamily domain containing 2A | MFSD2A | 0.024434204 | 1.84 | 2.78 | 1.24 |
| ENSG00000131389 | solute carrier family 6 (neurotransmitter transporter, taurine), memb SLC6A6 | | 0.026324662 | 1.83 | 1.95 | 0.89 |
| ENSG00000233773 | novel protein coding | RP11-460N11.5 | 0.02515223 | 1.82 | 0.74 | 0.19 |
| ENSG00000085063 | CD59 molecule, complement regulatory protein | CD59 | 0.029718576 | 1.82 | 29.37 | 14.00 |
| ENSG00000196923 | PDZ and LIM domain 7 (enigma) | PDLIM7 | 0.049751056 | 1.81 | 2.01 | 0.91 |
| ENSG00000183086 | GATS protein-like 1 | GATSL1 | 0.038586621 | 1.79 | 2.23 | 0.80 |
| ENSG00000115590 | interleukin 1 receptor, type II | IL1R2 | 0.03060844 | 1.77 | 6.67 | 3.07 |

| ENSG00000167723 | transient receptor potential cation channel, subfamily V, member 3 | TRPV3 | 0.013571864 | 1.76 | 3.19 | 1.39 |
| --- | --- | --- | --- | --- | --- | --- |
| ENSG00000070444 | MAX binding protein | MNT | 0.041226076 | 1.75 | 3.12 | 1.46 |
| ENSG00000233980 | farnesyl diphosphate synthase pseudogene 2 | FDPSP2 | 0.035937851 | 1.74 | 15.67 | 7.11 |
| ENSG00000067082 | Kruppel-like factor 6 | KLF6 | 0.041971728 | 1.70 | 36.33 | 17.87 |
| ENSG00000105639 | Janus kinase 3 | JAK3 | 0.048424333 | 1.68 | 2.97 | 1.43 |
| ENSG00000249923 | novel antisense | XXbac-B444P24.8 | 0.04637685 | 1.64 | 0.16 | 0.00 |
| ENSG00000087266 | SH3-domain binding protein 2 | SH3BP2 | 0.046465349 | 1.59 | 4.21 | 2.08 |
| ENSG00000181617 | follicular dendritic cell secreted protein | C4orf7 | 0.001408154 | 1.59 | 5.45 | 13.15 |
| ENSG00000120217 | CD274 molecule | CD274 | 0.0379928 | 1.53 | 1.29 | 0.57 |
| ENSG00000207834 | novel miRNA | D86994.2 | 0.001521492 | -1.51 | 143.29 | 320.14 |
| ENSG00000165092 | aldehyde dehydrogenase 1 family, member A1 | ALDH1A1 | 0.011015852 | -1.51 | 10.71 | 20.32 |
| ENSG00000106853 | prostaglandin reductase 1 | PTGR1 | 0.036755336 | -1.52 | 3.84 | 6.80 |
| ENSG00000134215 | vav 3 guanine nucleotide exchange factor | VAV3 | 0.03675691 | -1.53 | 4.99 | 8.78 |
| ENSG00000242927 |  | AC073308.1 | 0.048131689 | -1.53 | 194.38 | 352.30 |
| ENSG00000165475 | crystallin, lambda 1 | CRYL1 | 0.049760622 | -1.56 | 11.08 | 19.23 |
| ENSG00000007306 | carcinoembryonic antigen-related cell adhesion molecule 7 | CEACAM7 | 0.000974969 | -1.56 | 238.29 | 514.07 |
| ENSG00000082212 | malic enzyme 2, NAD(+)-dependent, mitochondrial | ME2 | 0.04148671 | -1.57 | 7.21 | 12.60 |
| ENSG00000183878 | ubiquitously transcribed tetratricopeptide repeat gene, Y-linked | UTY | 0.006988835 | -1.59 | 1.00 | 2.05 |
| ENSG00000151834 | gamma-aminobutyric acid (GABA) A receptor, alpha 2 | GABRA2 | 0.002842108 | -1.59 | 0.26 | 0.60 |
| ENSG00000230097 | known pseudogene | RP11-460C6.1 | 0.031249689 | -1.60 | 1.83 | 3.58 |
| ENSG00000108187 | phenazine biosynthesis-like protein domain containing | PBLD | 0.005368081 | -1.61 | 5.48 | 10.94 |
| ENSG00000148357 | hemicentin 2 | HMCN2 | 0.036348275 | -1.63 | 0.47 | 0.92 |
| ENSG00000187699 | chromosome 2 open reading frame 88 | C2orf88 | 0.045850302 | -1.63 | 5.31 | 9.25 |
| ENSG00000075239 | acetyl-CoA acetyltransferase 1 | ACAT1 | 0.000648892 | -1.64 | 4.01 | 8.99 |
| ENSG00000118515 | serum/glucocorticoid regulated kinase 1 | SGK1 | 0.041226076 | -1.64 | 2.21 | 3.94 |
| ENSG00000187097 | ectonucleoside triphosphate diphosphohydrolase 5 | ENTPD5 | 0.000177655 | -1.64 | 28.67 | 67.70 |
| ENSG00000189223 | novel lincRNA | AC016683.6 | 0.004019646 | -1.66 | 1.34 | 2.91 |
| ENSG00000164649 | cell division cycle associated 7-like | CDCA7L | 0.009656624 | -1.66 | 2.30 | 4.64 |
| ENSG00000131069 | acyl-CoA synthetase short-chain family member 2 | ACSS2 | 0.043807957 | -1.67 | 12.42 | 21.68 |
| ENSG00000198417 | metallothionein 1F | MT1F | 1.27837E-05 | -1.67 | 8.68 | 25.92 |
| ENSG00000172270 | basigin (Ok blood group) | BSG | 0.021088233 | -1.67 | 53.42 | 97.29 |
| ENSG00000141098 | glucose-fructose oxidoreductase domain containing 2 | GFOD2 | 0.046262245 | -1.67 | 1.70 | 3.22 |
| ENSG00000211973 | immunoglobulin heavy variable 1-69 | IGHV1-69 | 0.034819377 | -1.67 | 71.90 | 127.46 |
| ENSG00000073734 | ATP-binding cassette, sub-family B (MDR/TAP), member 11 | ABCB11 | 0.031756776 | -1.67 | 0.64 | 1.26 |
| ENSG00000122694 | GLI pathogenesis-related 2 | GLIPR2 | 0.044440128 | -1.68 | 1.91 | 3.51 |
| ENSG00000166816 | lactate dehydrogenase D | LDHD | 0.036061484 | -1.69 | 3.30 | 6.12 |
| ENSG00000184307 | zinc finger, DHHC-type containing 23 | ZDHHC23 | 0.037184359 | -1.69 | 2.30 | 4.19 |
| ENSG00000005882 | pyruvate dehydrogenase kinase, isozyme 2 | PDK2 | 0.046228404 | -1.70 | 2.10 | 3.78 |
| ENSG00000232911 | long intergenic non-protein coding RNA C20orf187-2 | RP4-697P8.2 | 0.007908915 | -1.70 | 2.02 | 6.49 |
| ENSG00000168079 | scavenger receptor class A, member 5 (putative) | SCARA5 | 0.04466515 | -1.70 | 2.48 | 4.47 |
| ENSG00000254003 | novel antisense | CTB-167B5.1 | 0.042260088 | -1.72 | 13.63 | 25.00 |
| ENSG00000256612 | CYP2B protein; Cytochrome P450 2B7 short isoform; Uncharacterize | AC008537.3 | 0.002842108 | -1.72 | 1.79 | 4.24 |
| ENSG00000184349 | ephrin-A5 | EFNA5 | 0.009078281 | -1.73 | 0.71 | 1.51 |
| ENSG00000164176 | EGF-like repeats and discoidin I-like domains 3 | EDIL3 | 0.036895746 | -1.73 | 3.64 | 6.55 |
| ENSG00000054277 | opsin 3 | OPN3 | 0.034962292 | -1.73 | 1.94 | 3.79 |
| ENSG00000040199 | PH domain and leucine rich repeat protein phosphatase 2 | PHLPP2 | 0.040558996 | -1.74 | 5.49 | 9.67 |
| ENSG00000184368 | MAP7 domain containing 2 | MAP7D2 | 5.59827E-05 | -1.74 | 0.13 | 0.56 |
| ENSG00000177380 | protein tyrosine phosphatase, receptor type, f polypeptide (PTPRF), i | PPFIA3 | 0.007954401 | -1.74 | 0.68 | 1.49 |
| ENSG00000005187 | acyl-CoA synthetase medium-chain family member 3 | ACSM3 | 0.016168421 | -1.75 | 3.99 | 7.71 |
| ENSG00000101938 | chordin-like 1 | CHRDL1 | 0.039355248 | -1.75 | 0.19 | 0.47 |
| ENSG00000184719 | renalase, FAD-dependent amine oxidase | RNLS | 0.023848403 | -1.76 | 0.54 | 1.15 |
| ENSG00000215572 | estrogen-related receptor alpha pseudogene 1 | ESRRAP1 | 0.044986126 | -1.76 | 1.05 | 2.29 |
| ENSG00000176273 | solute carrier family 35, member G1 | SLC35G1 | 0.032460021 | -1.76 | 0.94 | 1.79 |
| ENSG00000211934 | immunoglobulin heavy variable 1-2 | IGHV1-2 | 0.049028398 | -1.76 | 49.59 | 86.73 |
| ENSG00000162076 | FLYWCH family member 2 | FLYWCH2 | 0.038197536 | -1.76 | 2.09 | 4.26 |
| ENSG00000250305 | Putative methyltransferase KIAA1456 | KIAA1456 | 0.04803614 | -1.77 | 0.40 | 0.76 |
| ENSG00000138796 | hydroxyacyl-CoA dehydrogenase | HADH | 0.002272478 | -1.77 | 8.84 | 18.59 |
| ENSG00000185437 | SH3 domain binding glutamic acid-rich protein | SH3BGR | 0.035417891 | -1.78 | 0.34 | 0.98 |
| ENSG00000131002 | taxilin gamma 2, pseudogene | CYorf15B | 8.06819E-05 | -1.79 | 0.57 | 1.60 |
| ENSG00000176894 | peroxisomal membrane protein 2, 22kDa | PXMP2 | 0.028819799 | -1.79 | 3.72 | 7.33 |
| ENSG00000205189 | zinc finger and BTB domain containing 10 | ZBTB10 | 0.039666511 | -1.79 | 1.83 | 3.26 |
| ENSG00000153790 | chromosome 7 open reading frame 31 | C7orf31 | 0.008631636 | -1.79 | 1.31 | 2.72 |
| ENSG00000185630 | pre-B-cell leukemia homeobox 1 | PBX1 | 0.047130202 | -1.79 | 2.08 | 3.65 |
| ENSG00000104081 | Bcl2 modifying factor | BMF | 0.01869229 | -1.80 | 2.41 | 4.56 |
| ENSG00000168477 | tenascin XB | TNXB | 0.047268139 | -1.81 | 0.43 | 0.79 |
| ENSG00000108700 | chemokine (C-C motif) ligand 8 | CCL8 | 0.034870406 | -1.81 | 1.22 | 2.65 |
| ENSG00000197375 | solute carrier family 22 (organic cation/carnitine transporter), memb | SLC22A5 | 0.012281265 | -1.81 | 1.68 | 3.28 |
| ENSG00000135549 | protein kinase (cAMP-dependent, catalytic) inhibitor beta | PKIB | 4.25934E-06 | -1.82 | 9.45 | 26.58 |
| ENSG00000147650 | low density lipoprotein receptor-related protein 12 | LRP12 | 0.02551952 | -1.82 | 0.56 | 1.09 |
| ENSG00000255774 | novel lincRNA | AP000439.3 | 0.000945224 | -1.82 | 4.04 | 10.11 |
| ENSG00000243955 | glutathione S-transferase alpha 1 | GSTA1 | 0.002825625 | -1.83 | 1.31 | 3.53 |
| ENSG00000095209 | transmembrane protein 38B | TMEM38B | 0.012593765 | -1.83 | 1.06 | 2.17 |
| ENSG00000187193 | metallothionein 1X | MT1X | 9.17244E-05 | -1.83 | 9.75 | 26.19 |
| ENSG00000189221 | monoamine oxidase A | MAOA | 0.001970185 | -1.83 | 20.48 | 43.13 |
| ENSG00000104419 | N-myc downstream regulated 1 | NDRG1 | 0.001347352 | -1.84 | 11.33 | 24.36 |
| ENSG00000135362 | proline rich 5 like | PRR5L | 0.020541365 | -1.84 | 5.05 | 9.40 |
| ENSG00000197766 | complement factor D (adipsin) | CFD | 0.03073502 | -1.84 | 2.64 | 5.29 |
| ENSG00000175084 | desmin | DES | 0.028074524 | -1.84 | 1.20 | 2.43 |
| ENSG00000165269 | aquaporin 7 | AQP7 | 0.034305993 | -1.84 | 0.88 | 1.86 |
| ENSG00000225329 | novel lincRNA | RP11-325F22.5 | 0.031666736 | -1.84 | 11.01 | 20.50 |
| ENSG00000079739 | phosphoglucomutase 1 | PGM1 | 0.039973631 | -1.85 | 4.95 | 8.86 |
| ENSG00000165238 | WNK lysine deficient protein kinase 2 | WNK2 | 0.021088233 | -1.87 | 1.92 | 3.60 |
| ENSG00000241093 |  | AC021914.1 | 0.035413284 | -1.87 | 107.27 | 202.82 |
| ENSG00000044012 | guanylate cyclase activator 2B (uroguanylin) | GUCA2B | 0.000136593 | -1.87 | 9.16 | 23.62 |
| ENSG00000165556 | caudal type homeobox 2 | CDX2 | 0.010164406 | -1.87 | 17.70 | 34.11 |
| ENSG00000166391 | monoacylglycerol O-acyltransferase 2 | MOGAT2 | 0.013240885 | -1.88 | 5.04 | 9.73 |
| ENSG00000182253 | synemin, intermediate filament protein | SYNM | 0.024555874 | -1.89 | 0.55 | 1.10 |

| ENSG00000049246 | period homolog 3 (Drosophila) | PER3 | 0.001466796 | -1.91 | 1.17 | 2.69 |
| --- | --- | --- | --- | --- | --- | --- |
| ENSG00000248380 | novel lincRNA | RP11-94C24.3 | 0.005940017 | -1.91 | 0.59 | 1.47 |
| ENSG00000119042 | SATB homeobox 2 | SATB2 | 0.01502843 | -1.91 | 11.08 | 20.85 |
| ENSG00000144136 | solute carrier family 20 (phosphate transporter), member 1 | SLC20A1 | 0.010164406 | -1.91 | 15.69 | 30.18 |
| ENSG00000168229 | prostaglandin D2 receptor (DP) | PTGDR | 0.039423774 | -1.91 | 1.87 | 3.53 |
| ENSG00000137634 | neurexophilin and PC-esterase domain family, member 4 | FAM55D | 0.004232 | -1.92 | 21.01 | 42.66 |
| ENSG00000185532 | protein kinase, cGMP-dependent, type I | PRKG1 | 0.027037249 | -1.92 | 1.15 | 2.17 |
| ENSG00000153898 | mucolipin 2 | MCOLN2 | 0.009393292 | -1.92 | 1.26 | 2.66 |
| ENSG00000132561 | matrilin 2 | MATN2 | 0.002505149 | -1.93 | 2.84 | 6.01 |
| ENSG00000187091 | phospholipase C, delta 1 | PLCD1 | 0.004837174 | -1.93 | 2.26 | 4.73 |
| ENSG00000197273 | guanylate cyclase activator 2A (guanylin) | GUCA2A | 2.67346E-05 | -1.93 | 48.13 | 124.76 |
| ENSG00000164296 | tigger transposable element derived 6 | TIGD6 | 0.044284455 | -1.94 | 1.10 | 2.10 |
| ENSG00000198125 | myoglobin | MB | 3.06884E-06 | -1.94 | 0.53 | 1.96 |
| ENSG00000145945 | family with sequence similarity 50, member B | FAM50B | 0.026246452 | -1.95 | 0.33 | 0.91 |
| ENSG00000137727 | Rho GTPase activating protein 20 | ARHGAP20 | 0.043295843 | -1.95 | 0.30 | 0.61 |
| ENSG00000171612 | solute carrier family 25 (pyrimidine nucleotide carrier), member 33 | SLC25A33 | 0.000148442 | -1.95 | 2.54 | 6.81 |
| ENSG00000152952 | procollagen-lysine, 2-oxoglutarate 5-dioxygenase 2 | PLOD2 | 0.003869647 | -1.95 | 4.41 | 9.08 |
| ENSG00000172828 | carboxylesterase 3 | CES3 | 0.004136443 | -1.96 | 4.42 | 9.16 |
| ENSG00000148842 | cyclin M2 | CNNM2 | 0.013807533 | -1.96 | 1.45 | 2.90 |
| ENSG00000256674 | known pseudogene | RP11-172C16.4 | 0.018953648 | -1.96 | 3.13 | 6.29 |
| ENSG00000146205 | anoctamin 7 | ANO7 | 0.015360657 | -1.96 | 1.24 | 2.45 |
| ENSG00000118432 | cannabinoid receptor 1 (brain) | CNR1 | 0.018604337 | -1.96 | 0.10 | 0.24 |
| ENSG00000176728 | testis-specific transcript, Y-linked 14 (non-protein coding) | TTTY14 | 0.000566486 | -1.97 | 0.10 | 0.48 |
| ENSG00000125144 | metallothionein 1G | MT1G | 3.40074E-09 | -1.97 | 25.85 | 96.79 |
| ENSG00000108272 | dehydrogenase/reductase (SDR family) member 11 | DHRS11 | 0.000705522 | -1.98 | 12.82 | 28.67 |
| ENSG00000146950 | shroom family member 2 | SHROOM2 | 0.041719001 | -1.98 | 0.41 | 0.80 |
| ENSG00000226268 | known pseudogene | RP11-61N20.3 | 0.00082939 | -1.99 | 3.28 | 8.18 |
| ENSG00000256802 | novel antisense | RP11-680F8.1 | 0.043154869 | -1.99 | 0.48 | 1.07 |
| ENSG00000198729 | protein phosphatase 1, regulatory (inhibitor) subunit 14C | PPP1R14C | 0.029373771 | -2.00 | 2.92 | 5.53 |
| ENSG00000156298 | tetraspanin 7 | TSPAN7 | 0.001403922 | -2.00 | 3.68 | 8.28 |
| ENSG00000147724 | family with sequence similarity 135, member B | FAM135B | 0.031756776 | -2.00 | 0.16 | 0.33 |
| ENSG00000148600 | cadherin-related family member 1 | CDHR1 | 0.00308326 | -2.00 | 1.73 | 3.75 |
| ENSG00000122035 | RAS-like, family 11, member A | RASL11A | 0.004102905 | -2.01 | 2.28 | 5.13 |
| ENSG00000113805 | contactin 3 (plasmacytoma associated) | CNTN3 | 0.010265963 | -2.01 | 0.85 | 1.78 |
| ENSG00000130203 | apolipoprotein E | APOE | 0.000399765 | -2.01 | 7.31 | 17.13 |
| ENSG00000234828 | novel lincRNA | RP11-526A4.1 | 0.002009314 | -2.01 | 0.75 | 1.92 |
| ENSG00000137872 | sema domain, transmembrane domain (TM), and cytoplasmic domai | SEMA6D | 0.008726689 | -2.02 | 2.60 | 5.21 |
| ENSG00000236576 | known pseudogene | RP11-22B10.3 | 0.048873619 | -2.03 | 1.00 | 2.23 |
| ENSG00000100092 | SH3-domain binding protein 1 | SH3BP1 | 0.004229496 | -2.03 | 3.26 | 6.72 |
| ENSG00000071991 | cadherin 19, type 2 | CDH19 | 0.027995337 | -2.05 | 0.13 | 0.32 |
| ENSG00000127249 | ATPase type 13A4 | ATP13A4 | 0.039605974 | -2.05 | 0.58 | 1.14 |
| ENSG00000225117 | arylsulfatase D pseudogene 1 | ARSDP1 | 0.035435519 | -2.05 | 0.77 | 1.88 |
| ENSG00000130038 | EF-hand calcium binding domain 4B | EFCAB4B | 0.022164156 | -2.05 | 1.41 | 2.64 |
| ENSG00000239205 | novel lincRNA | RP11-747D18.1 | 0.01506195 | -2.06 | 1.82 | 3.79 |
| ENSG00000159212 | chloride intracellular channel 6 | CLIC6 | 7.67932E-09 | -2.06 | 1.61 | 6.06 |
| ENSG00000170482 | solute carrier family 23 (nucleobase transporters), member 1 | SLC23A1 | 0.030270333 | -2.07 | 0.60 | 1.27 |
| ENSG00000106351 | ArfGAP with FG repeats 2 | AGFG2 | 0.022468511 | -2.09 | 3.82 | 7.11 |
| ENSG00000118557 | polyamine modulated factor 1 binding protein 1 | PMFBP1 | 0.035176077 | -2.10 | 0.38 | 0.80 |
| ENSG00000108242 | cytochrome P450, family 2, subfamily C, polypeptide 18 | CYP2C18 | 0.02357228 | -2.11 | 4.14 | 7.90 |
| ENSG00000162949 | calpain 13 | CAPN13 | 0.009083757 | -2.11 | 0.71 | 1.53 |
| ENSG00000231535 | long intergenic non-protein coding RNA 278 | LINC00278 | 1.28344E-05 | -2.12 | 1.18 | 4.69 |
| ENSG00000241224 | novel lincRNA | RP11-59E19.1 | 0.000300083 | -2.12 | 1.37 | 3.70 |
| ENSG00000218328 | chromosome 1 open reading frame 196 | C1orf196 | 0.044654908 | -2.13 | 0.55 | 1.35 |
| ENSG00000198650 | tyrosine aminotransferase | TAT | 0.009432402 | -2.13 | 0.16 | 0.48 |
| ENSG00000170382 | leucine rich repeat neuronal 2 | LRRN2 | 0.00235351 | -2.13 | 0.48 | 1.16 |
| ENSG00000173320 | storkhead box 2 | STOX2 | 0.01506195 | -2.14 | 0.31 | 0.72 |
| ENSG00000186205 | mitochondrial amidoxime reducing component 1 | MOSC1 | 0.002604314 | -2.14 | 2.08 | 4.51 |
| ENSG00000129824 | ribosomal protein S4, Y-linked 1 | RPS4Y1 | 0.000275726 | -2.15 | 7.28 | 17.60 |
| ENSG00000117983 | mucin 5B, oligomeric mucus/gel-forming | MUC5B | 0.007733723 | -2.15 | 3.27 | 6.53 |
| ENSG00000185432 | methyltransferase like 7A | METTL7A | 0.00026609 | -2.16 | 10.66 | 25.07 |
| ENSG00000225434 | novel lincRNA | RP11-63P12.6 | 0.034658886 | -2.16 | 0.61 | 1.32 |
| ENSG00000255545 | novel lincRNA | RP11-627G23.1 | 0.008157352 | -2.16 | 0.79 | 1.65 |
| ENSG00000127129 | endothelin 2 | EDN2 | 0.008849383 | -2.17 | 0.26 | 0.89 |
| ENSG00000099749 | taxilin gamma 2, pseudogene | TXLNG2P | 0.005504556 | -2.17 | 1.60 | 3.50 |
| ENSG00000189045 | ankyrin repeat and death domain containing 1B | ANKDD1B | 0.018197361 | -2.19 | 1.03 | 2.19 |
| ENSG00000116141 | MAP/microtubule affinity-regulating kinase 1 | MARK1 | 0.025188065 | -2.19 | 0.34 | 0.71 |
| ENSG00000188761 | BCL2-like 15 | BCL2L15 | 5.99986E-05 | -2.20 | 7.05 | 17.85 |
| ENSG00000137819 | progestin and adipoQ receptor family member V | PAQR5 | 0.000265264 | -2.21 | 2.65 | 6.46 |
| ENSG00000109819 | peroxisome proliferator-activated receptor gamma, coactivator 1 alp | PPARGC1A | 0.0005814 | -2.21 | 1.83 | 4.26 |
| ENSG00000185038 | HEAT repeat containing 7B1 | HEATR7B1 | 0.036895746 | -2.21 | 0.01 | 0.09 |
| ENSG00000126803 | heat shock 70kDa protein 2 | HSPA2 | 0.006241618 | -2.21 | 1.14 | 2.55 |
| ENSG00000186715 | macrophage stimulating 1 (hepatocyte growth factor-like) pseudogen | MST1P9 | 0.001077546 | -2.22 | 0.69 | 1.69 |
| ENSG00000150054 | membrane protein, palmitoylated 7 (MAGUK p55 subfamily member | MPP7 | 0.000505803 | -2.22 | 2.39 | 5.54 |
| ENSG00000156113 | potassium large conductance calcium-activated channel, subfamily M | KCNMA1 | 0.004616261 | -2.23 | 0.59 | 1.27 |
| ENSG00000072133 | ribosomal protein S6 kinase, 90kDa, polypeptide 6 | RPS6KA6 | 0.001352025 | -2.23 | 1.64 | 3.86 |
| ENSG00000172016 | regenerating islet-derived 3 alpha | REG3A | 0.002825625 | -2.24 | 5.23 | 1.84 |
| ENSG00000196620 | UDP glucuronosyltransferase 2 family, polypeptide B15 | UGT2B15 | 0.00011625 | -2.24 | 34.93 | 83.83 |
| ENSG00000128383 | apolipoprotein B mRNA editing enzyme, catalytic polypeptide-like 3A | APOBEC3A | 0.02215287 | -2.24 | 0.42 | 1.08 |
| ENSG00000115841 | family with sequence similarity 82, member A1 | FAM82A1 | 0.00186087 | -2.25 | 1.61 | 3.56 |
| ENSG00000198910 | L1 cell adhesion molecule | L1CAM | 0.021932336 | -2.26 | 0.15 | 0.36 |
| ENSG00000119938 | protein phosphatase 1, regulatory subunit 3C | PPP1R3C | 0.021003743 | -2.26 | 0.31 | 0.82 |
| ENSG00000172478 | chromosome 2 open reading frame 54 | C2orf54 | 0.036895746 | -2.26 | 0.10 | 0.34 |
| ENSG00000108984 | mitogen-activated protein kinase kinase 6 | MAP2K6 | 0.001005139 | -2.26 | 5.51 | 12.55 |
| ENSG00000152078 | transmembrane protein 56 | TMEM56 | 0.00106604 | -2.27 | 3.03 | 6.77 |
| ENSG00000184860 | short chain dehydrogenase/reductase family 42E, member 1 | SDR42E1 | 0.003710151 | -2.28 | 1.52 | 3.38 |
| ENSG00000155066 | prominin 2 | PROM2 | 0.000872292 | -2.28 | 1.49 | 3.45 |
| ENSG00000251370 | novel lincRNA | CTD-2201E9.1 | 0.014589402 | -2.28 | 0.67 | 1.64 |

| ENSG00000131773 | KH domain containing, RNA binding, signal transduction associated 3 | KHDRBS3 | 0.030659227 | -2.28 | 0.22 | 0.47 |
| --- | --- | --- | --- | --- | --- | --- |
| ENSG00000104177 | myelin expression factor 2 | MYEF2 | 0.004040933 | -2.28 | 0.60 | 1.40 |
| ENSG00000157005 | somatostatin | SST | 8.06174E-07 | -2.29 | 1.26 | 6.14 |
| ENSG00000112902 | sema domain, seven thrombospondin repeats (type 1 and type 1-like | SEMA5A | 0.040202309 | -2.29 | 3.03 | 5.42 |
| ENSG00000257023 | novel antisense | RP11-268P4.4 | 0.013426561 | -2.30 | 0.06 | 0.63 |
| ENSG00000188373 | chromosome 10 open reading frame 99 | C10orf99 | 1.14931E-06 | -2.30 | 30.08 | 89.69 |
| ENSG00000179915 | neurexin 1 | NRXN1 | 0.004019646 | -2.30 | 0.15 | 0.35 |
| ENSG00000167701 | glutamic-pyruvate transaminase (alanine aminotransferase) | GPT | 0.018089439 | -2.30 | 3.40 | 6.62 |
| ENSG00000228915 | known pseudogene | RP11-757C15.2 | 0.040014372 | -2.31 | 0.61 | 1.58 |
| ENSG00000256518 | known pseudogene | RP11-807H22.5 | 0.002677953 | -2.32 | 0.23 | 1.31 |
| ENSG00000231132 | novel antisense | RP11-40C11.2 | 0.045027559 | -2.32 | 1.18 | 3.15 |
| ENSG00000156284 | claudin 8 | CLDN8 | 0.018894382 | -2.32 | 0.38 | 0.99 |
| ENSG00000198944 | sosondowah ankyrin repeat domain family member A | ANKRD43 | 0.002073853 | -2.32 | 1.26 | 2.93 |
| ENSG00000249453 | novel antisense | RP13-497K6.1 | 0.023684052 | -2.33 | 1.46 | 3.94 |
| ENSG00000115263 | glucagon | GCG | 0.006569368 | -2.33 | 0.63 | 1.55 |
| ENSG00000090920 | Fc fragment of IgG binding protein | FCGBP | 0.000717904 | -2.33 | 45.24 | 103.11 |
| ENSG00000237476 | novel lincRNA | XXbac-B135H6.15 | 0.014535789 | -2.33 | 1.31 | 3.43 |
| ENSG00000162670 | family with sequence similarity 5, member C | FAM5C | 0.00328203 | -2.33 | 0.54 | 1.36 |
| ENSG00000246763 | novel antisense | CTC-463N11.3 | 0.013066022 | -2.33 | 0.89 | 2.04 |
| ENSG00000167107 | acyl-CoA synthetase family member 2 | ACSF2 | 0.00026609 | -2.33 | 2.59 | 6.21 |
| ENSG00000176387 | hydroxysteroid (11-beta) dehydrogenase 2 | HSD11B2 | 0.000214069 | -2.34 | 23.40 | 56.16 |
| ENSG00000250612 | known pseudogene | RP11-618I10.2 | 0.038082899 | -2.34 | 0.07 | 0.40 |
| ENSG00000101470 | troponin C type 2 (fast) | TNNC2 | 0.035602017 | -2.35 | 0.29 | 1.09 |
| ENSG00000197888 | UDP glucuronosyltransferase 2 family, polypeptide B17 | UGT2B17 | 2.90401E-05 | -2.35 | 79.71 | 202.73 |
| ENSG00000183773 | apoptosis-inducing factor, mitochondrion-associated, 3 | AIFM3 | 0.001015696 | -2.35 | 0.98 | 2.42 |
| ENSG00000244067 | glutathione S-transferase alpha 2 | GSTA2 | 0.040202309 | -2.36 | 0.31 | 0.96 |
| ENSG00000152763 | WD repeat domain 78 | WDR78 | 0.002229541 | -2.36 | 0.76 | 1.73 |
| ENSG00000168447 | sodium channel, non-voltage-gated 1, beta subunit | SCNN1B | 0.002105409 | -2.36 | 2.19 | 4.96 |
| ENSG00000237513 | novel lincRNA | RP11-325F22.2 | 0.006574565 | -2.36 | 1.10 | 2.87 |
| ENSG00000142920 | arginine decarboxylase | ADC | 0.0018831 | -2.36 | 0.43 | 1.05 |
| ENSG00000131096 | peptide YY | PYY | 0.018535666 | -2.36 | 0.71 | 1.89 |
| ENSG00000239783 | known pseudogene | RP5-1050K3.3 | 0.032848232 | -2.37 | 0.11 | 1.23 |
| ENSG00000033122 | leucine rich repeat containing 7 | LRRC7 | 0.028020734 | -2.37 | 0.04 | 0.14 |
| ENSG00000066468 | fibroblast growth factor receptor 2 | FGFR2 | 0.000557119 | -2.37 | 2.62 | 6.04 |
| ENSG00000244020 | known pseudogene | MT1P2 | 0.027070788 | -2.37 | 0.60 | 2.84 |
| ENSG00000228949 | UDP glucuronosyltransferase 1 family, polypeptide A12 pseudogene | UGT1A12P | 0.020283631 | -2.37 | 0.26 | 1.03 |
| ENSG00000012817 | lysine (K)-specific demethylase 5D | KDM5D | 1.74852E-05 | -2.38 | 0.82 | 2.30 |
| ENSG00000182648 | novel lincRNA | AC073871.2 | 0.001632745 | -2.38 | 1.26 | 3.96 |
| ENSG00000169918 | OTU domain containing 7A | OTUD7A | 0.003414075 | -2.38 | 0.25 | 0.67 |
| ENSG00000138109 | cytochrome P450, family 2, subfamily C, polypeptide 9 | CYP2C9 | 0.001646883 | -2.39 | 0.48 | 1.29 |
| ENSG00000088280 | ArfGAP with SH3 domain, ankyrin repeat and PH domain 3 | ASAP3 | 0.000166117 | -2.40 | 2.02 | 5.03 |
| ENSG00000186198 | solute carrier family 51, beta subunit | OSTBETA | 2.04473E-05 | -2.41 | 9.12 | 25.26 |
| ENSG00000172752 | collagen, type VI, alpha 5 | COL6A5 | 0.005085673 | -2.41 | 0.18 | 0.45 |
| ENSG00000147852 | very low density lipoprotein receptor | VLDLR | 0.000920216 | -2.42 | 0.41 | 1.17 |
| ENSG00000166165 | creatine kinase, brain | CKB | 0.005328276 | -2.42 | 31.83 | 64.10 |
| ENSG00000205364 | metallothionein 1M | MT1M | 2.79533E-05 | -2.43 | 3.43 | 11.65 |
| ENSG00000142583 | solute carrier family 2 (facilitated glucose/fructose transporter), mem | SLC2A5 | 0.026417104 | -2.43 | 0.16 | 0.37 |
| ENSG00000178568 | v-erb-a erythroblastic leukemia viral oncogene homolog 4 (avian) | ERBB4 | 0.038082899 | -2.43 | 0.01 | 0.05 |
| ENSG00000111052 | lin-7 homolog A (C. elegans) | LIN7A | 0.031756776 | -2.43 | 0.32 | 1.01 |
| ENSG00000173253 | doublesex and mab-3 related transcription factor 2 | DMRT2 | 0.030332981 | -2.44 | 0.21 | 0.56 |
| ENSG00000203858 | hydroxy-delta-5-steroid dehydrogenase, 3 beta, pseudogene 2 | HSD3BP2 | 0.012281265 | -2.44 | 0.14 | 0.62 |
| ENSG00000118777 | ATP-binding cassette, sub-family G (WHITE), member 2 | ABCG2 | 7.37016E-05 | -2.44 | 3.07 | 7.82 |
| ENSG00000157703 | SVOP-like | SVOPL | 0.002597243 | -2.45 | 0.09 | 0.31 |
| ENSG00000139540 | solute carrier family 39 (metal ion transporter), member 5 | SLC39A5 | 3.28793E-06 | -2.46 | 3.57 | 10.63 |
| ENSG00000256879 | novel antisense | RP11-284H19.1 | 0.044175696 | -2.46 | 1.26 | 5.63 |
| ENSG00000249170 | known pseudogene | RP11-1J11.1 | 0.008821962 | -2.46 | 1.52 | 3.70 |
| ENSG00000006740 | Rho GTPase activating protein 44 | ARHGAP44 | 0.000139379 | -2.47 | 2.70 | 6.77 |
| ENSG00000144619 | contactin 4 | CNTN4 | 0.000539871 | -2.48 | 0.57 | 1.42 |
| ENSG00000156345 | cyclin-dependent kinase 20 | CDK20 | 0.000120907 | -2.48 | 0.45 | 1.39 |
| ENSG00000104059 | family with sequence similarity 189, member A1 | FAM189A1 | 0.006608468 | -2.49 | 0.28 | 0.71 |
| ENSG00000240498 | CDKN2B antisense RNA 1 | CDKN2B-AS | 0.000623424 | -2.50 | 1.06 | 2.68 |
| ENSG00000152292 | SH2 domain containing 6 | SH2D6 | 0.029941331 | -2.50 | 0.18 | 0.47 |
| ENSG00000091138 | solute carrier family 26, member 3 | SLC26A3 | 2.07875E-05 | -2.51 | 127.97 | 336.24 |
| ENSG00000110887 | D-amino-acid oxidase | DAO | 0.016940011 | -2.51 | 0.06 | 0.41 |
| ENSG00000007216 | solute carrier family 13 (sodium-dependent dicarboxylate transporter | SLC13A2 | 0.001630043 | -2.51 | 0.24 | 0.76 |
| ENSG00000120498 | testis expressed 11 | TEX11 | 0.003482234 | -2.53 | 0.16 | 0.55 |
| ENSG00000249279 | putative processed transcript | CTC-436P18.3 | 0.038861727 | -2.53 | 0.13 | 0.63 |
| ENSG00000123612 | activin A receptor, type IC | ACVR1C | 6.80233E-05 | -2.53 | 0.73 | 2.15 |
| ENSG00000164659 | KIAA1324-like | KIAA1324L | 0.001123504 | -2.54 | 0.24 | 0.65 |
| ENSG00000188242 | Uncharacterized protein | CTD-2228K2.5 | 0.000107505 | -2.54 | 12.69 | 32.08 |
| ENSG00000198848 | carboxylesterase 1 | CES1 | 6.9825E-05 | -2.55 | 0.67 | 2.24 |
| ENSG00000135929 | cytochrome P450, family 27, subfamily A, polypeptide 1 | CYP27A1 | 0.000436911 | -2.57 | 1.07 | 2.80 |
| ENSG00000177103 | Down syndrome cell adhesion molecule like 1 | DSCAML1 | 0.046514162 | -2.58 | 0.03 | 0.11 |
| ENSG00000198948 | microfibrillar-associated protein 3-like | MFAP3L | 0.012671633 | -2.58 | 0.17 | 0.40 |
| ENSG00000139656 | small integral membrane protein 2 | C13orf44 | 0.035536733 | -2.59 | 0.55 | 1.72 |
| ENSG00000176771 | NCK-associated protein 5 | NCKAP5 | 0.00014973 | -2.59 | 0.47 | 1.25 |
| ENSG00000143416 | selenium binding protein 1 | SELENBP1 | 0.000607035 | -2.60 | 25.79 | 58.32 |
| ENSG00000104332 | secreted frizzled-related protein 1 | SFRP1 | 0.00679999 | -2.60 | 0.17 | 0.49 |
| ENSG00000103184 | SEC14-like 5 (S. cerevisiae) | SEC14L5 | 0.040202309 | -2.60 | 0.02 | 0.09 |
| ENSG00000228695 | carboxylesterase 1 pseudogene 1 | CES1P1 | 0.004929259 | -2.61 | 0.29 | 0.93 |
| ENSG00000163873 | glutamate receptor, ionotropic, kainate 3 | GRIK3 | 0.011104495 | -2.61 | 0.05 | 0.15 |
| ENSG00000148483 | transmembrane protein 236 | TMEM236 | 2.18162E-05 | -2.62 | 3.22 | 8.72 |
| ENSG00000245248 | novel antisense | RP11-305N23.1 | 0.038792869 | -2.62 | 0.04 | 0.17 |
| ENSG00000006047 | Y box binding protein 2 | YBX2 | 4.85097E-05 | -2.63 | 2.42 | 6.89 |
| ENSG00000184040 | known protein coding | FAM23B | 1.82515E-05 | -2.63 | 3.20 | 8.74 |
| ENSG00000219159 | Uncharacterized protein | AC011298.2 | 0.029463294 | -2.63 | 0.00 | 0.13 |
| ENSG00000257045 | novel antisense | RP11-486F17.1 | 0.001619715 | -2.64 | 2.41 | 6.61 |

| ENSG00000179750 | apolipoprotein B mRNA editing enzyme, catalytic polypeptide-like 3B | APOBEC3B | 0.000173738 | -2.64 | 2.00 | 5.43 |
| --- | --- | --- | --- | --- | --- | --- |
| ENSG00000135917 | solute carrier family 19, member 3 | SLC19A3 | 0.000304837 | -2.64 | 0.49 | 1.38 |
| ENSG00000205362 | metallothionein 1A | MT1A | 0.045306666 | -2.65 | 0.34 | 1.69 |
| ENSG00000171208 | neuropilin (NRP) and tolloid (TLL)-like 2 | NETO2 | 0.000244295 | -2.65 | 2.27 | 5.58 |
| ENSG00000169271 | heat shock 27kDa protein 3 | HSPB3 | 0.00391776 | -2.65 | 0.04 | 0.69 |
| ENSG00000183463 | parahox cluster neighbor | PRHOXNB | 0.004216089 | -2.66 | 1.04 | 3.45 |
| ENSG00000248114 | known pseudogene | AC114812.9 | 0.000670277 | -2.67 | 1.09 | 3.61 |
| ENSG00000133742 | carbonic anhydrase I | CA1 | 4.66107E-06 | -2.67 | 13.51 | 38.48 |
| ENSG00000138435 | cholinergic receptor, nicotinic, alpha 1 (muscle) | CHRNA1 | 0.017804534 | -2.69 | 0.05 | 0.27 |
| ENSG00000124253 | phosphoenolpyruvate carboxykinase 1 (soluble) | PCK1 | 1.39576E-05 | -2.69 | 5.09 | 14.00 |
| ENSG00000214357 | neuralized homolog 1B (Drosophila) | NEURL1B | 0.000210313 | -2.69 | 2.35 | 5.81 |
| ENSG00000203401 | novel pseudogene | AC009061.1 | 6.00719E-05 | -2.69 | 5.49 | 14.40 |
| ENSG00000163959 | solute carrier family 51, alpha subunit | AC069257.9 | 1.11632E-05 | -2.70 | 1.70 | 4.87 |
| ENSG00000204099 | sialidase 4 | NEU4 | 0.012314676 | -2.70 | 0.62 | 1.36 |
| ENSG00000231412 | novel lincRNA | AC005392.8 | 0.002435125 | -2.71 | 1.32 | 12.06 |
| ENSG00000249473 |  | AC123912.1 | 0.013331684 | -2.71 | 0.08 | 0.45 |
| ENSG00000142959 | bestrophin 4 | BEST4 | 0.001506811 | -2.71 | 1.70 | 4.09 |
| ENSG00000164270 | 5-hydroxytryptamine (serotonin) receptor 4, G protein-coupled | HTR4 | 0.000228967 | -2.71 | 0.33 | 0.92 |
| ENSG00000241119 | UDP glucuronosyltransferase 1 family, polypeptide A9 | UGT1A9 | 0.000337129 | -2.71 | 2.39 | 6.62 |
| ENSG00000170927 | polycystic kidney and hepatic disease 1 (autosomal recessive) | PKHD1 | 0.004304778 | -2.72 | 0.07 | 0.18 |
| ENSG00000162992 | neuronal differentiation 1 | NEUROD1 | 0.000212079 | -2.74 | 0.10 | 0.53 |
| ENSG00000165841 | cytochrome P450, family 2, subfamily C, polypeptide 19 | CYP2C19 | 2.41374E-05 | -2.74 | 0.81 | 2.56 |
| ENSG00000233377 | MT-ND4 pseudogene 20 | MTND4P20 | 0.006771885 | -2.76 | 0.94 | 2.63 |
| ENSG00000119121 | transient receptor potential cation channel, subfamily M, member 6 | TRPM6 | 3.22445E-05 | -2.76 | 1.48 | 4.03 |
| ENSG00000233445 | ribosomal protein L17 pseudogene 11 | RPL17P11 | 0.001328582 | -2.77 | 1.40 | 4.51 |
| ENSG00000184709 | leucine rich repeat containing 26 | LRRC26 | 6.80233E-05 | -2.77 | 1.39 | 4.39 |
| ENSG00000012504 | nuclear receptor subfamily 1, group H, member 4 | NR1H4 | 1.88583E-06 | -2.78 | 2.31 | 7.32 |
| ENSG00000108381 | aspartoacylase | ASPA | 0.000604828 | -2.79 | 0.26 | 0.76 |
| ENSG00000224814 | novel antisense | AC114812.8 | 0.007006164 | -2.79 | 2.22 | 6.89 |
| ENSG00000236028 | novel lincRNA | RP11-323C15.2 | 0.00367282 | -2.81 | 0.08 | 1.22 |
| ENSG00000141434 | meprin A, beta | MEP1B | 5.1251E-07 | -2.81 | 3.53 | 11.37 |
| ENSG00000197415 | ventricular zone expressed PH domain homolog 1 (zebrafish) | VEPH1 | 0.019560727 | -2.81 | 0.05 | 0.17 |
| ENSG00000139209 | solute carrier family 38, member 4 | SLC38A4 | 6.64193E-08 | -2.82 | 1.08 | 3.98 |
| ENSG00000168748 | carbonic anhydrase VII | CA7 | 0.006755198 | -2.82 | 2.09 | 4.51 |
| ENSG00000153707 | protein tyrosine phosphatase, receptor type, D | PTPRD | 0.040996115 | -2.82 | 1.57 | 2.79 |
| ENSG00000246090 | novel lincRNA | RP11-696N14.1 | 5.2072E-05 | -2.83 | 0.40 | 1.18 |
| ENSG00000184313 | HEAT repeat containing 8 | HEATR8 | 0.000304837 | -2.85 | 0.50 | 1.29 |
| ENSG00000179314 | WSC domain containing 1 | WSCD1 | 1.23856E-05 | -2.86 | 0.93 | 2.74 |
| ENSG00000248705 | known pseudogene | AC114812.10 | 0.000909852 | -2.86 | 0.94 | 3.16 |
| ENSG00000196482 | estrogen-related receptor gamma | ESRRG | 0.00025952 | -2.88 | 0.11 | 0.35 |
| ENSG00000166183 | asparaginase homolog (S. cerevisiae) | ASPG | 0.00485396 | -2.88 | 0.23 | 0.73 |
| ENSG00000230873 | uncharacterized protein LOC401236 | RP3-486B10.1 | 0.002268308 | -2.89 | 0.06 | 0.37 |
| ENSG00000104267 | carbonic anhydrase II | CA2 | 4.04887E-07 | -2.89 | 78.61 | 242.32 |
| ENSG00000196616 | alcohol dehydrogenase 1B (class I), beta polypeptide | ADH1B | 6.98939E-07 | -2.89 | 2.85 | 8.72 |
| ENSG00000172986 | glucoside xylosyltransferase 2 | GXYLT2 | 5.05413E-05 | -2.89 | 1.09 | 3.11 |
| ENSG00000136315 | novel lincRNA | RP11-84C10.2 | 0.023412209 | -2.90 | 0.20 | 1.05 |
| ENSG00000243135 | UDP glucuronosyltransferase 1 family, polypeptide A3 | UGT1A3 | 0.000416428 | -2.92 | 1.32 | 4.08 |
| ENSG00000123560 | proteolipid protein 1 | PLP1 | 2.46468E-05 | -2.93 | 0.20 | 0.72 |
| ENSG00000070193 | fibroblast growth factor 10 | FGF10 | 0.017699635 | -2.94 | 0.08 | 0.34 |
| ENSG00000115457 | insulin-like growth factor binding protein 2, 36kDa | IGFBP2 | 0.000135342 | -2.96 | 2.27 | 5.66 |
| ENSG00000169432 | sodium channel, voltage-gated, type IX, alpha subunit | SCN9A | 5.99628E-06 | -2.98 | 0.33 | 1.05 |
| ENSG00000177992 | family with sequence similarity 75, member E1 | C9orf79 | 0.003790234 | -2.98 | 0.07 | 0.28 |
| ENSG00000256643 | novel lincRNA | RP11-349K16.1 | 2.49575E-08 | -2.99 | 2.24 | 10.74 |
| ENSG00000225354 | known pseudogene | RP11-400G3.3 | 0.006121677 | -2.99 | 0.13 | 0.86 |
| ENSG00000183034 | otopetrin 2 | OTOP2 | 2.33942E-05 | -3.01 | 0.84 | 2.78 |
| ENSG00000196517 | solute carrier family 6 (neurotransmitter transporter, glycine), memb | SLC6A9 | 7.70036E-05 | -3.01 | 1.15 | 3.11 |
| ENSG00000236404 | novel lincRNA | RP11-125B21.2 | 0.026426528 | -3.03 | 0.06 | 0.23 |
| ENSG00000135097 | musashi homolog 1 (Drosophila) | MSI1 | 0.000364647 | -3.04 | 0.21 | 0.80 |
| ENSG00000125851 | proprotein convertase subtilisin/kexin type 2 | PCSK2 | 0.011077886 | -3.05 | 0.04 | 0.15 |
| ENSG00000180616 | somatostatin receptor 2 | SSTR2 | 0.000531326 | -3.05 | 0.17 | 0.65 |
| ENSG00000089199 | chromogranin B (secretogranin 1) | CHGB | 2.46093E-26 | -3.06 | 0.50 | 6.72 |
| ENSG00000142515 | kallikrein-related peptidase 3 | KLK3 | 0.004128794 | -3.06 | 0.13 | 0.55 |
| ENSG00000082397 | erythrocyte membrane protein band 4.1-like 3 | EPB41L3 | 4.83449E-08 | -3.10 | 2.53 | 8.71 |
| ENSG00000240224 | UDP glucuronosyltransferase 1 family, polypeptide A5 | UGT1A5 | 0.000928407 | -3.10 | 1.17 | 4.62 |
| ENSG00000132854 | KN motif and ankyrin repeat domains 4 | KANK4 | 0.000466929 | -3.10 | 0.07 | 0.28 |
| ENSG00000144290 | solute carrier family 4, sodium bicarbonate transporter, member 10 | SLC4A10 | 5.81242E-07 | -3.12 | 0.51 | 1.76 |
| ENSG00000167165 | UDP glucuronosyltransferase 1 family, polypeptide A6 | UGT1A6 | 0.0001351 | -3.13 | 1.04 | 3.10 |
| ENSG00000135083 | cyclin J-like | CCNJL | 3.79836E-06 | -3.15 | 0.81 | 2.59 |
| ENSG00000139163 | ethanolamine kinase 1 | ETNK1 | 5.80812E-06 | -3.15 | 22.38 | 61.63 |
| ENSG00000087250 | metallothionein 3 | MT3 | 0.033734346 | -3.15 | 0.05 | 0.61 |
| ENSG00000167434 | carbonic anhydrase IV | CA4 | 1.56196E-10 | -3.15 | 14.73 | 60.77 |
| ENSG00000225953 | SATB2 antisense RNA 1 | AC017096.1 | 1.90132E-06 | -3.15 | 1.86 | 5.80 |
| ENSG00000233729 | novel antisense | AC016909.1 | 0.030319518 | -3.17 | 0.05 | 0.31 |
| ENSG00000250137 | novel antisense | RP11-380P13.1 | 0.000199603 | -3.18 | 0.67 | 2.88 |
| ENSG00000203857 | hydroxy-delta-5-steroid dehydrogenase, 3 beta- and steroid delta-iso | HSD3B1 | 0.004351067 | -3.18 | 0.10 | 0.47 |
| ENSG00000244122 | UDP glucuronosyltransferase 1 family, polypeptide A7 | UGT1A7 | 3.89485E-06 | -3.18 | 1.23 | 4.07 |
| ENSG00000132915 | phosphodiesterase 6A, cGMP-specific, rod, alpha | PDE6A | 4.34627E-06 | -3.20 | 0.28 | 0.97 |
| ENSG00000111846 | glucosaminyl (N-acetyl) transferase 2, I-branching enzyme (I blood g | GCNT2 | 5.04491E-08 | -3.21 | 1.01 | 3.54 |
| ENSG00000169676 | dopamine receptor D5 | DRD5 | 0.000282344 | -3.22 | 0.14 | 0.68 |
| ENSG00000175356 | signal peptide, CUB domain, EGF-like 2 | SCUBE2 | 6.76765E-07 | -3.22 | 0.31 | 1.14 |
| ENSG00000253549 | novel antisense | RP11-317J10.2 | 3.59072E-06 | -3.23 | 1.64 | 5.90 |
| ENSG00000139364 | transmembrane protein 132B | TMEM132B | 0.002660808 | -3.23 | 0.01 | 0.06 |
| ENSG00000205358 | metallothionein 1H | MT1H | 0.000243125 | -3.24 | 2.56 | 8.50 |
| ENSG00000179455 | makorin ring finger protein 3 | MKRN3 | 0.003949991 | -3.25 | 0.02 | 0.18 |
| ENSG00000071203 | membrane-spanning 4-domains, subfamily A, member 12 | MS4A12 | 1.17951E-09 | -3.25 | 9.84 | 38.92 |
| ENSG00000245750 | novel lincRNA | RP11-279F6.1 | 6.13304E-08 | -3.26 | 0.32 | 1.95 |
| ENSG00000100604 | chromogranin A (parathyroid secretory protein 1) | CHGA | 1.13045E-16 | -3.27 | 9.79 | 60.16 |

| ENSG00000203685 | chromosome 1 open reading frame 95 | C1orf95 | 0.006940836 | -3.28 | 0.06 | 0.20 |
| --- | --- | --- | --- | --- | --- | --- |
| ENSG00000187783 | transmembrane protein 72 | TMEM72 | 7.1476E-06 | -3.29 | 0.42 | 1.57 |
| ENSG00000234143 | UDP glucuronosyltransferase 1 family, polypeptide A13 pseudogene | UGT1A13P | 0.000163757 | -3.29 | 1.09 | 3.87 |
| ENSG00000242515 | UDP glucuronosyltransferase 1 family, polypeptide A10 | UGT1A10 | 1.18659E-06 | -3.30 | 14.96 | 45.57 |
| ENSG00000172572 | phosphodiesterase 3A, cGMP-inhibited | PDE3A | 1.2115E-06 | -3.31 | 1.31 | 4.11 |
| ENSG00000171126 | potassium voltage-gated channel, subfamily G, member 3 | KCNG3 | 9.66574E-05 | -3.32 | 0.25 | 0.88 |
| ENSG00000152785 | bone morphogenetic protein 3 | BMP3 | 1.22871E-07 | -3.32 | 0.99 | 3.49 |
| ENSG00000155850 | solute carrier family 26 (sulfate transporter), member 2 | SLC26A2 | 1.47271E-07 | -3.33 | 40.75 | 132.09 |
| ENSG00000134240 | 3-hydroxy-3-methylglutaryl-CoA synthase 2 (mitochondrial) | HMGCS2 | 2.75324E-07 | -3.36 | 42.36 | 131.75 |
| ENSG00000188175 | HEPACAM family member 2 | HEPACAM2 | 0.000851563 | -3.36 | 5.08 | 11.61 |
| ENSG00000175497 | dipeptidyl-peptidase 10 (non-functional) | DPP10 | 3.22729E-05 | -3.36 | 0.13 | 0.45 |
| ENSG00000036672 | ubiquitin specific peptidase 2 | USP2 | 3.20477E-08 | -3.37 | 1.25 | 4.64 |
| ENSG00000196090 | protein tyrosine phosphatase, receptor type, T | PTPRT | 0.003277501 | -3.39 | 0.02 | 0.09 |
| ENSG00000187758 | alcohol dehydrogenase 1A (class I), alpha polypeptide | ADH1A | 6.57947E-06 | -3.39 | 1.26 | 4.14 |
| ENSG00000099715 | protocadherin 11 Y-linked | PCDH11Y | 0.002020712 | -3.39 | 0.05 | 0.16 |
| ENSG00000039987 | bestrophin 2 | BEST2 | 1.22077E-06 | -3.40 | 1.21 | 4.34 |
| ENSG00000107105 | ELAV (embryonic lethal, abnormal vision, Drosophila)-like 2 (Hu anti | ELAVL2 | 0.001803725 | -3.40 | 0.04 | 0.20 |
| ENSG00000066230 | solute carrier family 9, subfamily A (NHE3, cation proton antiporter 3 | SLC9A3 | 1.27253E-09 | -3.40 | 20.64 | 79.44 |
| ENSG00000145864 | gamma-aminobutyric acid (GABA) A receptor, beta 2 | GABRB2 | 0.005360912 | -3.41 | 0.05 | 0.18 |
| ENSG00000249948 | glucosidase, beta, acid 3 (cytosolic) | GBA3 | 8.35474E-10 | -3.43 | 1.20 | 5.28 |
| ENSG00000250376 | known pseudogene | CTD-2005D20.1 | 0.006643385 | -3.44 | 0.24 | 1.25 |
| ENSG00000165794 | solute carrier family 39 (zinc transporter), member 2 | SLC39A2 | 0.001994565 | -3.46 | 0.16 | 0.83 |
| ENSG00000113249 | hepatitis A virus cellular receptor 1 | HAVCR1 | 6.64008E-09 | -3.47 | 0.70 | 3.36 |
| ENSG00000047936 | c-ros oncogene 1 , receptor tyrosine kinase | ROS1 | 8.86941E-05 | -3.48 | 0.02 | 0.16 |
| ENSG00000176884 | glutamate receptor, ionotropic, N-methyl D-aspartate 1 | GRIN1 | 5.65931E-06 | -3.48 | 0.22 | 0.82 |
| ENSG00000178836 | known pseudogene | AC114812.5 | 0.000117488 | -3.48 | 1.32 | 5.62 |
| ENSG00000158813 | ectodysplasin A | EDA | 4.41635E-05 | -3.50 | 0.13 | 0.49 |
| ENSG00000147257 | glypican 3 | GPC3 | 9.89072E-05 | -3.50 | 0.52 | 1.69 |
| ENSG00000175600 | chromosome 7 open reading frame 10 | C7orf10 | 4.16445E-07 | -3.57 | 0.32 | 1.38 |
| ENSG00000102349 | Kruppel-like factor 8 | KLF8 | 2.69745E-07 | -3.57 | 0.45 | 1.58 |
| ENSG00000129167 | tryptophan hydroxylase 1 | TPH1 | 5.40273E-08 | -3.58 | 0.58 | 2.26 |
| ENSG00000162409 | protein kinase, AMP-activated, alpha 2 catalytic subunit | PRKAA2 | 4.43112E-05 | -3.62 | 0.10 | 0.36 |
| ENSG00000227802 | DnaJ (Hsp40) homolog, subfamily B, member 3 | DNAJB3 | 3.89965E-06 | -3.64 | 0.63 | 2.74 |
| ENSG00000197444 | oxoglutarate dehydrogenase-like | OGDHL | 0.001987644 | -3.68 | 0.01 | 0.13 |
| ENSG00000172238 | atonal homolog 1 (Drosophila) | ATOH1 | 2.7242E-05 | -3.68 | 2.89 | 8.50 |
| ENSG00000164199 | G protein-coupled receptor 98 | GPR98 | 9.8196E-08 | -3.68 | 0.10 | 0.39 |
| ENSG00000242366 | UDP glucuronosyltransferase 1 family, polypeptide A8 | UGT1A8 | 1.71153E-07 | -3.69 | 6.15 | 21.29 |
| ENSG00000228445 | UDP glucuronosyltransferase 1 family, polypeptide A2 pseudogene | UGT1A2P | 1.77688E-05 | -3.72 | 0.80 | 3.39 |
| ENSG00000108576 | solute carrier family 6 (neurotransmitter transporter, serotonin), me | SLC6A4 | 7.94114E-06 | -3.72 | 0.06 | 0.35 |
| ENSG00000182271 | transmembrane and immunoglobulin domain containing 1 | TMIGD1 | 2.00699E-11 | -3.73 | 5.97 | 27.71 |
| ENSG00000218274 | known pseudogene | RP3-407E4.3 | 5.80812E-06 | -3.76 | 0.19 | 1.23 |
| ENSG00000249763 | known pseudogene | RP11-618I10.1 | 0.000195168 | -3.81 | 0.18 | 1.02 |
| ENSG00000164794 | potassium channel, subfamily V, member 1 | KCNV1 | 0.000157201 | -3.81 | 0.09 | 0.37 |
| ENSG00000233215 | novel lincRNA | AP000472.2 | 7.06889E-12 | -3.82 | 0.66 | 4.55 |
| ENSG00000176399 | DMRT-like family A1 | DMRTA1 | 0.000197878 | -3.85 | 0.14 | 0.71 |
| ENSG00000100505 | tripartite motif containing 9 | TRIM9 | 2.40916E-05 | -3.86 | 0.05 | 0.25 |
| ENSG00000166869 | calcineurin-like EF hand protein 2 | AC130454.2 | 4.31228E-11 | -3.91 | 10.65 | 45.86 |
| ENSG00000164128 | neuropeptide Y receptor Y1 | NPY1R | 8.30434E-05 | -3.92 | 0.52 | 1.59 |
| ENSG00000250328 | novel antisense | CTC-210G5.1 | 0.018715141 | -4.02 | 0.63 | 1.48 |
| ENSG00000124615 | molybdenum cofactor synthesis 1 | MOCS1 | 5.13992E-08 | -4.03 | 0.71 | 2.77 |
| ENSG00000016490 | chloride channel accessory 1 | CLCA1 | 0.008706173 | -4.05 | 160.41 | 308.73 |
| ENSG00000111863 | androgen-dependent TFPI-regulating protein | C6orf105 | 1.08928E-08 | -4.05 | 4.71 | 16.98 |
| ENSG00000240995 | known pseudogene | RP11-708H21.1 | 0.000105655 | -4.07 | 1.05 | 4.60 |
| ENSG00000103375 | aquaporin 8 | AQP8 | 6.57019E-15 | -4.07 | 27.24 | 151.88 |
| ENSG00000179520 | solute carrier family 17 (sodium-dependent inorganic phosphate cotr | SLC17A8 | 1.78396E-05 | -4.11 | 0.05 | 0.37 |
| ENSG00000109182 | cell wall biogenesis 43 C-terminal homolog (S. cerevisiae) | CWH43 | 1.98529E-08 | -4.20 | 1.04 | 4.15 |
| ENSG00000174992 | zymogen granule protein 16 homolog (rat) | ZG16 | 1.45039E-11 | -4.21 | 75.83 | 333.05 |
| ENSG00000248144 | alcohol dehydrogenase 1C (class I), gamma polypeptide | ADH1C | 7.29312E-10 | -4.22 | 33.56 | 128.06 |
| ENSG00000206159 | glycogenin 2 pseudogene 1 | GYG2P1 | 0.006777643 | -4.24 | 0.33 | 0.98 |
| ENSG00000138669 | protein kinase, cGMP-dependent, type II | PRKG2 | 1.0123E-07 | -4.35 | 0.32 | 1.46 |
| ENSG00000254645 | novel lincRNA | RP11-396O20.2 | 7.38219E-10 | -4.44 | 1.23 | 6.73 |
| ENSG00000244474 | UDP glucuronosyltransferase 1 family, polypeptide A4 | UGT1A4 | 2.03403E-07 | -4.50 | 1.01 | 4.77 |
| ENSG00000204936 | CD177 molecule | CD177 | 2.19807E-13 | -4.52 | 12.11 | 61.19 |
| ENSG00000131482 | glucose-6-phosphatase, catalytic subunit | G6PC | 7.97921E-05 | -4.55 | 0.01 | 0.23 |
| ENSG00000164093 | paired-like homeodomain 2 | PITX2 | 4.465E-10 | -4.56 | 1.41 | 5.95 |
| ENSG00000204933 | Uncharacterized protein | AC005392.1 | 1.98439E-14 | -4.67 | 11.52 | 69.69 |
| ENSG00000064655 | eyes absent homolog 2 (Drosophila) | EYA2 | 2.14952E-09 | -4.68 | 0.64 | 2.93 |
| ENSG00000122756 | ciliary neurotrophic factor receptor | CNTFR | 5.48554E-10 | -4.69 | 0.56 | 3.12 |
| ENSG00000224968 | novel lincRNA | RP1-35C21.1 | 7.99264E-09 | -4.97 | 0.76 | 6.04 |
| ENSG00000203859 | hydroxy-delta-5-steroid dehydrogenase, 3 beta- and steroid delta-iso | HSD3B2 | 8.71995E-16 | -5.29 | 0.95 | 6.90 |
| ENSG00000225421 | novel lincRNA | AC019330.1 | 5.45023E-07 | -5.30 | 0.40 | 3.54 |
| ENSG00000135220 | UDP glucuronosyltransferase 2 family, polypeptide A3 | UGT2A3 | 4.56633E-15 | -5.58 | 3.31 | 19.32 |
| ENSG00000197991 | protocadherin 20 | PCDH20 | 1.97719E-08 | -5.81 | 0.40 | 1.64 |
| ENSG00000196660 | solute carrier family 30, member 10 | SLC30A10 | 1.99745E-16 | -6.15 | 0.95 | 6.43 |
| ENSG00000203306 | novel pseudogene | AP001007.1 | 1.09919E-16 | -6.27 | 1.06 | 9.21 |
| ENSG00000132429 | popeye domain containing 3 | POPDC3 | 1.56202E-07 | -6.33 | 0.14 | 1.19 |
| ENSG00000167080 | beta-1,4-N-acetyl-galactosaminyl transferase 2 | B4GALNT2 | 5.29903E-14 | -7.70 | 9.25 | 48.64 |
| ENSG00000183844 | family with sequence similarity 3, member B | FAM3B | 9.55232E-15 | -8.23 | 0.74 | 4.84 |
| ENSG00000134955 | solute carrier family 37 (glycerol-3-phosphate transporter), member | SLC37A2 | 2.77847E-30 | -12.92 | 1.87 | 26.62 |
| ENSG00000132874 | solute carrier family 14 (urea transporter), member 2 | SLC14A2 | 3.4853E-39 | -18.67 | 0.33 | 9.38 |

**Table S5. BRAF V600E mutations in SSA/Ps and uninvolved colon from patients with serrated polyposis syndrome.** Sequencing of a 700 bp PCR amplicon of BRAF, that included codon 600, was done on samples (20 SSA/Ps and patient matched uninvolved controls) from twelve serrated polyposis patients. PCR products were sequenced (both strands) using an Applied Biosystems 3130 Genetic Analyzer and mutations were identified using Mutation Surveyor software (see SI Materials and Methods). Hyperplastic polyps and patient matched uninvolved colon (five patients) were also analyzed and showed no V600E BRAF mutations.

| **Tissue** | **Number of Samples** | **BRAF V600E (%)** |
| --- | --- | --- |
| **Patient matched uninvolved** | 16 | 0 (0) |
| **colon** |  |  |
| **SSA/Ps** | 20 | 12 (60) |
| **Hyperplastic polyps** | 10 | 0 (0) |
|  | | |
| **Size** | | |
| **Large SSA/Ps ( ≥ 1 cm)** | 10 | 7 (70) |
| **Small SSA/Ps ( < 1 cm)** | 10 | 5 (50) |

**Figure S1. Expression of adolase B (*ALDOB*) mRNA in SSA/Ps, adenomatous polyps (Adenoma) and controls. Panel A.** *ALDOB* RNA sequencing results. The y-axis represents RPKM. The x-axis represents the coordinates and gene structure of the *ALDOB* transcript. Bioinformatic analysis revealed a 20-fold increase in *ALDOB* mRNA in SSA/Ps (red, n=7 polyps) compared to controls (blue and green). **Panel B.** Relative mRNA levels of *ALDOB* in small and large SSA/Ps n=21), adenomatous polyps (n=10), right uninvolved colon of

serrated polyposis syndrome patients (n=10) and control right colon (screening colonoscopy with no polyps; (n=10) were measured by qPCR relative to β-actin. In small and large SSA/Ps *ALDOB* expression was greater by 33 and 38-fold, respectively, compared to controls.

A **ALDOB**


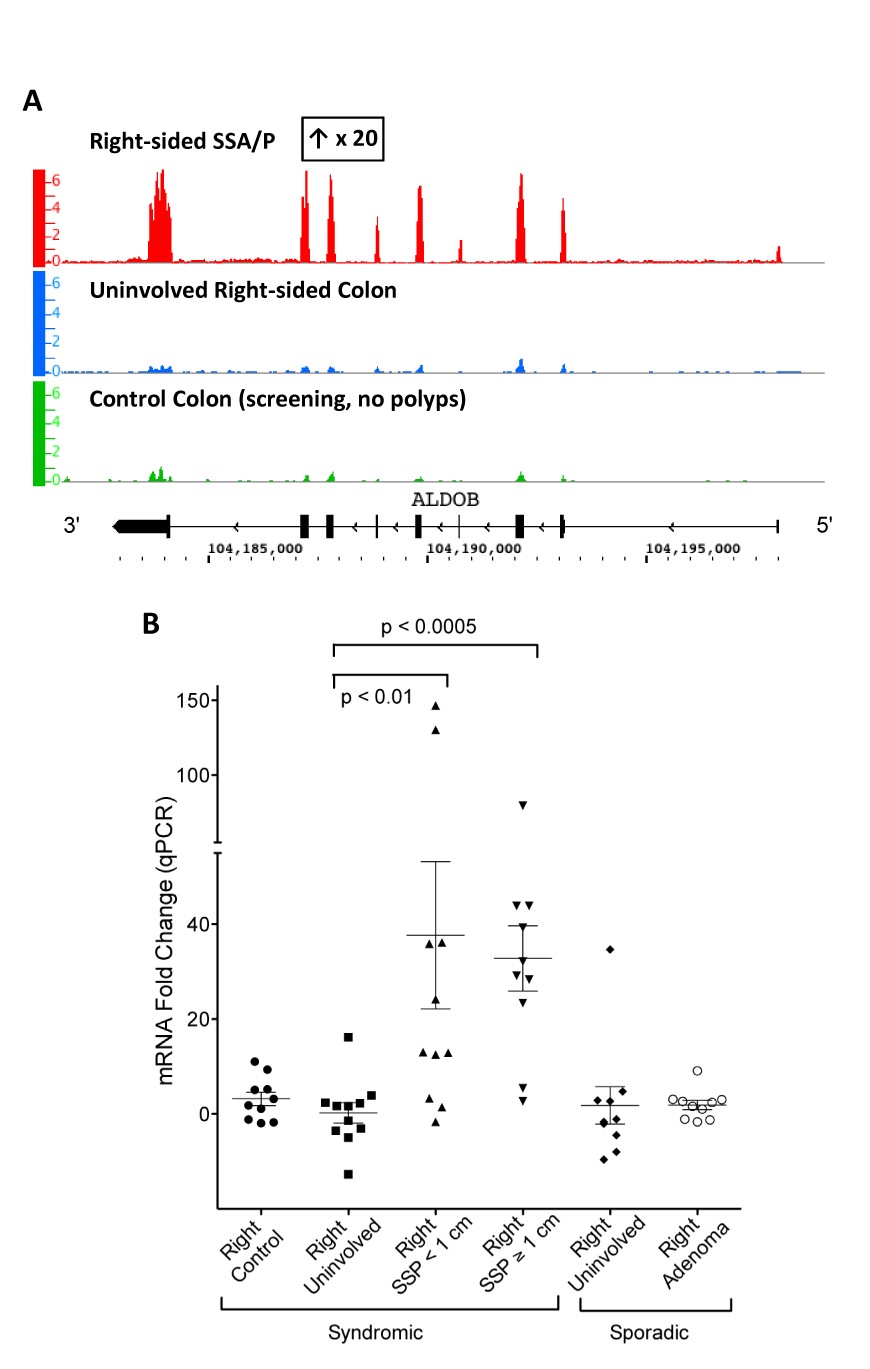


**SSA/P**

x 20

**Uninvolved Colon**

**Control Colon (screening, no polyps)**

**ALDOB**

B

mRNA Fold Change (qPCR)

Syndromic Sporadic

**Figure S2. Immunostaining for REG4 in control colon, SSA/Ps, hyperplastic and adenomatous polyps and higher magnification view of VSIG1 staining of an SSA/P.** Representative images of immunoperoxidase staining with affinity purified polyclonal antibodies and formalin-fixed, paraffin- embedded biopsies of control colon (Panel A, n=15), syndromic SSA/Ps (Panel B, n=9), sporadic SSA/Ps (Panel C, n=15), hyperplastic polyps (Panel D, n=10) and adenomatous polyps (Panel E, n=10) are shown. Immunostaining methods are described in detail in Methods. A representative higher magnification view of VSIG1 immunostaining of an SSA/P is shown (Panel F).


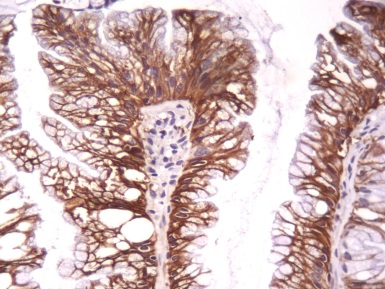
**REG4**


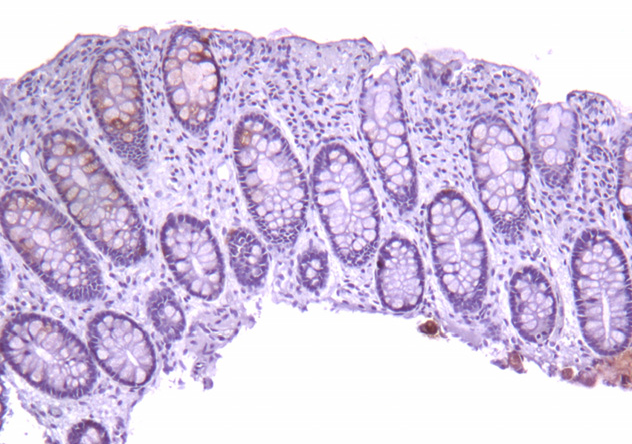

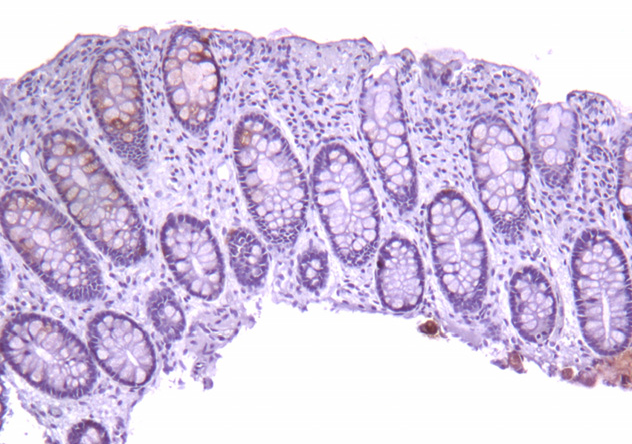


# A F

**B**
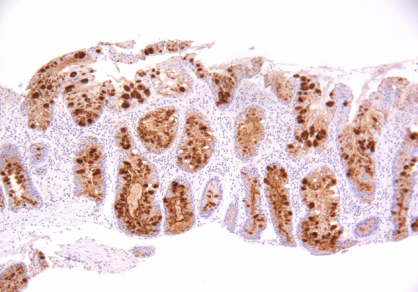


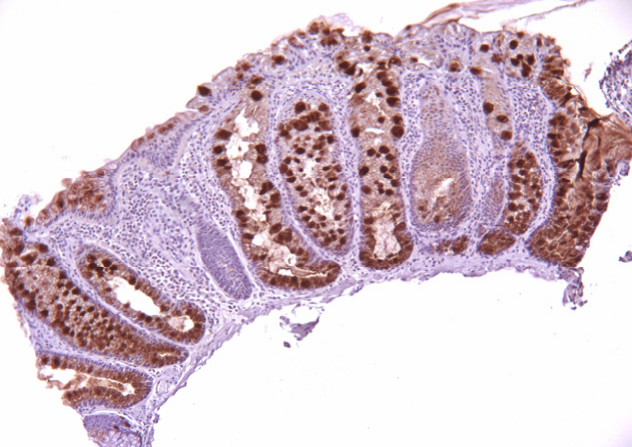

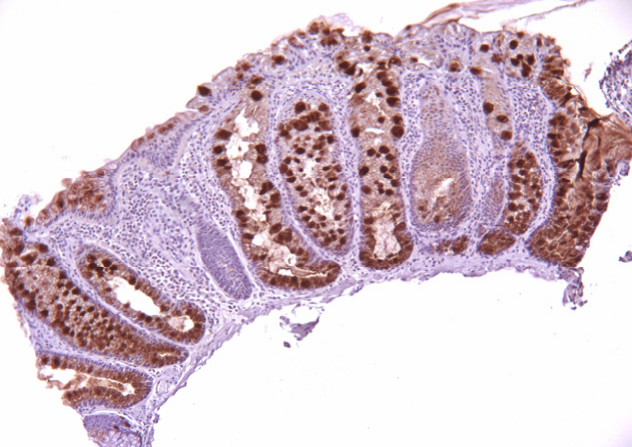


**C**


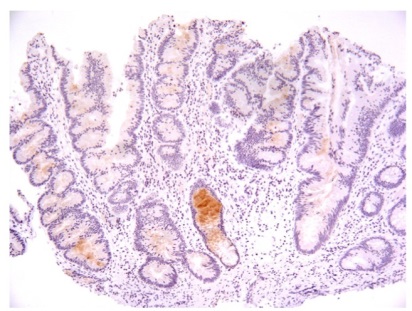
**D**
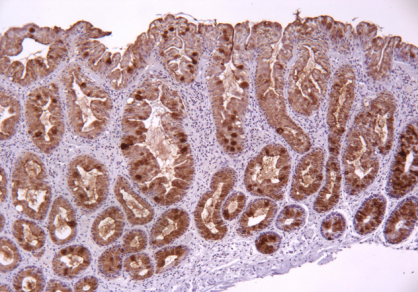


**E**

**VSIG1**
